# Transcriptional profiling reveals the role of *Candida albicans* Rap1 in oxidative stress response

**DOI:** 10.1042/BSR20240689

**Published:** 2024-12-12

**Authors:** Wen-Han Wang, Hsuan-Yu Chen, Sheng-Yuan Chen, Chung-Yu Lan

**Affiliations:** 1 Institute of Molecular and Cellular Biology, National Tsing Hua University, Hsinchu 300044, Taiwan; 2 Department of Life Science, National Tsing Hua University, Hsinchu 300044, Taiwan; 3 School of Medicine, National Tsing Hua University, Hsinchu 300044, Taiwan

**Keywords:** Candida albicans, oxidative stress, Rap1

## Abstract

*Candida albicans* is a member of the human commensal microbiota but can also cause opportunistic infections, including life-threatening invasive candidiasis, particularly in immunocompromised patients. One of the important features of *C. albicans* commensalism and virulence is its ability to adapt to diverse environmental stress conditions within the host. Rap1 is a DNA-binding protein identified in yeasts, protozoa, and mammalian cells, and it plays multiple functions, including telomere regulation. Intriguingly, our previous study showed that Rap1 is also involved in cell wall integrity, biofilm formation, and virulence in *C. albicans*. In this work, using RNA-seq analysis and other approaches, the role of *C. albicans* Rap1 in oxidative stress response was further revealed. The *RAP1*-deletion mutant exhibited greater resistance to the superoxide generator menadione, a lower level of intracellular reactive oxygen species (ROS) upon menadione treatment, and higher expression levels of superoxide dismutase genes, all in response to oxidative stress. Moreover, the association between Rap1-mediated oxidative stress response and the mitogen-activated protein kinase (MAPK) Hog1, the transcription factor Cap1 and the TOR signalling was also determined. Together, these findings expand our understanding of the complex signalling and transcriptional mechanisms regulating stress responses in *C. albicans*.

## Introduction

*Candida albicans* is a commensal fungus that can cause opportunistic infections, particularly in immunocompromised patients. Worst of all, *C. albicans* can even cause bloodstream infections, and the mortality rate of these infections can reach more than 50% [1]. An important feature related to *C. albicans* pathogenicity and virulence is its adaptability to harsh environmental conditions, including changes in pH and temperature, limitation of nutrients, and attack from the immune system [2–5]. Through different stress responsive mechanisms, *C. albicans* is able to overcome adversities, allowing the pathogen to grow and proliferate within the host [6,7]. These stress responses are coordinately regulated by various components of signalling and regulatory networks [7].

The maintenance of oxidation-reduction balance, known as redox homeostasis, is closely associated with generation and elimination of reactive oxygen species (ROS) that are by-products of aerobic metabolism [8,9]. Excessive ROS can induce oxidative stress and damage DNA, lipids, and proteins, leading to cell death. *C. albicans* encounters ROS-induced oxidative stress during the respiratory burst in macrophages and neutrophils, as well as from ROS-generating antifungal drugs such as amphotericin B, miconazole, and caspofungin [10–12]. To cope with oxidative stress in *C. albicans*, the involvement of antioxidants (superoxide dismutase, catalase, glutathione, and thioredoxin system), the Hog1 mitogen-activated protein kinase (MAPK) signalling, and the transcription regulator Cap1 are well recognized [9,10,13].

Repressor activator protein 1 (Rap1) is a multifunctional DNA-binding protein identified in different eukaryotic organisms [14]. In *Saccharomyces cerevisiae*, Rap1 plays an important role in subtelomeric gene silencing [15], telomere length control [16], and telomere end protection [17–19]. In addition, *S. cerevisiae* Rap1 also possesses other non-telomeric functions, including transcriptional regulation and metabolic control [20,21]. Similarly, *C. albicans* Rap1 participates in the control of telomere length and structure [22–24]. However, interestingly, we have previously found that *C. albicans* Rap1 is also associated with cell wall integrity and biofilm formation, and modulates phagocytosis and cytokine release by host immune cells [25]. Importantly, Rap1 is also related to *C. albicans* virulence as shown in a *Galleria mellanella* infection model.

In the present study, we aimed to explore more functions of Rap1 in *C. albicans*. Using RNA sequencing (RNA-seq) data with Gene Ontology (GO) analysis, different categories of the differentially expressed genes (DEGs) between the *RAP1*-deletion (*rap1*Δ/Δ) and wild-type strains were revealed, including the category of cell wall, membrane, and redox process. Here, we characterized the role of Rap1, particularly with respect to its function in oxidative stress response, which is associated with redox process. The *rap1*Δ/Δ mutant was much more resistant to the superoxide generator menadione, altered intracellular ROS levels, and had higher expression levels of superoxide dismutase genes. In addition, the Hog1 MAPK signaling and Cap1 were somehow associated with the Rap1-mediated response to menadione. Finally, using Gene Set Enrichment Analysis (GSEA) and other approaches, the link between the Target of Rapamycin (TOR) signalling pathway and Rap1-mediated response to menadione was also uncovered. Together, our findings not only reveal new functions of Rap1 but also highlight the complex signaling and regulation of oxidative stress response in *C. albicans*.

### Hypothesis

Rap1 is a DNA-binding protein identified in different eukaryotes including yeasts and mammals. Other than telomeric functions, nontelomeric activities have been revealed in *S. cerevisiae* and mammalian cells. Therefore, we hypothesized that *C. albicans* Rap1 may also possess nontelomeric functions. Indeed, our previous study linked *C. albicans* Rap1 to cell wall integrity, biofilm formation and interactions with host immune cells [25]. In the present study, we used genomic profiling and other approaches to further explore new functions of *C. albicans* Rap1 and found its role in oxidative stress response and a new relationship with the TOR signalling.

## Materials and methods

### *C. albicans* strains and growth conditions

*C. albicans* strains used throughout this study include SC5314 (*RAP1*/*RAP1*), *RAP1*-deletion (*rap1*Δ*/*Δ), and *RAP1*-reintegrated (*rap1*Δ::*RAP1/rap1*Δ::*RAP1*) strain [25]. All strains were routinely maintained at −80°C and plated on YPD agar (1% of yeast extract, 2% of peptone [Condalab, Madrid, Spain], and 2% of glucose). A single colony was inoculated in YPD broth and grown overnight at 30°C with shaking (180 rpm). The overnight grown cells were subcultured in synthetic complete (SC) medium (0.67% of yeast nitrogen base [YNB] with ammonium sulfate, 2% of glucose and 0.079% complete supplement mixture [MP Biomedicals, Santa Ana, CA, USA]) with an initial optical density at 600 nm (OD_600_) of 0.5 and grown at 30°C to the early exponential phase. All reagents were purchased from Sigma-Aldrich (St. Louis, MO, USA) unless indicated otherwise.

### Susceptibility to menadione and rapamycin

*C. albicans* cells were collected and resuspended in sterile phosphate-buffered saline (PBS). Ten microliters of 10-fold serial dilution of cells were spotted onto agar plates supplemented with menadione or rapamycin (Cayman Chemical, Ann Arbor, MI, USA) as indicated. Cell viability was recorded after incubation at 30°C for 3 days.

### RNA-Seq and transcriptome analysis

Total RNA was extracted using TRIzol Reagent (Invitrogen, Waltham, MA, USA), and ribosomal RNA (rRNA) was removed using the RiboMinus Eukaryote Kit (Thermo Fisher Scientific, Waltham, MA, USA). The yield and quality of purified RNA were assessed using an ND-1000 spectrophotometer (Nanodrop Technology, Wilmington, DE, USA) and a Bioanalyzer 2100 with the RNA 6000 Nano kit (Agilent Technology, Santa Clara, CA, USA). The RNA library was prepared using the SureSelect XT HS2 mRNA Library Preparation kit (Agilent), followed by DNA cleanup and size selection using AMPure XP beads (Beckman Coulter, Brea, CA, USA). Sequencing data (FASTQ reads) were generated using the Illumina Solexa platform (Illumina, San Diego, CA, USA) based on Illumina's basecalling program bcl2fastq v2.20. Sequencing reads were aligned to the *C. albicans* SC5314 genome assembly 22 (http://www.candidagenome.org/) with the HISAT2 alignment program to generate read counts [26]. RNA-seq data are available in the NCBI Sequence Read Archive (SRA) with the accession number PRJNA1114919. Three independent biological replicates of *C. albicans* cultures grown in SC medium to the early exponential phase were used for transcriptome analysis.

Transcriptome analysis was performed using raw read counts, and normalization along with differential expression analysis was carried out using the Bioconductor DESeq2 package (version 3.18) within the RStudio environment (version 4.3) under default parameters. DESeq2 models gene counts with a generalized linear model (GLM) that uses a negative binomial distribution to account for biological variability. The Wald test was used for hypothesis testing between two conditions [27]. Dispersion estimation was performed using parametric fitting (fitType), modeling dispersion as a function of the mean, with empirical Bayes shrinkage applied for stabilization. Additionally, multiple testing was corrected using the Benjamini-Hochberg method to control the false discovery rate (FDR) and to generate adjusted *p*-values shown in Supplementary Table S1. Gene Ontology enrichment analysis was subsequently conducted using the FungiFun2 tool (version 2.2.8 BETA) to identify enriched biological processes, cellular components, and molecular functions associated with the DEGs [28].

For GSEA, the pre-rank analysis was conducted using the Gseapy package (version 1.0.6), a Python wrapper for GSEA. Pre-ranked correlation table was generated from the differential expression results from DESeq2, ranked by log2 fold change. The gene set collection for running GSEA was obtained from Andre Nantel (http://www.candidagenome.org/download/community/GSEA_Nantel_2012/). Default parameters were applied, except for the maximum and minimum gene set sizes, which were adjusted to 250 and 15, respectively. To enhance the robustness of the analysis, the number of permutations was increased to 30,000. The pre-ranked results were sorted by FDR. For the results with an FDR of 0, the value was set to less than 1 divided by the number of permutations. The results were output as a table for further analysis.

For creating heatmaps, the normalization results from the differential expression analysis were converted into z-scores using the ‘zscore’ function from the ‘scipy.stats' module (SciPy version 1.11.2) along the gene axis. The heatmap was then generated using the ’clustermap’ function from the Seaborn library (Seaborn version 0.12.2). All analyses were performed using Python 3.11.5 in the Visual Studio Code environment.

### Real-time quantitative PCR (qPCR)

RNA isolation and reverse transcription for cDNA synthesis were performed as previously described [29]. Real-time qPCR was conducted using the ABI 7500 Fast Real-Time PCR System (Applied Biosystems, Waltham, MA, USA). The primers used for real-time qPCR are listed in Supplementary Table S2. Gene expression levels were normalized to the *ACT1* transcript levels [30]. All experiments were performed in triplicate, with three independent experiments for each strain, and the average cycle threshold (C_T_) values were determined. The 2^−ΔΔCT^ method was used to calculate the relative fold change in the expression of each gene [31].

### Measurement of intracellular ROS accumulation

Cells were treated with or without menadione (300 μM) for 1 hour and washed twice with sterile PBS. To measure superoxide anion accumulation, cells were stained with dihydroethidium (DHE, 20 μM, Thermo Fisher Scientific) for 10 min. After staining, cells were washed twice with PBS, and the mean fluorescence intensity was measured with PE detector using a CytoFLEX flow cytometer (Beckman Coulter, Brea, CA, USA).

### Protein extraction and western blot

Cells were harvested and resuspended in Radio-Immunoprecipitation Assay (RIPA) lysis buffer containing various protease inhibitors [25]. Cells were broken by vortexing with acid-washed glass beads. Supernatants were collected by centrifugation, and total proteins were extracted [25]. The total protein concentration was determined using a Bio-Rad Bradford assay (Bio-Rad, Hercules, CA, USA).

For western blotting to detect phosphorylated Hog1 (Hog1-p) and Rps6 (Rps6-p), proteins were separated by 10% of sodium dodecyl sulfate (SDS)-polyacrylamide gel electrophoresis as previously described [32]. Hog1-p and total Hog1 were detected using anti-phospho-p38 MAPK (catalog no. 9215; Cell Signalling Technology, Danvers, MA, USA) and anti-Hog1 antibodies (catalog no. sc-9079; Santa Cruz Biotechnology, Santa Cruz, CA, USA), respectively. Moreover, anti-phospho-(Ser/Thr) Akt substrate antibody (catalogue no. 9611; Cell Signalling Technology) was used to detect Rps6-p, and anti-ribosomal protein S6/RPS6 antibody (catalogue no. AF5436; R&D Systems, Minneapolis, MN, USA) was used to detect total Rps6. Horseradish peroxidase (HRP)-conjugated anti-rabbit IgG (no. GTX213110-01, GeneTex, Hsinchu, Taiwan), and HRP-conjugated anti-sheep IgG (catalogue no. HAF016, R&D Systems) were used as the secondary antibodies. HRP was detected by the Western Lightning Plus chemiluminescent substrates (PerkinElmer, Waltham, MA, USA), and the blot images were taken using an ImageQuant LAS 4000 biomolecular imager (GE Healthcare, Chicago, IL, USA).

## Results

### Transcript profiling reveals expression differences in the *rap1*Δ/Δ and wild-type strains

**Figure 1:**
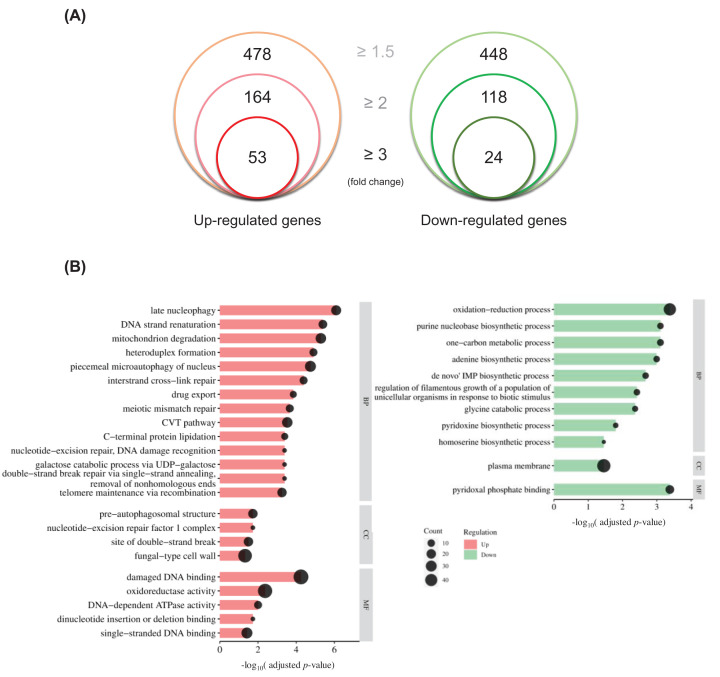
Gene expression comparison using RNA-seq (**A**) Differentially expressed genes (DEGs) in the *rap1*Δ/Δ mutant compared with the wild-type strain. Numbers of the DEGs with an adjusted *P*<0.05 and fold-change more than and equal to 1.5-, 2- and 3-fold were shown. (**B**) Gene ontology (GO) analysis of the DEGs. The most significantly (adjusted *P*<0.05) enriched GO term in biological process, molecular function, and cellular component branches are presented. Gene numbers in each GO term are indicated. The entire result of this GO analysis is listed in Supplementary Table S3. BP, Biological process; CC, Cellular component; MF, Molecular Function.

As mentioned above, Rap1 is involved in cell wall integrity, biofilm formation, and virulence in *C. albicans* [25]. To explore other functions of Rap1 in this important pathogen, RNA-seq was used to profile gene expression in the *rap1*Δ/Δ and wild-type strains. A total of 926 genes were significantly modulated (≥ 1.5-fold change and adjusted *P*<0.05, see Supplementary Table S2). Among them, 478 genes were up-regulated, and 448 genes were down-regulated in the *rap1*Δ/Δ compared with the wild-type strain (Figure 1A).

GO analysis was then performed to identify enriched biological categories for the DEGs using the FungiFun2 functional enrichment tool [28]. A total of 23 and 11 GO terms were independently identified in the up-regulated and down-regulated genes of the *rap1*Δ/Δ mutant compared with the wild-type strain. These categories involve various biological processes, molecular functions, and cellular components (Figure 1B and Supplementary Table S3). Among the up-regulated genes, the GO terms include “telomere maintenance via recombination,” “interstrand cross-link repair,” “damaged DNA binding,” and “fungal-type cell wall”. The identification of these categories is consistent with previous findings that Rap1 plays a role in telomere regulation and cell wall integrity [24,25,33]. Moreover, the GO terms of the down-regulated genes include metabolic processes, such as “purine nucleobase biosynthetic process”, and “de novo IMP biosynthetic process”. Interestingly, many up-regulated and down-regulated genes were related to “oxidoreductase activity” and “oxidation-reduction process”. However, the relationship between Rap1 and redox-related events has yet to be studied.

### Rap1 is involved in oxidative stress response in *C. albicans*

Redox processes are primarily linked to aerobic metabolism for energy generation and are associated with the production of ROS [8,9]. ROS not only modulate redox reactions, but also influence intracellular redox homeostasis [34]. Moreover, ROS can cause oxidative damage, and the response to ROS-mediated oxidative stress is critical for *C. albicans* pathogenicity and virulence [10]. For example, massive ROS generation by host immune cells of the host can cause irreversible damage and death of *C. albicans* cells [10]. As shown in Figure 1B and Supplementary Table S3, GO enrichment analysis suggested an association between Rap1 and redox process. Therefore, we aimed to investigate potential differences in oxidative stress response among the wild-type, *rap1*Δ/Δ, and *RAP1*-reintegrated strains.

**Figure 2:**
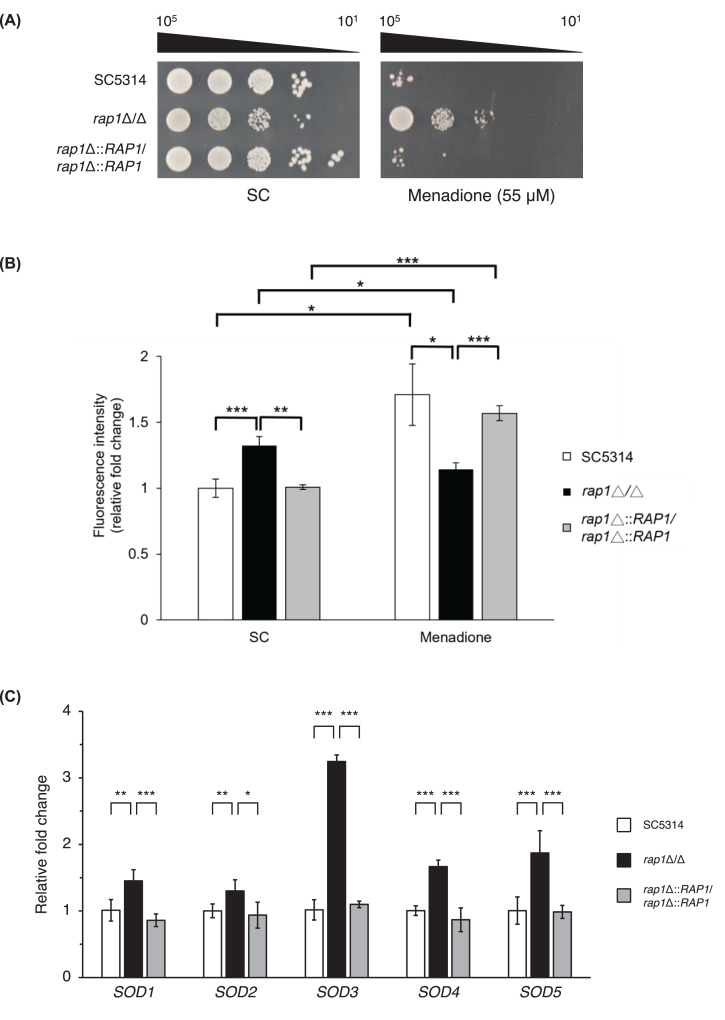
Menadione susceptibility assay and superoxide dismutase (*SOD*) gene expression (**A**) Cellular susceptibility to menadione. Cells were ten-fold serially diluted and spotted onto SC agar plates with or without 55 μM menadione. Cells were incubated at 30°C for 3 days. Representative images of three independent experiments with identical results are shown. (**B**) Measurement of the superoxide content measurement using DHE staining. Cells were stained with DHE (20 μM) and the mean fluorescence intensity (MFI) of 20,000 cells was determined by flow cytometry. The results are expressed as the mean ± standard deviation (SD) of three independent experiments. **, *P*<0.01; ***, *P*<0.001. (**C**) Assessment of *SOD* gene expression. Cells were treated with 300 μM menadione and incubated at 30°C for 1 h, and the expression of *SOD* genes was analyzed using real-time qPCR. The *ACT1* transcript was used as an endogenous control. The results are displayed as the mean ± SD from three independent experiments. *, *P*<0.05; **, *P*<0.01; ***, *P*<0.001.

To test this hypothesis, we examined cell susceptibility to menadione using a spot assay. Figure 2A shows that the *rap1*Δ/Δ mutant exhibited significantly higher resistance to the superoxide generator menadione compared with the wild-type and *RAP1*-reintegrated cells. Superoxide anions represent the main resource of ROS in cells and are produced as by-products of the mitochondrial electron transport chain (ETC). In addition, superoxide levels were measured using DHE staining and analyzed by flow cytometry. The result indicated that intracellular superoxide levels increased in wild-type and *RAP1*-reintegrated cells treated with menadione compared with untreated cells. However, in the *rap1*Δ/Δ mutant, intracellular superoxide levels decreased slightly after menadione treatment compared with untreated mutant cells. Furthermore, menadione-treated *rap1*Δ/Δ mutant cells exhibited lower intracellular superoxide levels than wild-type and *RAP1*-reintegrated strains (Figure 2B).

ROS levels and cellular redox state are regulated by various antioxidant enzymes [35,36], which are closely associated with cellular responses to oxidative stress [10]. In *C. albicans*, superoxide dismutases (Sods) play a critical role by converting superoxide into hydrogen peroxide (H_2_O_2_) [10,37]. To further explore the function of Rap1 in oxidative stress response, the expression of *SOD* genes was monitored using real-time qPCR. The results revealed that the expression of *SOD* genes was upregulated in the *rap1*Δ/Δ mutant upon menadione treatment compared with the control strains (Figure 2C). Together, these results suggest a link between Rap1 and oxidative stress response in *C. albicans*.

### The Hog1 signalling pathway and the transcription factor Cap1 associated with Rap1-mediated oxidative stress response

**Figure 3:**
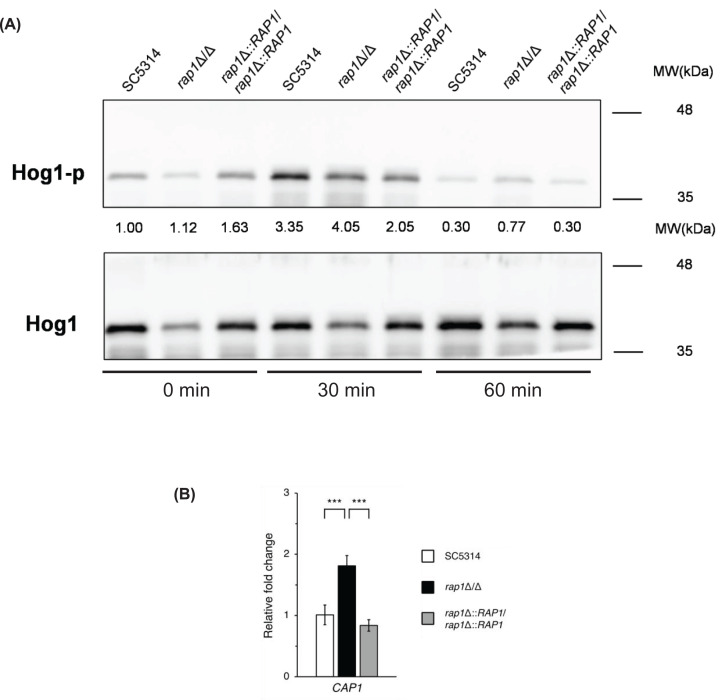
The Hog1 signaling and the transcription factor Cap1 are associated with Rap1-mediated oxidative stress response (**A**) Activation of the Hog1 MAPK. Cells were treated with 300 μM menadione for 0, 30, and 60 min. Then, Hog1 phosphorylation was detected by Western blotting and analyzed by ImageJ software. The total Hog1 band of each sample was served as the loading control to normalize the Hog1-p levels and the fold-change values were indicated. The data are representative of three independent experiments with identical results. (**B**) The expression of the *CAP1* gene. Cells were treated with 300 μM menadione for 1 h, and the gene expression level of *CAP1* was analyzed by real-time qPCR. The *ACT1* transcript was used as an endogenous control. The results are displayed as the mean ± SD from three independent experiments. ***, *P*<0.001.

In *C. albicans*, the Hog1 MAPK pathway is known to function in the regulation of oxidative stress response [10,38,39]. Moreover, independent of the Hog1 pathway, Cap1 is a major regulator in the transcriptional responses to oxidative stress [10,39–41]. Based on the results that correlate Rap1 to oxidative stress response (Figure 2A–C), we were therefore interested in examining the relationship among Hog1, Cap1, and Rap1. As shown by semi-quantitative analysis of western blots, the *rap1*Δ/Δ mutant had an overall higher level of phosphorylated Hog1 (Hog1-p) than the wild-type and *RAP1*-reintegreated strains, particularly in cells treated with menadione for 30 min (Figure 3A). Interestingly, the expression of the *CAP1* gene was upregulated in the *rap1*Δ/Δ mutant compared with the other two control strains (Figure 3B). These results suggest that the Hog1 pathway and Cap1 may also be involved in the Rap1-mediated oxidative stress response.

### The TOR signalling pathway is also related to Rap-mediated oxidative stress response

In response to nutrient availability and stress conditions, the yeast TOR signalling pathway regulates various cellular processes, including protein synthesis, ribosome biogenesis, and autophagy. Thereby, the TOR pathway is critical in coordination of cell growth, proliferation, metabolism, and cell cycle progression [42,43].

**Figure 4:**
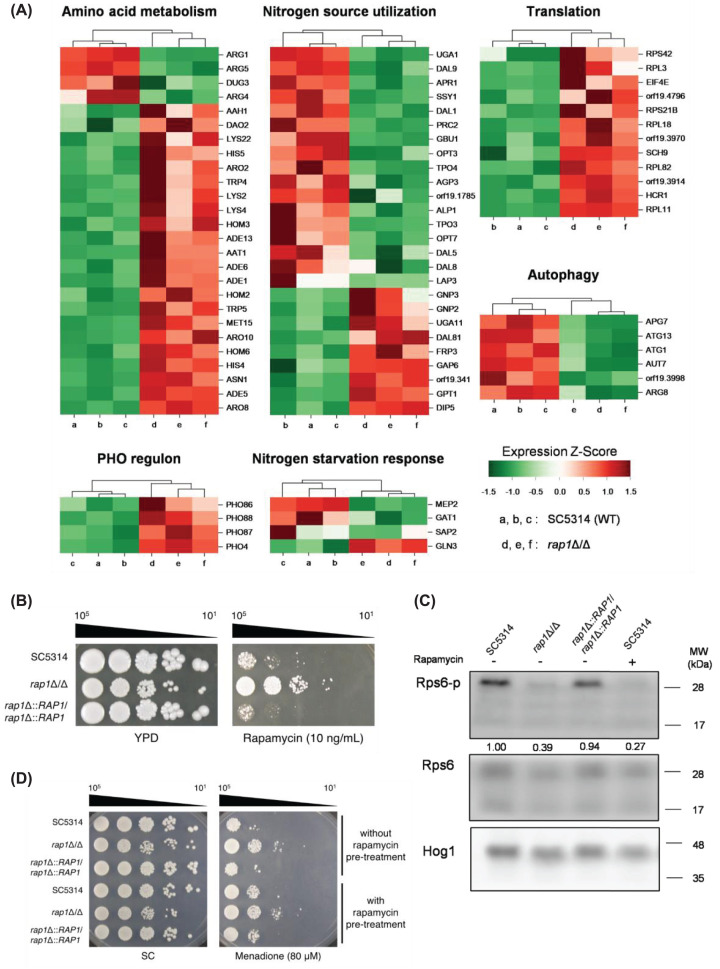
The association of the TOR signaling with Rap1-mediated oxidative stress response (**A**) Heat map of differentially expressed genes (DEGs) probably related to the TOR pathway. The DEGs were sorted based on their functions and analyzed by hierarchical clustering calculation with their expression fold changes from RNA-seq analysis to generate Z-score. The Z-score of each data was then used to generate the heat map. a, b, c: the wild-type strain; d, e, f: the *rap1*Δ/Δ mutant strain. (**B**) Cellular susceptibility to rapamycin. Cells were ten-fold serially diluted and spotted onto YPD agar plates with or without rapamycin. Cells were cultivated at 30°C for 3 days. Representative images of three independent experiments with identical results are shown. (**C**) Phosphorylation of the ribosomal protein Rps6 was assayed by Western blotting. Rps6 and Hog1 protein was used as the loading controls, and the phosphorylation ratio (Rps6-p/Rps6) was analyzed by using ImageJ software. The data are representative of three independent experiments with identical results. (**D**) Cellular susceptibility to menadione with or without blocking the TOR signalling. Cells were pre-treated with or without rapamycin (200 ng/mL) for 1 h and spotted onto SC plates with or without menadione. Viability was recorded after cell growth at 30°C for 3 days. Representative images of three independent experiments with identical results are shown.

To derive more insights into Rap1 functions from our RNA-seq data, GSEA was further performed, using an extensive gene data set generated by Nantel and colleagues [44]. Using a false discovery rate (FDR) ≤ 0.25 as a cut-off, 227 gene sets were enriched (Supplementary Table S4). Interestingly, the enriched groups include ‘ribosome biogenesis & assembly,’ ‘translation,’ and ‘ribosome’, which are all related to protein biosynthesis. More intriguingly, several groups of our DEGs were also found to be related to translation, nitrogen starvation response, amino acid metabolism, autophagy, and PHO regulon (Supplementary Table S2 and Figure 4A). Together, we suspected that the TOR signalling may also be involved in Rap1 functions.

To examine the possible link between the TOR pathway and Rap1, spot assay of viability of cells treated with or without rapamycin was conducted. Rapamycin is an inhibitor of the Tor1 kinase, which is the key component of the TOR pathway. The *rap1*Δ/Δ mutant had much higher resistance to rapamycin than the wild-type and *RAP1*-reintegrated strains (Figure 4B). In addition, the ribosomal protein S6 (Rps6) is a downstream target of the TOR signalling and is phosphorylated through the TOR activity in *C. albicans* [45]. To further connect the TOR pathway and Rap1, levels of phosphorylated Rps6 (Rps6-p) were measured by western blotting. In Figure 4C, as a control, cells treated with rapamycin had no detectable Rps6-p. However, without rapamycin treatment, the levels of Rps6-p were increased in the wild-type and *RAP1*-reintegrated cells, but not in the *rap1*Δ/Δ mutant. Finally, the relationship between the TOR pathway and Rap1-mediated oxidative stress response was tested. Cells were pre-treated with or without rapamycin for 1 h, followed by spotted onto SC agar plates with or without menadione. As shown in Figure 4D, the wild-type and *RAP1*-reintegrated strains were more resistant to menadione in cells pretreated with rapamycin than those without rapamycin pretreatment. However, the *rap1*Δ/Δ mutant was not affected by rapamycin and exhibited a much more resistance to menadione. These results suggest the role of Rap1 in oxidative stress response modulated by the TOR signalling.

## Discussion

Rap1 is a multifunctional DNA binding protein identified in various eukaryotic organisms, including mammals, *S. cerevisiae*, and *C. albicans* [20]. In *S. cerevisiae* and *C. albicans*, Rap1 shares similar functions in telomere maintenance [20]. However, divergent structures and functions exist between Rap1 in *S. cerevisiae* and *C. albicans*. First, *C. albicans* Rap1 exhibits an unusual truncated protein structure lacking the C-terminal domain, which is implicated in telomere regulation of *S. cerevisiae* Rap1 [24]. Secondly, *S. cerevisiae RAP1* is an essential gene, whereas *RAP1* is not essential for cell viability of *C. albicans* [24]. Intriguingly, our recent study demonstrated that *C. albicans* Rap1 is involved in cell wall integrity, biofilm formation, and virulence [25], functions not displayed in *S. cerevisiae* Rap1. Therefore, expanding our knowledge of new functions of Rap1 in *C. albicans* is of great interest.

To explore this further, RNA-seq analysis was performed. Using GO analysis on RNA-seq data measuring differential expression between the *rap1*Δ/Δ and wild-type strain, many GO terms including redox process were enriched (Figure 1B and Supplementary Table S3). By measuring cell susceptibility to menadione, intracellular ROS accumulation, and the expression levels of the superoxide dismutase *SOD* genes (Figure 2A–C), we further demonstrated that Rap1 is involved in the oxidative stress response, which is associated with redox process.

Both menadione and H_2_O_2_ are commonly used oxidants in studies of cellular oxidative stress response [46]. As shown in Figure 2A, the *rap1*Δ/Δ mutant exhibited much greater resistance to menadione compared with the wild-type and *RAP1*-reintegrated strains. Interestingly, however, we observed no significant differences in susceptibility to H_2_O_2_ among all the tested strains (data not shown). It is well known that menadione and H_2_O_2_ function differently and elicit distinct cellular responses in both yeast and mammalian cells [46–48]. Menadione primarily triggers an integrated stress response, whereas H_2_O_2_ broadly influences biological processes [46]. Although the observation that *RAP1* deletion leads to different cellular responses to menadione and H_2_O_2_ is intriguing, further studies are needed to elucidate the detail mechanisms. Additionally, while all *SOD* genes were upregulated in the *rap1*Δ/Δ mutant, the upregulation of *SOD3* was more pronounced (Figure 2C). The *SOD3* gene encodes a cytoplasmic manganese-containing superoxide dismutase in *C. albicans*, whose activation is regulated by the copper-responsive transcription factor Mac1 during copper limitation in the stationary phase [49–51]. Notably, overexpression of *SOD3* alleviate menadione toxicity and rescues cell growth [49], suggesting that the reduced ROS accumulation and enhanced resistance to menadione observed in *rap1*Δ/Δ mutant may be partially attributed to the elevated expressed *SOD3* (Figure 2A–C). Investigating how Rap1 regulates *SOD3* expression and the potential connection between Rap1, copper and Mac1 will be valuable for further research.

Rap1 has been implicated in oxidative stress response in mammalian cells through the NF-κB signalling pathway and sirtuins [20]. Moreover, Rap1 levels are affected by oxidative stress, and Rap1-deficient mice exhibit elevated oxidative stress [52,53]. Also, in response to mild ethanol stress, increased expression of Rap1 and sirtuin proteins (Sir1, Sir2, and Sir3) is accompanied by the increase in total ROS and superoxide in industrial wine yeast strains [54]. Sirtuins are a class of nicotinamide adenine dinucleotide (NAD^+^)-dependent deacetylases, involved in signal transduction and various biological processes [55]. Interestingly, the association between telomeres and sirtuins has been a focus of some research [56,57], and several subtelomeric genes were identified as differentially expressed in the *rap1*Δ/Δ mutant in this study (Supplementary Tables S1 and S5). Therefore, whether telomeres and Sir proteins affect Rap1-mediated oxidative stress response is worth further investigation.

*C. albicans* encounters oxidative stress from host phagocytes and antifungals [10,11]. The well-known mechanism to respond to oxidative stress and activate antioxidant gene expression is primarily mediated by the Hog1 MAPK signalling and the transcription factor Cap1 [58,59]. In response to oxidative stress, *C. albicans* Hog1 is rapidly activated and accumulates in the nucleus, though it does not play a major role in regulating oxidative stress-related gene expression [60,61]. Additionally, Cap1 is normally oxidized in response to H_2_O_2_, leading to its nuclear accumulation and activation of target genes [59,62]. Although our results showed that *RAP1* deletion affects Hog1 phosphorylation and *CAP1* expression level, the detailed mechanisms still remain to be elucidated.

Recently, TOR signalling has been linked to oxidative stress in *C. albicans*. Lack of the N-terminal HEAT repeats in the Tor1 kinase led to significantly impaired cellular response to oxidative stress [63]. Furthermore, overexpression of Gtr1, the activating GTPase of the TOR complex 1, rescues the Sod3 deficiency in the manganese transporter *PHO84*-deletion mutant [63]. Interestingly, our previous studies found that the transcription factor Sfp1, which functions downstream of the Tor1 signalling, also regulates oxidative stress response in *C. albicans* [64,65]. In the present study, we further established a correlation between Rap1-mediated oxidative stress response and TOR signalling (Figure 4B–D). Future studies will be needed to examine the relationship between Rap1, TOR signalling, Sod3, Pho84, and Sfp1.

In conclusion, our findings not only reveal new functions of Rap1 but also underscore the complex regulatory machinery involved in the oxidative stress response of *C. albicans*.

### Future Directions

Based on our findings in the present study, at least two main directions are worth considering for future research. First, whether and how telomeres and Sir proteins are associated with Rap1-mediated oxidative stress response in *C. albicans*. Secondly, how Rap1 interplays with different signalling pathways and other transcription regulators in controlling oxidative stress response in *C. albicans*.

## Supplementary Material

Supplementary Tables S1-S5 and Supplementary Material

## Data Availability

The data presented in this study are available within the article and the Supplementary material. RNA-seq data are available in the NCBI Sequence Read Archive (SRA) with the accession number PRJNA1114919.

## Abbreviations

DEGs: differentially expressed genes
DHE: dihydroethidium
FDR: false discovery rate
GSEA: gene set enrichment analysis
MAPK: mitogen-activated protein kinase
PBS: phosphate-buffered saline
qPCR: quantitative PCR
RAP1: repressor activator protein 1
ROS: reactive oxygen species
SD: standard deviation
SOD: superoxide dismutase
