# Supplementary material for: Transcriptional profiling reveals the role of Candida albicans Rap1 in oxidative stress response
Source: Biosci Rep. 2024 Dec 12;44(12):BSR20240689. doi: 10.1042/BSR20240689 (PMC11667096; doi:10.1042/BSR20240689)

Supplementary file

Supplementary Table S1. List of differentially expressed genes (DEGs)

Table S1 is provided in a separate file. The detailed information is described in Materials and Methods of the text.

Supplementary Table S2. Primers used in this study.

Primer name	Sequence (5' to 3')
For real-time qPCR	
ACT1-F	ATACTCTGTCTGGATTGGTGGTTCT
ACT1-R	TTTTGAAATCCACATTTGTTGGA
SOD1-F	TCCGAATCCGCTCCAACCACA
SOD1-R	AAATGAGGACCAGCAGAAGTACAACCA
SOD2-F	TCA ATT GAA CAA GCC GTT GAA GCC AAA
SOD2-R	ACCACCTTGAGAGACAGGAGCCA
SOD3-F	CAATGCCGCTATTGACGCACTTGA
SOD3-R	TCCAGAACAAACTGTGGTTGGTGTGT
SOD4-F	TTTGAGCCAGCAAACAATGG
SOD4-R	CACCTGAAGGCAATCCAGTTAAA
SOD5-F	AAGGATTGCCCTCTGATATTGG
SOD5-R	GATGCTGGCACTGGTTTTTCA
CAP1-F	TGACAACTTTGATCTTGCATTGG
CAP1-R	TGGATCGGCTTCTGCTTCAT

Supplementary Table S3. Result of Gene ontology (GO) analysis

Up-regulated genes			
GO ID	GO name	Adjusted *p*-value (padj)	Numbers in the group
Biological Process			
GO:0034727	piecemeal microautophagy of nucleus	0.002573565	10/28
GO:0032258	CVT pathway	0.01855553	9/31
GO:0000422	mitochondrion degradation	0.001024215	9/20
GO:0044805	late nucleophagy	0.000269434	8/13
GO:0000722	telomere maintenance via recombination	0.02413165	7/21
GO:0000733	DNA strand renaturation	0.00100429	6/8
GO:0030491	heteroduplex formation	0.002085161	5/6
GO:0036297	interstrand cross-link repair	0.004567702	5/7
GO:0000710	meiotic mismatch repair	0.01672866	5/9
GO:0006501	C-terminal protein lipidation	0.01855553	4/6
GO:0046618	drug export	0.01181907	4/5
GO:0000736	double-strand break repair via single-strand annealing, removal of nonhomologous ends	0.01855553	3/3
GO:0033499	galactose catabolic process via UDP-galactose	0.01855553	3/3
GO:0000715	nucleotide-excision repair, DNA damage recognition	0.01855553	3/3
Molecular function			
GO:0003684	damaged DNA binding	5.64476E-05	11/22
GO:0032139	dinucleotide insertion or deletion binding	0.01855553	3/3
GO:0008094	DNA-dependent ATPase activity	0.009937789	5/8
GO:0016491	oxidoreductase activity	0.004274802	19/92
GO:0003697	single-stranded DNA binding	0.03822519	10/43
Cellular component			
GO:0009277	fungal-type cell wall	0.04767009	17/102
GO:0000110	nucleotide-excision repair factor 1 complex	0.01855553	3/3
GO:0000407	hyphal cell wall	0.01855553	7/20

GO:0035861	site of double-strand break	0.031776	7/22
Down-regulated genes			
GO ID	GO name	Adjusted *p*-value (padj)	Numbers in the group
Biological Process			
GO:0009113	purine nucleobase biosynthetic process	0.000416659	6/7
GO:0046084	adenine biosynthetic process	0.000784346	6/8
GO:0006189	de novo' IMP biosynthetic process	0.000784346	6/8
GO:0006730	one-carbon metabolic process	0.001010269	7/12
GO:0055114	oxidation-reduction process	0.002120296	38/264
GO:0008615	pyridoxine biosynthetic process	0.003810314	4/4
GO:0006546	glycine catabolic process	0.004289571	5/7
GO:1900443	regulation of filamentous growth of a population of unicellular organisms in response to biotic stimulus	0.01579998	6/13
GO:0009090	homoserine biosynthetic process	0.03509764	3/3
Molecular function			
GO:0030170	pyridoxal phosphate binding	0.000416659	14/42
Cellular component			
GO:0005886	plasma membrane	0.03509764	47/408

Supplementary Table S4. Result of Gene Set Enrichment Analysis (GSEA)

Table S4 is provided in a separate file. The detailed information is described in Materials and Methods of the text.

Supplementary Table S5. DEGs in the *rap1*Δ/Δ mutant that are located in the subtelomeric region

Table S5 is provided in a separate file. The detailed information is described in Materials and Methods of the text.

Supplementary Table S1: List of Differentially Expressed Genes (*rap1*Δ/Δ versus wild type)

Gene ID	Orf identifier	Gene name	Fold change[a]	log2 Fold change	Adjusted *p*-value (padj)	Description[b]
CR_08510W_A	orf19.6420	PGA13	60.37004409	5.91576095	1.2788E-131	GPI-anchored cell wall protein involved in cell wall synthesis; required for normal cell surface properties; induced in oralpharyngeal candidasis; Spider biofilm induced; Bcr1-repressed in RPMI a/a biofilms
C1_13430C_A	orf19.4970	orf19.4970	54.11826475	5.758043676	5.1592E-227	Protein of unknown function; Spider biofilm induced
C1_04140W_A	orf19.1048	IFD6	19.82198158	4.309029289	2.05922E-91	Aldo-keto reductase; similar to aryl alcohol dehydrogenases; protein increase correlates with MDR1 overexpression (not CDR1 or CDR2) in fluconazole-resistant clinical isolates; farnesol regulated; possibly essential; Spider biofilm induced
CR_02920C_A	orf19.2849	AQY1	13.9370759	3.800856	2.7658E-56	Aquaporin water channel; osmotic shock resistance, WT freeze tolerance; virulent in mice; flucytosine repressed; flow model/RPMI/Spider/rat catheter biofilm induced; required for RPMI biofilm formation; Bcr1-induced in a/a RPMI biofilms
C7_00630C_A	orf19.7056	orf19.7056	13.21640392	3.724257779	2.7641E-172	Putative protein of unknown function; transcript is upregulated in clinical isolates from HIV+ patients with oral candidiasis; regulated by Sef1, Sfu1, and Hap43
CR_04990C_A	orf19.654	CIS305	9.81752957	3.295360038	1.04557E-29	Predicted protein only found in *Candida albicans* and *Candida dubliniensis*; highly induced during chlamydospore development
C1_04010C_A	orf19.4476	orf19.4476	8.817121267	3.140307702	5.2927E-172	Protein with a NADP-dependent oxidoreductase domain; transcript induced by ketoconazole; rat catheter and Spider biofilm induced

C3_01540W_A	orf19.1691	orf19.1691	7.858788671	2.974306957	0.005074112	Plasma-membrane-localized protein; filament induced; Hog1, ketoconazole, fluconazole and hypoxia-induced; regulated by Nrg1, Tup1, Upc2; induced by prostaglandins; flow model biofilm induced; rat catheter and Spider biofilm repressed
C5_05510C_A	orf19.4055	orf19.4055	6.571298533	2.716178485	5.42678E-10	Protein similar to *Saccharomyces cerevisiae* Ybr075wp; transposon mutation affects filamentous growth; clade-associated gene expression
C2_04010C_A	orf19.822	HSP21	6.555831476	2.712778769	4.01176E-17	Small heat shock protein; role in stress response and virulence; fluconazole-downregulated; induced in cyr1 or ras1 mutant; stationary phase enriched protein; detected in some, not all, biofilm extracts; Spider biofilm induced
C3_00050C_A	orf19.5468	orf19.5468	6.243426275	2.64233797	2.00038E-20	Predicted ORF from Assembly 19; removed from Assembly 20; restored based on transcription data; almost identical to orf19.6113, orf19.4055, orf19.5370
C1_08790W_A	orf19.4737	TPO3	5.924533581	2.56670158	2.19367E-49	Putative polyamine transporter; MFS-MDR family; induced by Sfu1, regulated upon white-opaque; decreased expression in hyphae vs yeast-form cells; regulated by Nrg1; Spider biofilm repressed
C1_02990C_A	orf19.2990	XOG1	5.851809073	2.548882699	1.65039E-60	Exo-1, 3-beta-glucanase; 5 glycosyl hydrolase family member; affects sensitivity to chitin and glucan synthesis inhibitors; not required for yeast-to-hypha transition or for virulence in mice; Hap43-induced; Spider biofilm induced
C4_01340W_A	orf19.4653	orf19.4653	5.779268853	2.530886986	1.25074E-09	Protein similar to GPI-linked cell-wall proteins; induced in low iron; Spider biofilm induced; regulated in Spider biofilms by Bcr1, Tec1, Ndt80, Brg1
C2_01450C_A	orf19.1449	orf19.1449	5.646917493	2.497463552	1.18242E-41	Protein of unknown function; induced in azole-resistant strain that overexpresses MDR1; protein present in

						exponential and stationary growth phase yeast cultures; Spider biofilm induced
C4_03430W_A	orf19.3369	MOH1	5.628769275	2.492819513	1.18604E-17	Ortholog of *S. cerevisiae* Moh1, essential for staionary phase growth; induced by alpha pheromone in SpiderM medium and by Mnl1 under weak acid stress; possibly essential (UAU1 method); flow model biofilm induced; Spider biofilm induced
C5_04250W_A	orf19.3908	MRV8	5.152478916	2.365266696	1.21166E-21	Membrane protein involved in mycelial growth, biofilm formation and epithelial damage; Spider biofilm induced
C1_13160W_A	orf19.4943	PSA2	5.003889584	2.323049955	9.81883E-34	Mannose-1-phosphate guanyltransferase; Hap43, macrophage-repressed; stationary phase enriched protein; Spider biofilm induced; rat catheter biofilm repressed
C3_05620W_A	orf19.6997	ATO5	4.950914365	2.307694996	1.1575E-12	Putative fungal-specific transmembrane protein
C3_02330C_A	orf19.1611	orf19.1611	4.890253933	2.289909381	3.9905E-19	Protein of unknown function; Spider biofilm induced
C3_01130C_A	orf19.2515	orf19.2515	4.666815765	2.222438514	8.02839E-70	ZZ-type zinc finger protein; rat catheter and Spider biofilm induced
C1_10060C_A	orf19.4873	orf19.4873	4.65716725	2.219452694	1.22806E-14	Protein of unknown function; transcript regulated by white-opaque switch; flow model biofilm induced; Spider biofilm induced
C6_04380W_A	orf19.1097	ALS2	4.506860786	2.172122887	3.86034E-09	ALS family protein; role in adhesion, biofilm formation, germ tube induction; expressed at infection of human buccal epithelial cells; putative GPI-anchor; induced by ketoconazole, low iron and at cell wall regeneration; regulated by Sfu1p
C6_02950C_A	orf19.5573	orf19.5573	4.342359646	2.118479219	3.32171E-08	Protein of unknown function; expression downregulated in an ssr1 null mutant
C7_02260W_A	orf19.6487	orf19.6487	4.185507129	2.065402435	2.73284E-08	Ortholog of *Candida parapsilosis* CDC317: CPAR2_808350, *C. dubliniensis* CD36: Cd36_72060,

						Candida metapsilosis: CMET_5893 and *Candida orthopsilosis* Co 90-125: CORT_0C00820
CR_00620C_A	orf19.7469	ARG1	4.09105806	2.032474012	4.05548E-53	Argininosuccinate synthase; arginine synthesis; Gcn4, Rim101 regulated; induced by amino acid starvation (3-AT), benomyl treatment; stationary phase enriched protein; repressed in alkalinizing medium; rat catheter, Spider biofilm induced
C1_04020C_A	orf19.4477	CSH1	3.852804714	1.945909062	7.49239E-59	Aldo-keto reductase; role in fibronectin adhesion, cell surface hydrophobicity; regulated by temperature, growth phase, benomyl, macrophage interaction; azole resistance associated; Spider biofilm induced; rat catheter biofilm repressed
C1_09310C_A	orf19.4791	orf19.4791	3.774312531	1.91621389	5.05992E-39	Protein of unknown function; Spider biofilm induced
C2_03110W_A	orf19.5785	orf19.5785	3.688410808	1.882999349	2.00038E-20	Protein of unknown function; upregulated in a cyr1 or ras1 null mutant; induced by nitric oxide
C3_02060W_A	orf19.1641	orf19.1641	3.673010096	1.876962862	8.74554E-17	Ortholog(s) have extracellular region localization
CR_07220C_A	orf19.6148	orf19.6148	3.663336708	1.87315831	3.66822E-47	Homolog of nuclear distribution factor NudE, NUDEL; regulates dynein targeting to microtubule plus ends; flow model biofilm repressed
C1_07330W_A	orf19.4438	RME1	3.659981031	1.871836171	2.3511E-17	Zinc finger protein, controls asexual sporulation; white-specific transcript; upregulation correlates with clinical development of fluconazole resistance; Upc2-regulated in hypoxia; flow model biofilm induced; Spider biofilm
C1_10370W_A	orf19.4908	orf19.4908	3.614459479	1.853779917	1.00401E-06	Dubious open reading frame
C6_01180C_A	orf19.125	EBP1	3.613781623	1.853509328	4.32493E-31	NADPH oxidoreductase; interacts with phenolic substrates (17beta-estradiol); possible role in estrogen response; induced by oxidative, weak acid stress, NO, benomyl,

						GlcNAc; Cap1, Mnl1 induced; Hap43-repressed; rat catheter biofilm induced
C4_04770C_A	orf19.3803	MNN22	3.551550142	1.828448854	4.07644E-06	Alpha-1, 2-mannosyltransferase; required for normal cell wall mannan; regulated by Tsa1, Tsa1B at 37 deg; repressed in core stress response; NO, Hog1 induced; confers sensitivity to cell wall perturbing agents; Spider biofilm repressed
C5_04940W_A	orf19.3982	orf19.3982	3.540617871	1.824001146	2.77019E-24	Maltase; induced during growth on sucrose; induced by alpha pheromone in SpiderM medium; early-stage flow model biofilm indced
C1_02810W_A	orf19.2969	RAD16	3.492031186	1.804066443	2.58362E-74	Ortholog of S. cerevisiae Rad16; a protein that recognizes and binds damaged DNA; flucytosine induced; rat catheter and Spider biofilm induced
C1_14220C_A	orf19.7231	FTR2	3.44142716	1.783006975	7.03881E-50	High-affinity iron permease; probably interacts with ferrous oxidase; regulated by iron level, ciclopirox olamine, amphotericin B, caspofungin; complements S. cerevisiae ftr1 iron transport defect; Hap43-repressed; Spider biofilm induced
C6_00010W_A	orf19.6339	NRG2	3.430238043	1.778308696	2.04312E-11	Transcription factor; transposon mutation affects filamentous growth
C4_01160W_A	orf19.4674.1	CRD2	3.390251969	1.761392501	2.70731E-16	Metallothionein; for adaptation to growth in high copper; basal transcription is cadmium-repressed; Ssn6 regulated; complements copper sensitivity of an S. cerevisiae cup1 mutant; regulated by Sef1, Sfu1, and Hap43; Spider biofilm induced
C4_01170C_A	orf19.4673	BMT9	3.365980412	1.751026781	1.52307E-23	Beta-mannosyltransferase, 9-gene family that includes characterized genes BMT1, BMT2, BMT3, and BMT4 with roles in beta-1, 2-mannosylation of cell wall

						phosphopeptidomannan; regulated by Sef1, Sfu1, Hap43; rat catheter biofilm repressed
C6_03170C_A	orf19.5604	MDR1	3.349571281	1.743976453	7.73303E-16	Plasma membrane MDR/MFS multidrug efflux pump; methotrexate is preferred substrate; overexpression in drug-resistant clinical isolates confers fluconazole resistance; repressed in young biofilms; rat catheter biofilm induced
C1_03270W_A	orf19.3021	orf19.3021	3.322378289	1.732216349	9.16815E-44	Putative protein of unknown function; Hap43-repressed gene; Spider biofilm induced
C1_06620C_A	orf19.6248	orf19.6248	3.247459168	1.699311386	1.55646E-08	Ortholog of *Candida dubliniensis* CD36: Cd36_06210, *Candida tropicalis* NEW ASSEMBLY: CTRG1_04227, *Candida tropicalis* MYA-3404: CTRG_04227 and *Candida albicans* WO-1: CAWG_00750
C1_05440C_A	orf19.419	orf19.419	3.224861639	1.689237264	5.02583E-45	Protein of unknown function; flow model biofilm induced; Spider biofilm induced
C4_02360W_A	orf19.2768	AMS1	3.162099282	1.660882666	1.13606E-22	Putative alpha-mannosidase; transcript regulated by Nrg1; induced during cell wall regeneration; flow model biofilm induced; Spider biofilm induced
C7_03450C_A	orf19.1331	HSM3	3.131787417	1.646986287	1.8954E-21	Ortholog(s) have role in mismatch repair, proteasome regulatory particle assembly and cytosol, nucleus localization
C1_09340C_A	orf19.4795	orf19.4795	3.120981579	1.641999842	2.41796E-50	Protein of unknown function; Sef1-, Sfu1-, and Hap43 regulated; Spider biofilm induced
C4_07020C_A	orf19.3107	orf19.3107	3.061183764	1.614089653	4.08143E-07	Ortholog of *Candida dubliniensis* CD36: Cd36_46490, *Pichia stipitis* Pignal: PICST_33598, *Candida tropicalis* NEW ASSEMBLY: CTRG1_03758 and *Spathaspora passalidarum* NRRL Y-27907: spas_CGOB_00037
C1_01610C_A	orf19.3351	orf19.3351	3.052345948	1.609918484	2.51715E-37	Protein of unknown function; Hap43-induced; Spider biofilm induced

C6_02100W_A	orf19.3499	orf19.3499	3.048146175	1.607932089	7.02519E-11	Secreted potein; Hap43-repressed; fluconazole-induced; regulated by Tsa1, Tsa1B under H2O2 stress conditions; induced by Mnl1p under weak acid stress; Spider biofilm induced
CR_02200C_A	orf19.3752	RAD51	3.013103335	1.591250151	1.28104E-44	Protein involved in homologous recombination and DNA repair; flucytosine induced; slow growth and increased white-to-opaque switching frequency in null mutant
C2_09880C_A	orf19.1363	orf19.1363	3.005223705	1.587472387	1.6748E-17	Putative protein of unknown function; Plc1-regulated; transcript induced by Mnl1 under weak acid stress; flow model, rat catheter, Spider biofilm induced
C6_02660C_A	orf19.5536	orf19.5536	2.983052322	1.576789283	3.8548E-16	Has domain(s) with predicted carbon-nitrogen ligase activity, with glutamine as amido-N-donor, hydrolase activity
CR_02810W_A	orf19.2839	CIRT4B	2.980522106	1.575565073	0.000263674	Cirt family transposase; transcript repressed in an azole-resistant strain that overexpresses CDR1 and CDR2; Hap43-repressed; flow model biofilm induced
CR_02800C_A	orf19.2838	orf19.2838	2.967478606	1.569237629	5.14494E-12	Protein of unknown function; mutation confers hypersensitivity to amphotericin B; flow model biofilm induced
C7_03460W_A	orf19.1330	orf19.1330	2.90545493	1.538764075	1.3785E-16	Ortholog of *Candida albicans* WO-1: CAWG_05678
CR_04480C_A	orf19.539	LAP3	2.900732768	1.536417392	0.007941438	Putative aminopeptidase; positively regulated by Sfu1; clade-associated gene expression; virulence-group-correlated expression; induced by alpha pheromone in SpiderM medium; Hap43-induced; Spider and flow model biofilm induced
C5_01450W_A	orf19.4135	PRC2	2.895642501	1.533883497	4.80762E-30	Putative carboxypeptidase; induced by human neutrophils; Spider biofilm induced

C4_02660W_A	orf19.2734	orf19.2734	2.891025716	1.531581442	3.11145E-16	Protein with a glucose/ribitol dehydrogenase family domain; mutants are viable
C5_02630C_A	orf19.4279	MNN1	2.851668725	1.511806395	1.10527E-26	Putative alpha-1, 3-mannosyltransferase; of the mannosyltransferase complex; negatively regulated by Rim101; transcript elevated in chk1 and nik1 mutants, but not in sln1 mutant; Spider and flow model biofilm induced
C4_05780C_A	orf19.1264	CFL2	2.842364993	1.507091825	1.02088E-24	Oxidoreductase; iron utilization; Sfu1/Sef1/Hap43/Nrg1/Tup1/Rim101 regulated; alkaline/low iron/fluphenazine/ciclopirox olamine, flucytosine, fluconazole, Spider/flow model/rat catheter biofilm induced; caspofungin/amphotericin B repressed
C7_00110W_A	orf19.7111.1	SOD3	2.824229358	1.497857256	4.1995E-42	Cytosolic manganese-containing superoxide dismutase; protects against oxidative stress; repressed by ciclopirox olamine, induced during stationary phase when SOD1 expression is low; Hap43-repressed; Spider and flow model biofilm induced
C2_10000C_A	orf19.1782.1	orf19.1782.1	2.815962479	1.493628111	2.36995E-11	Ortholog of *Candida parapsilosis* CDC317: CPAR2_407200.1, *Candida dubliniensis* CD36: Cd36_24070, *Candida metapsilosis*: CMET_2070 and *P. stipitis* Pignal: PICST_31669
C2_00760C_A	orf19.2048	orf19.2048	2.787706162	1.479078502	3.10226E-28	Proten of unknown function; transcript positively regulated by Sfu1; Hap43 repressed; Spider biofilm induced
C5_01380W_A	orf19.1930	CFL5	2.783333903	1.476813992	2.32774E-15	Ferric reductase; induced in low iron; ciclopirox olamine, flucytosine induced; amphotericin B, Sfu1 repressed; Tbf1, Hap43 induced
C4_00790C_A	orf19.4162	MLH1	2.744094835	1.456330341	3.50637E-38	Putative mismatch repair protein; cell-cycle regulated periodic mRNA expression

C3_02900W_A	orf19.281	orf19.281	2.726413704	1.447004492	4.11799E-26	Ortholog of *C. dubliniensis* CD36: Cd36_82880, *C. parapsilosis* CDC317: CPAR2_102370, *Candida tenuis* NRRL Y-1498: CANTEDRAFT_118463 and *Pichia stipitis* Pignal: PICST_32241
C3_02990C_A	orf19.295	orf19.295	2.726413704	1.447004492	4.11799E-26	Ortholog of *C. dubliniensis* CD36: Cd36_82970, *C. auris* B8441: B9J08_001198, *Lodderomyces elongisporus* NRLL YB-4239: LELG_02117 and *Debaryomyces hansenii* CBS767: DEHA2G01012g
C2_06130W_A	orf19.4105	CSM3	2.695541707	1.430575232	5.51896E-30	Putative subunit of a replication fork-pausing checkpoint complex
CR_00740C_A	orf19.3282	BMT3	2.695486821	1.430545856	1.9896E-22	Beta-mannosyltransferase; adds 2nd beta-mannose to the acid-stable fraction of cell wall phosphopeptidomannan, elongation of beta-mannose chains on the phosphopeptidomannan acid-labile fraction; Hap43-induced; Spider biofilm induced
C4_00990W_A	orf19.4690	SMF11	2.671698609	1.417757269	3.48842E-07	Putative metal ion transporter of the Nramp family; null mutant shows increased intracellular levels of manganese; induced by nitric oxide independent of Yhb1; flow model biofilm induced; rat catheter biofilm repressed
C1_12840W_A	orf19.4914	orf19.4914	2.667305158	1.415382888	4.90025E-21	Ortholog(s) have role in cellular response to DNA damage stimulus and FANCM-MHF complex localization
C1_00270W_A	orf19.6079	orf19.6079	2.657972312	1.410326076	0.003860314	Predicted ORF in retrotransposon Tca8 with similarity to the Gag region encoding nucleocapsid-like protein; repressed by ciclopirox olamine; filament induced; regulated by Rfg1, Tup1; overlaps orf19.6078.1
C1_05450W_A	orf19.420	orf19.420	2.637658365	1.399257716	0.000170298	Protein of unknown function
C5_04260W_A	orf19.3910	orf19.3910	2.631250119	1.395748394	6.13427E-18	Has domain(s) with predicted RNA binding, ribonuclease T2 activity

C5_01360W_A	orf19.1932	CFL4	2.622619929	1.391008747	4.16101E-37	C-terminus similar to ferric reductases; induced in low iron; Sfu1-repressed; ciclopirox olamine induced; colony morphology-related gene regulation by Ssn6; Hap43-repressed; Sef1-regulated
C2_01010W_A	orf19.2021	HGT8	2.591197504	1.373618984	6.45026E-17	High-affinity glucose transporter of the major facilitator superfamily; 20 members in the C. albicans glucose transporter family; 12 probable membrane-spanning segments; gene has intron; expressed in rich medium; flow model biofilm induced
C6_00020W_A	orf19.6338	orf19.6338	2.58854437	1.372141048	5.54265E-18	Ortholog of *Candida albicans* WO-1: CAWG_05352
C1_14180W_A	orf19.7225	orf19.7225	2.58383473	1.369513794	4.08191E-17	Ortholog of *C. dubliniensis* CD36: Cd36_13150, *C. parapsilosis* CDC317: CPAR2_700610, *C. auris* B8441: B9J08_003318 and *Candida tenuis* NRRL Y-1498: CANTEDRAFT_135418
CR_04220C_A	orf19.510	orf19.510	2.580457572	1.36762691	4.42067E-07	Protein of unknown function; Spider biofilm induced
C1_00310W_A	orf19.6077	orf19.6077	2.578493501	1.366528409	5.0126E-17	Putative protein of unknown function; shows colony morphology-related gene regulation by Ssn6p
C2_08330W_A	orf19.1350	orf19.1350	2.572184756	1.362994273	6.43233E-08	Protein with a thioredoxin domain; predicted role in cell redox homeostasis; rat catheter and Spider biofilm induced
CR_09700W_A	orf19.6592	orf19.6592	2.560732685	1.356556658	1.88498E-14	Predicted membrane transporter, member of the aromatic acid:proton symporter (AAHS) family, major facilitator superfamily (MFS)
CR_09690C_A	orf19.6594	PLB3	2.543840815	1.347008394	7.27698E-18	GPI-anchored cell surface phospholipase B; possibly secreted; fungal-specific (no mammalian homolog); induced by Tbf1; fluconazole-induced; possible essential gene (UAU1 method); Spider and flow model biofilm induced

C1_08070W_A	orf19.5079	CDR4	2.512513137	1.32913114	1.77472E-05	Putative ABC transporter superfamily; fluconazole, Sfu1, Hog1, core stress response induced; caspofungin repressed; fluconazole resistance not affected by mutation or correlated with expression; rat catheter and flow model biofilm induced
C4_07120C_A	orf19.3093	MSH2	2.437900693	1.28563936	1.82787E-34	Putative DNA mismatch repair factor; transcript regulated by Nrg1; flucytosine repressed; transcript regulated by tyrosol and cell density
C4_05530W_A	orf19.1234	FGR6	2.433590164	1.283086227	1.11718E-14	Protein lacking an ortholog in S. cerevisiae; member of a family encoded by FGR6-related genes in the RB2 repeat sequence; transposon mutation affects filamentous growth
C7_03560W_A	orf19.6688	orf19.6688	2.430028782	1.280973401	7.61914E-08	Protein of unknown function; expression decreases by benomyl treatment or in an azole-resistant strain overexpressing MDR1; Spider biofilm induced
C4_06190C_A	orf19.2919	MPH1	2.413254142	1.270979855	6.14204E-25	Protein similar to S. cerevisiae Mph1p, which is a DNA helicase involved in DNA repair; induced under hydroxyurea treatment
C1_13470W_A	orf19.4979	KNS1	2.410087873	1.269085749	8.04125E-10	Protein kinase involved in negative regulation of PolIII transcription; effector kinase of the TOR signaling pathway, phosphorylates Rpc53p to regulate ribosome and tRNA biosynthesis; Spider and flow model biofilm induced
C5_04370C_A	orf19.3923	PGA37	2.410031792	1.269052178	5.42678E-10	Putative GPI-anchored protein; Hap43-repressed; Spider biofilm induced
C1_14030W_A	orf19.7210	orf19.7210	2.407186538	1.267347944	2.05383E-14	Protein of unknown function; Spider biofilm induced
C6_02330W_A	orf19.3475	orf19.3475	2.402732809	1.264676225	2.48093E-08	Described as a Gag-related protein; hyphal induced; downregulation correlates with clinical development of fluconazole resistance; repressed by nitric oxide, 17-beta-estradiol, ethynyl estradiol

C1_07160C_A	orf19.4450.1	orf19.4450.1	2.391706826	1.258040556	7.74761E-12	Protein conserved among the CTG-clade; 2 adjacent upstream SRE-1 elements; highly up-regulated in cecum-grown cells in a Cph2-dependent manner; Hap43-repressed; rat catheter, Spider and flow model biofilm induced
C4_02180C_A	orf19.4573	ZCF26	2.378121886	1.24982266	5.45488E-11	Zn2-Cys6 transcription factor of unknown function; induced by alpha pheromone in SpiderM medium
C6_02940C_A	orf19.5572	orf19.5572	2.369929599	1.244844203	4.9872E-11	Protein of unknown function; Spider biofilm repressed
C1_02150W_A	orf19.3672	GAL10	2.369276294	1.244446449	4.20738E-18	UDP-glucose 4-epimerase; galactose utilization; mutant has cell wall defects and increased filamentation; GlcNAc-, fluconazole- and ketoconazole-induced; stationary phase enriched protein; rat catheter and flow model biofilm induced
CR_02220C_A	orf19.3749	OPT3	2.366196972	1.242570175	1.2996E-14	Oligopeptide transporter; transcript induced by macrophage phagocytosis, BSA or peptides; fluconazole-induced; induced by Rim101 at pH 8; virulence-group-correlated expression; Hap43-repressed; Spider biofilm induced
C2_07630C_A	orf19.1862	orf19.1862	2.34788622	1.231362496	3.25167E-05	Possible stress protein; increased transcription associated with CDR1 and CDR2 overexpression or fluphenazine treatment; regulated by Sfu1, Nrg1, Tup1; stationary phase enriched protein; Spider biofilm induced
CR_07800W_A	orf19.3708	SAP2	2.344306882	1.229161438	4.74137E-07	Major secreted aspartyl proteinase; utilization of protein as nitrogen source; role in virulence complicated by URA3 effects; immunoprotective; regulated by growth, albumin, drugs, white cell-type; flow model biofilm induced
C2_01840C_A	orf19.1493	RAD7	2.343521913	1.228678285	1.9976E-30	Protein similar to S. cerevisiae Rad7p, which is a subunit of the Nucleotide Excision Repair Factor 4; induced under hydroxyurea treatment

C1_13180W_A	orf19.4946	orf19.4946	2.335450088	1.223700613	0.000117029	Ortholog of *C. dubliniensis* CD36: Cd36_12240, *C. parapsilosis* CDC317: CPAR2_801750, *C. auris* B8441: B9J08_002031, *C. tenuis* NRRL Y-1498: CANTEDRAFT_135403 and *Pichia stipitis* Pignal: PICST_29111
C2_04420W_A	orf19.4510	IFA4	2.330468067	1.220619745	7.81568E-21	Protein of unknown function; oxidative stress-induced via Cap1
C1_11700C_A	orf19.1149	MRF1	2.329327434	1.219913454	3.88132E-13	Putative mitochondrial respiratory protein; induced by farnesol, benomyl, nitric oxide, core stress response; oxidative stress-induced via Cap1; stationary-phase enriched protein; Spider biofilm induced
C4_04850C_A	orf19.3794	CSR1	2.327980002	1.219078665	4.98251E-17	Transcription factor; role in zinc homeostasis and regulation of Spider biofilm matrix; mutation affects filamentous growth; can suppress S. cerevisiae rok1 mutant inviability; Spider biofilm induced; mutants for abnormal Spider biofilms
C1_12850W_A	orf19.4914.1	BLP1	2.325283976	1.217406917	5.09096E-12	Protein of unknown function, serum-induced
C4_02330C_A	orf19.2770	orf19.2770	2.317843817	1.212783357	0.014628719	Ortholog of *C. dubliniensis* CD36: Cd36_42220, *C. parapsilosis* CDC317: CPAR2_500340, *Debaryomyces hansenii* CBS767: DEHA2G17754g and *Pichia stipitis* Pignal: PICST_31424
C6_04130C_A	orf19.4555	ALS4	2.309430314	1.207537014	2.67464E-09	GPI-anchored adhesin; role in adhesion, germ tube induction; growth, temperature regulated; expressed during infection of human buccal epithelial cells; repressed by vaginal contact; biofilm induced; repressed during chlamydospore formation
CR_06500C_A	orf19.716	orf19.716	2.298171995	1.200486773	3.14525E-08	Protein of unknown function; Hap43-induced; regulated by Nrg1, Tup1; repressed by alpha pheromone in SpiderM

						medium; Spider biofilm induced; Bcr1-repressed in RPMI a/a biofilms
C7_00880C_A	orf19.7027	orf19.7027	2.293574999	1.197598084	2.44009E-11	Protein of unknown function; Spider biofilm induced
C4_00440C_A	orf19.5673	OPT7	2.285704643	1.192638991	3.30205E-14	Putative oligopeptide transporter; possibly transports GSH or related compounds; Hog1-induced; expression of OPT6, -7, or -8 does not suppress defect of mutant lacking OPT1-3; Hap43-repressed; F-12/CO2 early biofilm induced
CR_10790W_A	orf19.7668	MAL2	2.280141452	1.189123327	3.00694E-16	Alpha-glucosidase; hydrolyzes sucrose for sucrose utilization; transcript regulated by Suc1, induced by maltose, repressed by glucose; Tn mutation affects filamentous growth; upregulated in RHE model; rat catheter and Spider biofilm induced
C2_09130C_A	orf19.4072	IFF6	2.275593812	1.186243063	1.71506E-14	Putative GPI-anchored adhesin-like protein; opaque-specific transcript; macrophage-induced gene; Hap43-repressed gene; Spider biofilm induced
C1_13490C_A	orf19.4981	orf19.4981	2.270485419	1.183000772	4.58094E-16	Ortholog(s) have role in cellular calcium ion homeostasis, regulation of G0 to G1 transition
C3_05610W_A	orf19.6996	MNN14	2.267438261	1.181063269	9.94522E-23	Predicted alpha-1, 3-mannosyltransferase activity with a role in protein glycosylation; Hap43-repressed; Spider biofilm induced
CR_06550C_A	orf19.711	orf19.711	2.246276434	1.167535481	3.57272E-05	Protein of unknown function; induced by nitric oxide; predicted ORF from Assembly 19; removed from Assembly 20; restored based on transcription data
C4_00430W_A	orf19.5672	MEP2	2.238897649	1.162788577	4.81419E-21	Ammonium permease and regulator of nitrogen starvation-induced filamentation; 11 predicted transmembrane regions; in low nitrogen cytoplasmic C-terminus activates Ras/cAMP and MAPK signal transduction pathways to induce filamentation

C2_00940W_A	orf19.2030	orf19.2030	2.236918492	1.161512689	1.65952E-12	Plasma membrane-associated protein; induced in cyr1 or ras1 mutant; induced by hypoxia, ketoconazole and during growth in the mouse cecum; induced in oralpharyngeal candidasis; Spider biofilm induced
C6_01680C_A	orf19.3418	GBU1	2.22687187	1.15501855	2.14734E-07	Guanidinobutyrase (Gbase), enzyme involved in metabolism of guanidinobutyrate
C3_03700C_A	orf19.6949	orf19.6949	2.20743894	1.142373532	0.000322496	Ortholog of *Candida albicans* WO-1: CAWG_02687
C2_08510W_A	orf19.3627	orf19.3627	2.202055263	1.138850675	1.60135E-15	Ortholog of *C. dubliniensis* CD36: Cd36_22640, *C. parapsilosis* CDC317: CPAR2_406910, *C. auris* B844: B9J08_004017 and *C. tenuis* NRRL Y-1498: CANTEDRAFT_104937
C7_01130C_A	orf19.6897	orf19.6897	2.194739193	1.13404951	0.025669506	Protein of unknown function
CR_05570C_A	orf19.5845	RNR3	2.187016445	1.128964069	1.14054E-12	Putative ribonucleotide reductase large subunit; transcript induced in low iron; rat catheter and Spider biofilm induced
CR_03920C_A	orf19.473	TPO4	2.185285438	1.127821734	3.52632E-19	Putative sperimidine transporter; fungal-specific (no human or murine homolog); Spider biofilm induced; promoter bound by Tec1 and Ndt80; Bcr1-repressed in RPMI a/a biofilms
C4_05350W_A	orf19.1793	orf19.1793	2.176477447	1.121995071	1.46961E-12	Ortholog(s) have phosphatidylinositol-3, 5-bisphosphate binding, phosphatidylinositol-3-phosphate binding, phosphatidylinositol-4-phosphate binding, phosphatidylinositol-5-phosphate binding activity
C1_10800C_A	orf19.2337	ALP1	2.175474488	1.121330099	8.66977E-13	Cystine transporter; present in pathogenic yeasts (no human or murine homolog); Spider biofilm induced
C1_04430C_A	orf19.5194.1	orf19.5194.1	2.175269027	1.121193838	7.51023E-16	Putative protein of unknown function; clade-associated gene expression
C4_02030W_A	orf19.4590	RFX2	2.17119703	1.118490652	3.42378E-14	Transcriptional repressor; regulator of filamentation, response to DNA damage, adhesion, virulence in murine

						mucosal, systemic infections; RFX domain; regulated by Nrg1, UV-induced; partially complements S. cerevisiae rfx1 mutant defects
C1_03990W_A	orf19.4474	orf19.4474	2.170814374	1.118236367	6.10497E-23	Ortholog(s) have proteasome binding activity and role in cellular response to arsenic-containing substance, proteasome-mediated ubiquitin-dependent protein catabolic process
C1_05840W_A	orf19.2467	PRN1	2.170409616	1.117967345	7.64819E-08	Protein with similarity to pirins; induced by benomyl and in response to alpha pheromone in SpiderM medium; transcript induced by Mnl1 in weak acid stress; rat catheter and Spider biofilm induced
C1_07080W_A	orf19.6196	RTT102	2.16398568	1.113690952	6.23746E-11	Component of the SWI/SNF and RSC chromatin remodeling complexes; suggested role in chromosome maintenance; Spider biofilm induced
C2_00770W_A	orf19.2047	orf19.2047	2.156289613	1.10855096	7.88964E-11	Putative protein of unknown function; Hap43p-repressed gene; mutation confers hypersensitivity to toxic ergosterol analog, and to amphotericin B
C6_03250W_A	orf19.5612	BMT4	2.141084991	1.098342065	3.04013E-13	Beta-mannosyltransferase; for elongation of beta-mannose chains on the acid-labile fraction of cell wall phosphopeptidomannan; 9-gene family member; regulated by Tsa1, Tsa1B; flow model biofilm induced; rat catheter biofilm repressed
C7_03170W_A	orf19.5138	IFA21	2.140380884	1.097867549	1.1254E-19	IPF family A protein; mutants are viable; Spider biofilm induced
C2_04720C_A	orf19.156	FGR51	2.137406833	1.095861536	7.55895E-27	Protein lacking an ortholog in S. cerevisiae; transposon mutation affects filamentous growth; Hap43p-repressed gene

C6_01870C_A	orf19.3395	orf19.3395	2.132243415	1.092372144	1.86352E-08	Predicted MFS membrane transporter, member of the drug:proton antiporter (12 spanner) (DHA1) family; induced by nitric oxide, oxidative stress, alpha pheromone; fungal-specific; Hap43-repressed; Spider biofilm induced
C4_01240C_A	orf19.4665	orf19.4665	2.129413003	1.09045579	1.31617E-13	Protein of unknown function; Spider biofilm induced
C2_09120C_A	orf19.4070	orf19.4070	2.122842245	1.085997164	6.09414E-06	Ortholog of *C. dubliniensis* CD36: Cd36_23270
CR_05340C_A	orf19.5288	IFE2	2.115305195	1.08086583	1.35591E-12	Putative alcohol dehydrogenase; yeast-enriched transcript; Efg1-regulated; induced by prostaglandins, Hog1, fluconazole; rat catheter biofilm induced
C1_00290W_A	orf19.6078	POL93	2.108089042	1.075935805	0.015775161	Predicted ORF in retrotransposon Tca8 with similarity to the Pol region of retrotransposons encoding reverse transcriptase, protease and integrase; downregulated in response to ciclopirox olamine; F-12/CO2 early biofilm induced
C1_02250W_A	orf19.3682	CWH8	2.096140237	1.06773524	5.45453E-08	Putative dolichyl pyrophosphate (Dol-P-P) phosphatase; ketoconazole-induced; expression is increased in a fluconazole-resistant isolate; clade-associated gene expression; Hap43p-induced gene
C3_06450W_A	orf19.7434	GLG2	2.094445199	1.066568137	2.66656E-16	Putative self-glucosylating initiator of glycogen synthesis; expression regulated upon white-opaque switch; hypha-induced; Spider biofilm induced
CR_05420W_A	orf19.5284	orf19.5284	2.087669853	1.06189358	0.000335773	Dubious open reading frame
CR_01200W_A	orf19.3234	OYE22	2.086383824	1.06100459	3.11738E-07	Putative NADPH dehydrogenase; rat catheter biofilm induced
C1_10300W_A	orf19.4900	MNN12	2.075355365	1.053358392	3.51441E-12	Predicted alpha-1, 3-mannosyltransferase activity with a role in protein glycosylation
C6_03160C_A	orf19.5602	BMT6	2.062622293	1.04447966	1.66562E-11	Beta-mannosyltransferase; beta-1, 2-mannosylation of phospholipomannan; member of a 9-member family

						including Bmt1, Bmt2, Bmt3, and Bmt4 with roles in mannosylation of cell wall phosphopeptidomannan; flow model biofilm induced
CR_08310C_A	orf19.6398	orf19.6398	2.062326343	1.044272643	0.000107738	S. pombe ortholog SPBC460.04c is a predicted sulfonate/alpha-ketoglutare dioxygenase; induced by nitric oxide; Spider biofilm induced
C1_09290C_A	orf19.4788	ARG5	2.056165125	1.039956128	7.40371E-17	Arginine biosynthetic enzyme; processed in S. cerevisiae into 2 polypeptides with acetylglutamate kinase (Arg6) activity and acetylglutamate-phosphate reductase (Arg5) activity; Gcn4 regulated; alkaline repressed; Spider biofilm induced
C4_02560C_A	orf19.2745	UME7	2.050972316	1.036308018	3.93104E-05	Putative transcription factor with zinc cluster DNA-binding motif; similar to S. cerevisiae Ume6p, which is a transcription factor involved in the regulation of meiotic genes
C1_07220W_A	orf19.4445	orf19.4445	2.046234681	1.032971616	2.32524E-07	Protein of unknown function; Plc1p-regulated; expression induced early upon infection of reconstituted human epithelium (RHE), while expression of the C. dubliniensis ortholog is not; mutant is viable; Spider biofilm induced
C4_02590C_A	orf19.2740	SLX4	2.03981865	1.028440895	1.13987E-07	Putative endonuclease involved in DNA repair
C5_04190W_A	orf19.3902	MRV2	2.039030175	1.027883126	0.001257776	Protein of unknown function; repressed by fluphenazine treatment or in an azole-resistant strain that overexpresses CDR1 and CDR2; Spider biofilm induced
C3_04330C_A	orf19.5876	orf19.5876	2.038985204	1.027851306	6.13892E-13	Protein of unknown function; Cyr1-repressed; induced by alpha pheromone in SpiderM medium; rat catheter and Spider biofilm induced

C2_00750W_A	orf19.2049	orf19.2049	2.034856661	1.024927172	6.69735E-07	Plasma membrane-associated protein; heterozygous null mutant displays sensitivity to virgineone; Spider biofilm induced
C1_03620C_A	orf19.3061.1	orf19.3061.1	2.027506159	1.019706297	2.05747E-18	Ortholog of S. cerevisiae Rps22Ap and Rps22Bp; gene contains 5' UTR intron
C1_10360C_A	orf19.4907	orf19.4907	2.027301192	1.019560443	0.002375383	Putative protein of unknown function; Hap43p-repressed gene; increased transcription is observed upon fluphenazine treatment; possibly transcriptionally regulated by Tac1p; induced by nitric oxide; fungal-specific (no human/murine homolog
C1_14190C_A	orf19.7227	orf19.7227	2.021657785	1.015538806	0.005416126	Protein phosphatase inhibitor; Hap43-repressed; homozygous Tn insertion decreases colony wrinkling but does not block hyphal growth in liquid media; mutation confers hypersensitivity to toxic ergosterol analog; Spider biofilm induced
C3_01230C_A	orf19.1728	orf19.1728	2.016648115	1.01195937	0.029534317	Ortholog of *C. dubliniensis* CD36: Cd36_81190, *C. parapsilosis* CDC317: CPAR2_503840, *Debaryomyces hansenii* CBS76: DEHA2A09042g and *Pichia stipitis* Pignal: PICST_42519
C1_00780C_A	orf19.6028	HGC1	2.015462571	1.011110991	8.53282E-10	Hypha-specific G1 cyclin-related protein involved in regulation of morphogenesis, biofilm formation; Cdc28-Hgc1 maintains Cdc11 S394 phosphorylation during hyphal growth; required for virulence in mice; regulated by Nrg1, Tup1, farnesol
C4_06020C_A	orf19.4412	orf19.4412	2.013912387	1.010000922	6.06027E-23	Ortholog(s) have DNA-directed DNA polymerase activity, deoxycytidyl transferase activity and role in cellular response to DNA damage stimulus, error-free translesion synthesis, error-prone translesion synthesis

C3_03230C_A	orf19.321	orf19.321	2.011408401	1.008206039	1.28185E-12	Ortholog(s) have L-methionine transmembrane transporter activity and role in methionine import across plasma membrane
C1_11790W_A	orf19.1137	orf19.1137	2.004030381	1.00290438	7.49302E-15	Thymidylate kinase of unknown role; forms a dimer; potential target for antifungal drugs
C2_06880C_A	orf19.2242	PRB1	2.000561718	1.000405137	4.21033E-16	Endoprotease B; regulated by heat, carbon source (GlcNAc-induced), nitrogen, macrophage response, human neutrophils; similar to (does not replace) S. cerevisiae vacuolar B protease Prb1p; flow model biofilm induced; Spider biofilm induced
C4_00390W_A	orf19.5666	orf19.5666	1.999838957	0.999883827	3.20738E-15	Ortholog(s) have chromatin binding activity
CR_09100C_A	orf19.7306	orf19.7306	1.993025764	0.99496036	9.4793E-12	Aldo-keto reductase; increased transcript associated with MDR1 overexpression, benomyl or long-term fluconazole treatment; overexpression does not affect drug or oxidative stress sensitivity; stationary phase enriched; flow biofim repressed
C2_07640W_A	orf19.1861	orf19.1861	1.986474649	0.990210382	3.59231E-12	BAR domain-containing protein, forms heterodimer with Rvs162p that binds liposomes in vitro; flow model biofilm induced
C5_03510C_A	orf19.6660	orf19.6660	1.985582278	0.989562144	1.11232E-11	Protein of unknown function; mRNA binds to She3; Hap43-repressed; rat catheter and flow model biofilm induced
C1_01830C_A	orf19.4540	UBC8	1.98412041	0.988499581	1.83579E-19	Predicted ubiquitin-conjugating enzyme that negatively regulates gluconeogenesis by mediating the glucose-induced ubiquitination of fructose-1, 6-bisphosphatase; induced by alpha pheromone in SpiderM medium
CR_03040C_A	orf19.2425	HGT18	1.982797662	0.987537463	9.10476E-10	Putative glucose transporter of the major facilitator superfamily; the C. albicans glucose transporter family

						comprises 20 members; 12 probable membrane-spanning segments; expressed in rich medium with 2% glucose
C4_04140W_A	orf19.5295	orf19.5295	1.980382136	0.985778841	2.04172E-07	Protein with a predicted endonuclease/exonuclease/phosphatase family domain and a carbon catabolite repressor protein 4 domain; induced by alpha pheromone in SpiderM medium
C7_03570W_A	orf19.6689	ARG4	1.979460486	0.985107269	3.72642E-08	Argininosuccinate lyase, catalyzes the final step in the arginine biosynthesis pathway; alkaline downregulated; flow model biofilm induced; Spider biofilm induced
C4_04050C_A	orf19.5305	RHD3	1.971606494	0.979371638	3.26935E-12	GPI-anchored yeast-associated cell wall protein; induced in high iron; clade-associated gene expression; not essential for cell wall integrity; fluconazole-repressed; flow model and Spider biofilm repressed
C4_04670C_A	orf19.3813	orf19.3813	1.966216598	0.975422257	8.73313E-15	Ortholog of *C. dubliniensis* CD36: Cd36_44340, *C. parapsilosis* CDC317: CPAR2_302240, *Candida tenuis* NRRL Y-1498: CANTEDRAFT_105331 and *Debaryomyces hansenii* CBS767: DEHA2E03454g
C1_08130C_A	orf19.5089	TERT	1.957235447	0.968817316	6.64526E-16	Telomerase reverse transcriptase; catalytic protein subunit of telomere synthesis; essential for telomerase activity; has telomerase-specific motif T and other conserved reverse transcriptase motifs
C7_03140W_A	orf19.5141	orf19.5141	1.95435577	0.966693119	3.26935E-12	Ortholog of *Candida albicans* WO-1: CAWG_05647
CR_04030W_A	orf19.486	NIP100	1.947265624	0.961449694	3.23856E-16	p150 subunit of dynactin; required for normal spindle formation and position
CR_03480W_A	orf19.4391	orf19.4391	1.947148046	0.96136258	9.31574E-08	Ortholog of *C. dubliniensis* CD36: Cd36_28730, *Candida tropicalis* NEW ASSEMBLY: CTRG1_00749, *Candida tropicalis* MYA-3404: CTRG_00749 and *Candida albicans* WO-1: CAWG_01683

C1_05700W_A	orf19.2480.1	AUT7	1.94612927	0.960607543	5.28434E-09	Putative autophagosome protein; acts synergistically with Ysy6p to regulate unfolded protein response and mitochondrial function under ER stress; macrophage/pseudohyphal-repressed; alternatively spliced intron in 5' UTR
CR_04440C_A	orf19.535	RBR1	1.945476514	0.960123564	0.000634044	Glycosylphosphatidylinositol (GPI)-anchored cell wall protein; required for filamentous growth at acidic pH; expression repressed by Rim101 and activated by Nrg1; Hap43-induced
C1_09240C_A	orf19.4783	orf19.4783	1.938829377	0.955185848	0.000400274	Protein of unknown function; induced during chlamydospore formation in both C. albicans and C. dubliniensis
C6_01940W_A	orf19.685.1	orf19.685.1	1.935288872	0.952548927	0.000543634	Ortholog of *C. parapsilosis* CDC317: CPAR2_602135, *Candida tenuis* NRRL Y-1498: cten_CGOB_00042, *Debaryomyces hansenii* CBS767: DEHA2F11858g and *Pichia stipitis* Pignal: PICST_60705
C2_08110W_A	orf19.2174	RAD57	1.93314818	0.950952227	8.62184E-20	Putative DNA recombination and repair protein; induced by interaction with macrophage; transcript is regulated by Nrg1, Mig1, and Tup1; essential protein; S. cerevisiae ortholog is essential
C1_02630C_A	orf19.2952	EXG2	1.931914107	0.950030953	1.89941E-08	GPI-anchored cell wall protein, similar to S. cerevisiae exo-1, 3-beta-glucosidase Exg2p; predicted Kex2p substrate; induced during cell wall regeneration; possibly an essential gene, disruptants not obtained by UAU1 method; Hap43p-repressed
C6_01960W_A	orf19.687	orf19.687	1.927141204	0.946462283	4.69324E-15	Ortholog of *C. dubliniensis* CD36: Cd36_62090, *C. parapsilosis* CDC317: CPAR2_602150, *C. auris* B8441:

						B9J08_005503 and *Candida tenuis* NRRL Y-1498: CANTEDRAFT_112751
C1_09670C_A	orf19.4830	orf19.4830	1.926847422	0.946242336	0.000871813	Has domain(s) with predicted DNA binding activity, role in DNA recombination, DNA repair, DNA replication and nucleus localization
C2_10840W_A	orf19.5367	RDH54	1.922998784	0.943357851	2.53177E-14	Putative DNA-dependent ATPase with a predicted role in DNA recombination and repair; transcriptionally induced by interaction with macrophages
C4_05380C_A	orf19.1795.1	orf19.1795.1	1.920765694	0.941681542	0.011096332	Ortholog(s) have role in protein targeting to ER, signal peptide processing and signal peptidase complex localization
C1_14050C_A	orf19.7213	orf19.7213	1.918147487	0.939713654	1.61329E-19	Putative ATP-dependent RNA helicase; fungal-specific (no human or murine homolog)
C7_00420C_A	orf19.7078	orf19.7078	1.906130376	0.930646801	2.75018E-06	Ortholog of *S. cerevisiae*: YCL012C, *C. dubliniensis* CD36: Cd36_65270, *C. parapsilosis* CDC317: CPAR2_805430, *C. auris* B8441: B9J08_000227 and *Candida tenuis* NRRL Y-1498: CANTEDRAFT_96597
CR_07480W_A	orf19.6117	orf19.6117	1.900441894	0.926334915	7.74761E-12	S. pombe ortholog SPAC5D6.04 is a predicted auxin family transmembrane transporter; ketoconazole and hypoxia induced
C6_02480W_A	orf19.5517	orf19.5517	1.900308349	0.926233533	8.095E-10	Similar to alcohol dehydrogenases; induced by benomyl treatment, nitric oxide; induced in core stress response; oxidative stress-induced via Cap1; Spider biofilm repressed
CR_06510W_A	orf19.715	orf19.715	1.899370061	0.925521018	7.69509E-06	Protein of unknown function; Hap43-induced; rat catheter and Spider biofilm induced
C4_03880W_A	orf19.5043	orf19.5043	1.899166101	0.925366089	1.86579E-08	Ortholog of *C. dubliniensis* CD36: Cd36_43610, *C. parapsilosis* CDC317: CPAR2_403770, *C. auris* B8441:

						B9J08_000308, *Debaryomyces hansenii* CBS767: DEHA2G16962g and *Pichia stipitis* Pignal: PICST_30926
C1_02730W_A	orf19.2962	orf19.2962	1.89687049	0.923621181	3.96687E-07	Protein of unknown function; Spider biofilm induced
C3_07070C_A	orf19.6793	orf19.6793	1.89470739	0.921975062	7.74291E-08	Protein of unknown function; Sef1, Sfu1, and Hap43 regulated; rat catheter and Spider biofilm induced
C1_13660W_A	orf19.5004	RAD54	1.888140424	0.916966064	1.50278E-14	Putative DNA-dependent ATPase involved in DNA repair; induced under hydroxyurea treatment; plays an essential role during mitotic growth; mutants display aberrant cell and nuclear morphology
C2_09860C_A	orf19.1365	orf19.1365	1.8850161	0.914576845	4.08747E-06	Putative monooxygenase; mutation confers hypersensitivity to toxic ergosterol analog; constitutive expression independent of MTL or white-opaque status
CR_00170W_A	orf19.7518	ZCF38	1.88029983	0.910962731	1.46961E-12	Putative Zn(II)2Cys6 transcription factor
C5_00140C_A	orf19.5683	orf19.5683	1.876305985	0.907895119	1.61354E-06	Putative integral membrane protein of unknown function; clade-associated gene expression; Spider biofilm induced
C1_04330W_A	orf19.1069	RPN4	1.874367062	0.906403507	1.13323E-15	C2H2 transcription factor; regulator of proteasome genes; induced by Hap43, Spider biofilm, and within core stress response; null mutants show increased susceptibility to fluconazole and kill macrophages more slowly than wild type
C5_05350W_A	orf19.4030	orf19.4030	1.87401158	0.906129868	2.21854E-21	Ortholog(s) have DNA primase activity, single-stranded DNA binding activity and role in DNA replication, DNA replication, synthesis of RNA primer
CR_09050C_A	orf19.7301	orf19.7301	1.873003354	0.905353483	1.26596E-07	Has domain(s) with predicted DNA binding, nucleic acid binding activity
C4_05440C_A	orf19.1800	orf19.1800	1.87090475	0.903736111	3.86034E-09	Protein of unknown function; Spider biofilm induced
C2_03220C_A	orf19.909	STP4	1.869974722	0.903018768	2.54335E-10	C2H2 transcription factor; induced in core caspofungin response; colony morphology-related gene regulation by

						Ssn6; induced by 17-beta-estradiol, ethynyl estradiol; rat catheter and Spider biofilm induced
C1_00860W_A	orf19.6020	orf19.6020	1.864947459	0.899134987	3.66701E-13	Ortholog(s) have Atg8 ligase activity
CR_03120W_A	orf19.2414	orf19.2414	1.861801029	0.8966989	3.14451E-09	Ortholog of S. cerevisiae Mpm1; a mitochondrial intermembrane space protein of unknown function; Hap43-repressed; Spider biofilm induced
C1_09400C_A	orf19.4802	FTH1	1.858853676	0.89441321	3.8465E-14	Putative iron permease involved in the production of prostaglandin E2; mutants show decreased metabolic activity in biofilms
C7_03580C_A	orf19.6690	orf19.6690	1.856865046	0.892868967	2.76315E-09	Protein of unknown function; Hap43-repressed gene
C7_03680W_A	orf19.6703	orf19.6703	1.85475697	0.891230162	3.20951E-13	Ortholog of *C. dubliniensis* CD36: Cd36_73260 and *Candida albicans* WO-1: CAWG_05699
C6_01430C_A	orf19.3441	FRP6	1.85225875	0.889285649	2.199E-12	Putative ammonia transport protein; regulated by Nrg1 and Tup1; regulated by Ssn6; induced by human neutrophils
C6_00510C_A	orf19.4208	RAD52	1.847935389	0.885914315	6.70753E-12	Required for homologous DNA recombination, repair of UV- or MMS-damaged DNA, telomere length, UV-induced LOH; constitutive expression, MMS-induced; weakly complements S. cerevisiae rad52 mutant; slow growth, increased white-to-opaque switch
C1_14060W_A	orf19.7214	orf19.7214	1.842084397	0.881339162	5.23272E-09	Glucan 1, 3-beta-glucosidase; regulated by Nrg1, Tup1 and possibly Tac1; induced by NO and during cell wall regeneration; stationary phase enriched; possibly essential (UAU1 method); F-12/CO2 early biofilm induced; flow biofilm repressed
C4_06780C_A	orf19.3131	OYE32	1.841223516	0.880664774	1.92163E-10	NAD(P)H oxidoreductase family protein; induced by nitric oxide, amphotericin B, oxidative stress via Cap1; upregulation associated with MDR1 overexpression or

						benomyl treatment; macrophage-downregulated protein; Spider biofilm induced
C3_02390W_A	orf19.1606	orf19.1606	1.837131274	0.877454719	5.82624E-13	Protein of unknown function; Plc1-regulated
C3_06270C_A	orf19.7405	orf19.7405	1.836408597	0.876887091	2.08477E-08	Ortholog of Rad33; involved in nucleotide excision repair in S. cerevisiae; induced by Mnl1 under weak acid stress
C1_00830W_A	orf19.6023	orf19.6023	1.835765542	0.876381814	1.27258E-07	Protein with a predicted multidrug transporter domain; Hap43-repressed gene
C3_04170W_A	orf19.5860	orf19.5860	1.832764522	0.874021437	0.005802275	Predicted ORF from Assembly 19; removed from Assembly 20; subsequently reinstated in Assembly 21 based on comparative genome analysis
C4_02610C_A	orf19.2738	SUL2	1.830393479	0.872153818	4.03549E-07	Putative sulfate transporter; transcript negatively regulated by Sfu1; amphotericin B induced; F-12/CO2 and Spider biofilm induced
C4_06320C_A	orf19.2905	orf19.2905	1.8290579	0.871100745	0.000329749	Ortholog of *Candida albicans* WO-1: CAWG_03194
C5_04560C_A	orf19.3940.1	CUP1	1.828834906	0.870924845	4.8769E-09	Metallothionein; involved in copper resistance; copper induced; Spider biofilm induced; flow model biofilm repressed
C3_00180C_A	orf19.5454	DAL1	1.827606101	0.869955164	6.96111E-10	Putative allantoinase; transcript regulated by Nrg1 and Mig1; macrophage/pseudohyphal-repressed
C4_02340W_A	orf19.2769	orf19.2769	1.822511535	0.865927946	2.81873E-07	Putative protease B inhibitor; hyphal-induced expression; Cyr1p- and Ras1p-repressed
C4_04470W_A	orf19.3839	SAP10	1.819939355	0.863890377	6.44324E-08	Secreted aspartyl protease; roles in adhesion, virulence (RHE model), cell surface integrity; distinct specificity from Sap9; at cell membrane and wall; GPI-anchored; induced in low iron; Tbf1-activated; Spider biofilm induced
C1_01710W_A	orf19.3360	orf19.3360	1.818625613	0.862848577	8.94178E-08	Protein of unknown function; flow model biofilm induced; Spider biofilm induced

C5_00010W_A	orf19.5700	TLO11	1.816858092	0.86144574	0.026139655	Member of a family of telomere-proximal genes of unknown function; may be spliced in vivo
C1_10560C_A	orf19.1002	orf19.1002	1.816160524	0.860891723	1.19329E-08	Protein of unknown function; Hap43-repressed gene
CR_06560C_A	orf19.916	YBH3	1.816112097	0.860853254	1.16496E-07	Protein that promotes apoptosis and negatively regulates filamentation; null mutant shows increased virulence and hyperfilamentation; Spider biofilm induced
CR_06730W_A	orf19.707	APG7	1.813396839	0.858694675	4.93234E-12	Ortholog(s) have Atg12 activating enzyme activity, Atg8 activating enzyme activity
C2_05660W_A	orf19.6877	PNG2	1.812490419	0.857973369	1.47042E-10	Putative peptide:N-glycanase; gene has variable numbers of 12-bp repeats; induced by caspofungin, ciclopirox olamine, ketoconazole or hypoxia; gene of core caspofungin response; Hap43-induced; Spider biofilm induced
C6_00680C_A	orf19.4192.1	orf19.4192.1	1.810750025	0.856587395	1.13969E-12	Ortholog of *C. dubliniensis* CD36: Cd36_60590, *C. parapsilosis* CDC317: CPAR2_602880, *C. auris* B8441: B9J08_001867 and *Candida tenuis* NRRL Y-1498: CANTEDRAFT_115034
C7_00600C_A	orf19.7059	orf19.7059	1.810563006	0.856438382	3.39997E-13	Ortholog(s) have endoribonuclease activity, role in nuclear mRNA surveillance of mRNP export, transcription elongation from RNA polymerase II promoter and cytoplasm, nucleus localization
C1_10730W_A	orf19.2346	orf19.2346	1.80416867	0.851334221	2.8951E-19	Putative protein of unknown function, transcription is positively regulated by Tbf1p
C3_05080W_A	orf19.5978	orf19.5978	1.803819085	0.85105465	0.000978777	Has domain(s) with predicted acyltransferase activity, transferring groups other than amino-acyl groups, oxidoreductase activity, zinc ion binding activity

C5_02520W_A	orf19.4264	orf19.4264	1.803608309	0.850886062	1.6689E-05	Protein of unknown function; induced during chlamydospore formation in both C. albicans and C. dubliniensis; flow model biofilm induced
C1_10520W_A	orf19.996	orf19.996	1.803377451	0.850701388	8.70859E-07	Protein with a predicted leucine-rich repeat domain; possibly an essential gene, disruptants not obtained by UAU1 method
C2_05510C_A	orf19.3581	orf19.3581	1.796975988	0.845571131	1.71438E-08	Ortholog(s) have histone binding activity, role in DNA replication-dependent nucleosome assembly and CAF-1 complex, cytoplasm, nucleus localization
C4_03960W_A	orf19.787.1	orf19.787.1	1.796779457	0.845413338	0.001486333	Protein of unknown function; ORF added to Assembly 21 based on comparative genome analysis; protein detected by mass spec in stationary phase cultures
C5_02160W_A	orf19.4222	SST2	1.796010749	0.844795984	1.13769E-06	Predicted regulator of G-protein signaling in mating pathway; null mutation causes alpha-factor hypersensitivity and mating defect (in opaque MTLa/MTLa background); transcript induced by alpha factor
C1_02180W_A	orf19.3675	GAL7	1.79399364	0.843174776	3.07431E-08	Putative galactose-1-phoshphate uridyl transferase; downregulated by hypoxia, upregulated by ketoconazole; macrophage/pseudohyphal-repressed
C3_04890W_A	orf19.5958	CDR2	1.793235977	0.842565349	3.11599E-11	Multidrug transporter, ATP-binding cassette (ABC) superfamily; transports phospholipids, in-to-out direction; overexpressed in azole-resistant isolates; repressed in young biofilms
C1_03750W_A	orf19.1034	orf19.1034	1.789173446	0.839293252	8.86016E-10	Protein with a predicted cytochrome b5-like Heme/Steroid binding domain; Hap43, caspofungin repressed; flow model biofilm induced
C3_07970C_A	orf19.6191	TLO8	1.785482159	0.836313718	1.36128E-08	Member of a family of telomere-proximal genes of unknown function; may be spliced in vivo

C2_09980W_A	orf19.1785	orf19.1785	1.778730573	0.830848	1.0308E-05	Protein with a PI31 proteasome regulator domain; Hap43-repressed; flow model biofilm induced
C2_06720W_A	orf19.3150	GRE2	1.776907334	0.829368447	3.01283E-06	Putative reductase; Nrg1 and Tup1-regulated; benomyl- and hyphal-induced; macrophage/pseudohyphal-repressed; repressed by low iron; possibly involved in osmotic stress response; stationary phase enriched protein; Spider biofilm induced
C7_02750W_A	orf19.5191	FGR6	1.770134248	0.823858779	1.2064E-05	Protein lacking an ortholog in S. cerevisiae; member of a family encoded by FGR6-related genes in the RB2 repeat sequence; transposon mutation affects filamentous growth
C1_09160W_A	orf19.4774	AOX1	1.768052809	0.822161366	1.3045E-05	Alternative oxidase; low abundance; constitutively expressed; one of two isoforms (Aox1p and Aox2p); involved in a cyanide-resistant respiratory pathway present in plants, protists, and some fungi, absent in S. cerevisiae; Hap43p-repressed
CR_02910W_A	orf19.2848	ATG13	1.767203669	0.821468319	9.14166E-11	Predicted regulatory subunit of the Atg1 signaling complex; required for vesicle formation during autophagy, biofilm formation, and the cytoplasm-to-vacuole targeting (Cvt) pathway; Spider biofilm induced
C4_05250W_A	orf19.6864	orf19.6864	1.76702347	0.821321203	0.014512784	Putative ubiquitin-protein ligase; role in protein ubiquitination; Spider biofilm induced
C5_03610W_A	orf19.6673	HEX1	1.76474034	0.819455924	1.40914E-12	Beta-N-acetylhexosaminidase/chitobiase, highly glycosylated enzyme that is secreted to the periplasm and culture medium; required for full virulence; may have role in carbon or nitrogen scavenging; possibly an essential gene (UAU1 method)
C1_13480W_A	orf19.4980	HSP70	1.759508164	0.815172208	0.035318872	Putative hsp70 chaperone; role in entry into host cells; heat-shock, amphotericin B, cadmium, ketoconazole-

						induced; surface localized in yeast and hyphae; antigenic in host; farnesol-downregulated in biofilm; Spider biofilm induced
CR_04770C_A	orf19.6315	orf19.6315	1.75722542	0.813299275	0.042324954	Ortholog of *C. dubliniensis* CD36: Cd36_30140, *C. parapsilosis* CDC317: CPAR2_204040, *C. auris* B8441: B9J08_001978 and *Candida tenuis* NRRL Y-1498: CANTEDRAFT_114703
C1_10290W_A	orf19.4899	GCA1	1.753890362	0.810558566	8.95521E-06	Extracellular/plasma membrane-associated glucoamylase; expressed in rat oral infection; regulated by carbohydrates, pH, galactose; promotes biofilm matrix formation; flow model biofilm induced; Bcr1 repressed in RPMI a/a biofilms
C5_03620W_A	orf19.6674	BTS1	1.7531585	0.809956434	8.72946E-08	Putative geranylgeranyl diphosphate synthase; repressed by benomyl treatment; Spider biofilm induced
C2_06010W_A	orf19.4119	SPO72	1.752311353	0.809259137	7.86703E-14	Protein described as similar to S. cerevisiae sporulation protein; ortholog of S. cerevisiae Atg2, an autophagic vesicle formation protein; up-regulation associated with azole resistance; Spider biofilm induced
C2_04190C_A	orf19.802	UGA1	1.747019921	0.804896059	7.85679E-13	Putative GABA transaminase; transcription regulated by Mig1 and Tup1; stationary phase enriched protein; rat catheter and Spider biofilm induced
CR_05430W_A	orf19.3512	CSP1	1.7443453	0.802685656	2.40801E-05	Putative cell wall associated protein; gene only found in C. albicans and C. dubliniensis; highly upregulated during chlamydospore development in both species; localized to chlamydospore cell wall
C1_03720C_A	orf19.1037	orf19.1037	1.744206208	0.802570612	2.97908E-17	Protein of unknown function; rat catheter biofilm repressed

C1_00410C_A	orf19.6066	orf19.6066	1.743576838	0.802049944	5.16968E-10	Hexadecenal dehydrogenase; involved in the conversion of sphingosine 1-phosphate breakdown product hexadecenal to hexadecenoic acid; Spider biofilm induced
C7_01700W_A	orf19.6554	orf19.6554	1.743264214	0.801791245	1.80037E-07	Regulator of calcineurin; regulated by calcineurin-Crz1 pathway; feedback regulator of calcineurin-dependent signaling; Hap43-repressed; induced by ketoconazole, hypoxia, during growth in the mouse cecum; Spider biofilm induced
C1_05820C_A	orf19.2469	RAD10	1.7391318	0.798367271	2.94866E-06	Ortholog of S. cerevisiae Rad10, an endonuclease involved in nucleotide excision repair; mutant is extremely sensitive to UV irradiation; transcript repressed in alkaline conditions
C6_00120W_A	orf19.6325.1	orf19.6325.1	1.734468328	0.794493497	0.040543966	Ortholog of *S. cerevisiae*: MRX7, *C. glabrata* CBS138: CAGL0K04785g, *C. dubliniensis* CD36: Cd36_19820, *C. parapsilosis* CDC317: CPAR2_603375 and *C. auris* B8441: B9J08_005197
CR_01530C_A	orf19.2539	orf19.2539	1.733967538	0.79407689	0.015740061	Protein of unknown function; transcript detected on high-resolution tiling arrays
C7_03420C_A	orf19.1333	SNG3	1.729399324	0.79027103	5.33373E-09	Putative membrane transporter; Hap43p-induced gene; mutation confers hypersensitivity to toxic ergosterol analog; shows colony morphology-related gene regulation by Ssn6p
CR_02350C_A	orf19.3734	GEF2	1.728123308	0.789206163	8.54853E-14	Member of the voltage chloride channel family; Hap43p-repressed gene
CR_01900C_A	orf19.2587	BIO5	1.72587228	0.787325705	7.41528E-05	Putative transporter; Hap43, flucytosine repressed; possibly essential, disruptants not obtained by UAU1 method; Spider biofilm induced; transcription regulated by biotin and Vhr1p

C3_01310W_A	orf19.1720	orf19.1720	1.725696289	0.787178582	2.79954E-10	Ortholog(s) have role in mitotic recombination
C2_06460W_A	orf19.23	RTA3	1.719731872	0.782183648	1.60334E-13	7-transmembrane receptor protein involved in regulation of asymmetric lipid distribution in plasma membrane; involved in biofilm formation; putative drug-responsive regulatory site; rat catheter biofilm induced
C7_02010C_A	orf19.6518	orf19.6518	1.718896413	0.781482605	0.002123637	Predicted aldehyde dehydrogenase [NAD(P)+]; Spider biofilm induced
C7_03030W_A	orf19.5158	orf19.5158	1.71691378	0.779817592	0.000178583	Protein with similarity to a human gene associated with colon cancer and to orf19.5158; regulated by Gcn4, Cyr1; induced by amino acid starvation; macrophage-induced protein, macrophage-repressed; Spider biofilm induced
C7_01940C_A	orf19.6527	orf19.6527	1.715818514	0.778896964	1.46511E-16	Pheromone-regulated protein (Prm10) of S. cerevisiae; colony morphology-related gene regulation by Ssn6; induced by Mnl1 under weak acid stress; possibly essential gene, disruptants not obtained by UAU1 method; Spider biofilm induced
CR_07190W_A	orf19.730	orf19.730	1.711487181	0.775250487	3.60552E-18	Ortholog(s) have GTPase activator activity and role in establishment or maintenance of actin cytoskeleton polarity, small GTPase mediated signal transduction
C6_03260W_A	orf19.5614	orf19.5614	1.708328274	0.772585231	2.73613E-13	Putative ribonuclease H1; possibly an essential gene, disruptants not obtained by UAU1 method; flow model biofilm induced; Spider biofilm induced
C5_05060C_A	orf19.3998	orf19.3998	1.704450008	0.769306285	0.000527438	Ortholog(s) have Atg8 ligase activity, protein-macromolecule adaptor activity
C6_04410C_A	orf19.2124	orf19.2124	1.703009814	0.768086749	0.000161588	Predicted alcohol dehydrogenase; Spider biofilm induced
C3_02650W_A	orf19.257	orf19.257	1.701926016	0.767168323	0.045291396	Transcription is negatively regulated by Sfu1p; repressed by nitric oxide

C1_11670W_A	orf19.1152	orf19.1152	1.701690741	0.766968871	1.77918E-05	Protein of unknown function; induced in core stress response; Gcn2 and Gcn4 regulated; flow model biofilm induced; Spider biofilm induced
C3_07670W_A	orf19.6720	orf19.6720	1.699373241	0.765002753	1.02602E-05	P-Loop domain-containing protein of unknown function; transposon mutation affects filamentous growth; Spider biofilm induced
C4_00860C_A	orf19.4706	orf19.4706	1.697893206	0.763745719	7.25745E-05	Protein of unknown function; induced in cyr1 or ras1 mutant; induced by fluconazole, by alpha pheromone in SpiderM medium and during oralpharyngeal candidasis; Spider biofilm induced
C1_04700C_A	orf19.775	orf19.775	1.696674879	0.762710138	2.12336E-06	Ortholog of *C. dubliniensis* CD36: Cd36_04450, *C. parapsilosis* CDC317: CPAR2_105460, *C. auris* B8441: B9J08_002463, *Debaryomyces hansenii* CBS767: DEHA2D07128g and *Pichia stipitis* Pignal: PICST_80203
C3_04130W_A	orf19.5857	orf19.5857	1.693211437	0.759762139	5.80174E-13	Ortholog of *C. dubliniensis* CD36: Cd36_84070, *C. parapsilosis* CDC317: CPAR2_102720, *Candida tenuis* NRRL Y-1498: CANTEDRAFT_98617 and *Debaryomyces hansenii* CBS767: DEHA2D07392g
C1_06610C_A	orf19.6249	HAK1	1.690264091	0.757248674	0.000683361	Putative potassium transporter; similar to Schwanniomyces occidentalis Hak1p; amphotericin B induced; induced upon phagocytosis by macrophage; Hap43-repressed; rat catheter biofilm repressed
C1_02390W_A	orf19.2926	orf19.2926	1.690036235	0.757054179	4.83293E-06	Putative nuclease required for DNA single- and double-strand break repair; rat catheter biofilm induced
C7_03470W_A	orf19.6704	orf19.6704	1.689335818	0.756456145	0.01408817	Protein of unknown function; Hap43-repressed gene
C6_00540W_A	orf19.4206	orf19.4206	1.688301434	0.75557251	1.89167E-06	Ortholog(s) have crossover junction endodeoxyribonuclease activity, endodeoxyribonuclease activity, enzyme inhibitor activity

C1_04750W_A	orf19.769	IFE1	1.687595024	0.754968739	0.000209024	Putative medium-chain alcohol dehydrogenase; rat catheter and Spider biofilm repressed
C1_11610C_A	orf19.1159	orf19.1159	1.686019612	0.753621318	4.4807E-06	Protein similar to A. nidulans CysA serine O-trans-acetylase; suggests that C. albicans uses an O-acetyl-serine (OAS) pathway of sulfur assimilation; F-12/CO2 early biofilm induced; Spider biofilm induced
C1_00630W_A	orf19.6043	orf19.6043	1.683521159	0.751481854	2.1185E-10	Has domain(s) with predicted UDP-N-acetylmuramate dehydrogenase activity, catalytic activity, flavin adenine dinucleotide binding activity
C2_02730W_A	orf19.5833	ECS3	1.679801734	0.748290962	6.72939E-10	Protein involved in resistance to caspofungin and anidulafungin
CR_05360C_A	orf19.5287	orf19.5287	1.677771303	0.746546076	0.000231349	Ortholog of *C. dubliniensis* CD36: Cd36_30690, *C. parapsilosis* CDC317: CPAR2_204140, *Debaryomyces hansenii* CBS767: DEHA2F16940g and *Candida tropicalis* MYA-340: CTRG_00570
C1_02130C_A	orf19.3670	GAL1	1.676117574	0.745123353	9.87804E-12	Galactokinase; galactose, Mig1, Tup1, Hap43 regulated; fluconazole, ketoconazole-induced; stationary phase enriched protein; GlcNAc-induced protein; farnesol, hypoxia-repressed in biofilm; rat catheter and Spider biofilm induced
C4_02300W_A	orf19.2772	HOS3	1.675694699	0.744759323	2.03472E-11	Histone deacetylase; similar to S. cerevisiae Hos3p; greater expression and longer mRNA in white cells, compared to opaque cells; has conserved deacetylation motif
C6_01770W_A	orf19.3407	RAD18	1.671805652	0.741407144	1.7522E-07	Putative transcription factor with zinc finger DNA-binding motif; Hap43p-repressed gene
C4_07030W_A	orf19.3106	MET16	1.668097595	0.738203698	8.15397E-07	Putative 3'-phosphoadenylsulfate reductase; sulfur amino acid metabolism; reports differ on regulation during biofilm formation; fungal-specific; possibly an essential gene,

						disruptants not obtained by UAU1 method; Hap43p-repressed gene
C5_00740W_A	orf19.576	CTF8	1.667556199	0.737735383	3.45291E-05	Putative kinetochore protein with a predicted role in sister chromatid cohesion; repressed during the mating process; flow model biofilm induced
C4_02390W_A	orf19.2765	PGA62	1.667355282	0.737561548	1.1366E-06	Adhesin-like cell wall protein; putative GPI-anchor; fluconazole-induced; induced in high iron; induced during cell wall regeneration; Cyr1 or Ras1 repressed; Tbf1 induced
C1_07850C_A	orf19.5056	orf19.5056	1.666770401	0.737055386	8.39928E-05	Ortholog(s) have enzyme regulator activity, ubiquitin protein ligase activity and role in anaphase-promoting complex-dependent catabolic process, chromatin assembly, positive regulation of ubiquitin protein ligase activity
C3_05600W_A	orf19.6995	ATO6	1.666667227	0.736966079	0.003765548	Putative fungal-specific transmembrane protein
C5_05450C_A	orf19.4044	MUM2	1.665592822	0.736035756	0.000976311	Protein similar to S. cerevisiae Mum2, a protein essential for meiotic DNA replication and sporulation; induced by alpha pheromone in SpiderM medium; transcript regulated by Tup1
C3_04840C_A	orf19.5952	orf19.5952	1.664979911	0.735504771	5.05276E-07	Protein of unknown function; induced by nitric oxide independent of Yhb1; Sef1, Sfu1, and Hap43-induced; rat catheter and Spider biofilm induced
C1_07770W_A	orf19.4712	FGR6	1.661996153	0.732917043	7.58041E-05	Protein lacking an ortholog in S. cerevisiae; member of a family encoded by FGR6-related genes in the RB2 repeat sequence; transposon mutation affects filamentous growth
C1_14590C_A	orf19.7276.1	TLO4	1.661413562	0.732411236	0.004031274	Member of a family of telomere-proximal genes of unknown function; transcript induced in an RHE model of oral candidiasis; Hap43-repressed

C2_07400C_A	orf19.1891	APR1	1.661164988	0.73219537	8.20067E-14	Vacuolar aspartic proteinase; transcript equivalent in yeast-form and mycelial cells but is elevated at lower growth temperatures; upregulated iby human neutrophils; protein enriched in stationary phase; Spider biofilm induced
C4_02200C_A	orf19.4570	orf19.4570	1.659315448	0.730588179	3.09956E-09	Ortholog of *C. dubliniensis* CD36: Cd36_42090, *Debaryomyces hansenii* CBS767: DEHA2C14872g, Pichia stipitis Pignal: PICST_74821 and *Candida tropicalis* NEW ASSEMBLY: CTRG1_00174
C2_02590W_A	orf19.1585	ZRT2	1.659177841	0.730468532	4.90917E-05	Zinc transporter, essential for zinc uptake and acidic conditions tolerance; transcript induced by amphotericin B, interaction with macrophages; induced in oralpharyngeal candidiasis; Spider biofilm induced
C1_14170W_A	orf19.7224	orf19.7224	1.657769402	0.72924334	6.62934E-11	Ortholog(s) have ubiquitin protein ligase activity and role in histone catabolic process, histone ubiquitination
C2_00460W_A	orf19.2082	SAP30	1.655662864	0.727408933	0.001619563	Aspartic-type endopeptidase; involved in degradation of alpha pheromone; functional equivalent of S. cerevisiae Bar1; a-cell specific; induced by alpha pheromone
C4_03020W_A	orf19.2690	MGM1	1.654587796	0.726471846	2.0732E-14	Putative mitochondrial GTPase; required for mitochondrial morphology and genome maintenance; Spider biofilm induced
CR_03800C_A	orf19.4378	PPH3	1.653846625	0.725825448	2.75459E-10	Putative catalytic subunit of protein phosphatase complex; functions with regulatory subunit Psy2p in dephosphorylation of Rad53p in response to DNA damage; dephosphorylates Rfa2p in G1 phase; ortholog of *S. cerevisiae* Pph3p
C4_03990C_A	orf19.1208	orf19.1208	1.652587196	0.724726395	0.01316838	Predicted ORF in the Major Repeat Sequence on chromosome 4; member of a family encoded by FGR6-related genes in the RB2 repeat sequence

C2_07120W_A	orf19.2267	RFA2	1.652496841	0.724647514	6.78663E-10	Putative DNA replication factor A; RNA abundance regulated by cell cycle, tyrosol and cell density
C2_08090W_A	orf19.2177	orf19.2177	1.651078046	0.723408318	0.021352355	Sef1p-, Sfu1p-, and Hap43p-regulated gene; overlaps IFM3/orf19.2176
C4_01790W_A	orf19.4614	ATG11	1.649389756	0.721932352	1.38463E-12	Adapter protein for pexophagy and the cytoplasm-to-vacuole targeting (Cvt) pathway; Spider biofilm induced
C4_03710C_A	orf19.1307	orf19.1307	1.647686864	0.72044209	4.19129E-05	Predicted membrane protein; rat catheter biofilm induced
C7_02810W_A	orf19.5180	PRX1	1.647647352	0.720407494	1.39599E-10	Thioredoxin peroxidase; transcriptionally induced by interaction with macrophage; fluconazole induced; Fkh2p-downregulated; caspofungin repressed; protein present in exponential and stationary growth phase yeast cultures
C5_00100C_A	orf19.5686	orf19.5686	1.646957801	0.71980359	4.22547E-05	Protein of unknown function; Spider biofilm induced
C4_01550C_A	orf19.4630	CPA1	1.646131216	0.71907934	0.005074112	Putative carbamoyl-phosphate synthase subunit; alkaline repressed; rat catheter, Spider and flow model biofilm induced
C4_03970W_A	orf19.1207	orf19.1207	1.645964877	0.718933551	2.73699E-05	Ortholog of *C. dubliniensis* CD36: Cd36_33200 and *Candida albicans* WO-1: CAWG_03416
C7_03780C_A	orf19.7204	orf19.7204	1.645502026	0.718527803	0.00308539	Has domain(s) with predicted catalytic activity, nitronate monooxygenase activity
C7_02790C_A	orf19.5182	POL3	1.644523397	0.717669534	1.83797E-12	Large subunit of DNA polymerase III; partially complements defects of an S. cerevisiae cdc2 mutant; differing reports about periodic (G1/S) or non-periodic mRNA expression through cell cycle; Hap43p-repressed
C6_01420C_A	orf19.3442	orf19.3442	1.643213569	0.71652	4.67442E-07	Putative oxidoreductase; Hap43-repressed gene
C7_00170W_A	orf19.7106	VPS70	1.642345367	0.715757541	2.76305E-11	Has domain(s) with predicted peptidase activity and role in proteolysis
C2_09590C_A	orf19.1395	orf19.1395	1.641504402	0.715018619	6.42541E-06	Ortholog(s) have copper ion transmembrane transporter activity, inorganic phosphate transmembrane transporter

						activity and role in cellular copper ion homeostasis, copper ion transmembrane transport, phosphate ion transmembrane transport
C1_09250W_A	orf19.4784	CRP1	1.641050604	0.714619727	0.000219965	Copper transporter; CPx P1-type ATPase; mediates Cu resistance; similar to Menkes and Wilson disease proteins; copper-induced; Tbf1-activated; suppresses Cu sensitivity of S. cerevisiae cup1 mutant; flow model biofilm induced
C3_04920C_A	orf19.5961	orf19.5961	1.640982928	0.714560229	5.91644E-13	Ortholog(s) have proteasome regulatory particle binding activity, role in proteasome regulatory particle assembly and cytosol, nucleus localization
C6_00850W_A	orf19.86	orf19.86	1.640953244	0.714534132	5.6589E-05	Putative glutathione peroxidase; induced by peroxide, exposure to neutrophils and macrophage blood fractions; repressed during infection of macrophages; Spider biofilm induced; flow model biofilm repressed
C3_00540C_A	orf19.5409	IST1	1.64076605	0.714369546	3.00107E-10	Protein with a positive role in the multivesicular body sorting pathway; rat catheter biofilm repressed
C4_05070C_A	orf19.3770	ARG8	1.640428781	0.714072961	1.37794E-06	Putative acetylornithine aminotransferase; Gcn2, Gcn4 regulated; rat catheter biofilm induced; Spider biofilm induced
C1_03840W_A	orf19.1026.1	orf19.1026.1	1.639067737	0.712875477	0.005640541	Ortholog of *C. dubliniensis* CD36: Cd36_03580, *C. parapsilosis* CDC317: CPAR2_105090, *C. auris* B8441: B9J08_004984 and *Candida tenuis* NRRL Y-1498: CANTEDRAFT_106351
C6_02890C_A	orf19.5565	HPD1	1.638192698	0.712105068	0.000505303	3-hydroxypropionate dehydrogenase; involved in degradation of toxic propionyl-CoA; rat catheter and Spider biofilm induced
C2_05570C_A	orf19.6869	orf19.6869	1.636844838	0.710917571	3.94335E-07	Putative lipid raft associated protein; Spider biofilm induced

C7_03490W_A	orf19.6706	GYP7	1.635911243	0.710094476	4.82545E-12	Protein similar to S. cerevisiae Gyp7p (GTPase-activating protein for Ypt1p); caspofungin-induced
C3_07660W_A	orf19.6722	RAD4	1.635297768	0.709553357	6.68309E-11	Protein similar to S. cerevisiae Rad4p; down-regulation associated with azole resistance
C4_05880W_A	orf19.1275	GAT1	1.63146395	0.706167109	0.000108011	GATA-type transcription factor; regulator of nitrogen utilization; required for nitrogen catabolite repression and utilization of isoleucine, tyrosine and tryptophan N sources; required for virulence in a mouse systemic infection model
CR_06290C_A	orf19.3881	orf19.3881	1.630616018	0.705417092	1.95657E-10	Ortholog of *C. dubliniensis* CD36: Cd36_31790, *C. parapsilosis* CDC317: CPAR2_204900, *C. auris* B8441: B9J08_000806 and *Candida tenuis* NRRL Y-1498: CANTEDRAFT_136864
C3_01040C_A	orf19.2508	PRM9	1.628593599	0.703626637	0.012657285	Protein described a similar to S. cerevisiae Prm9; not the ortholog though; mutant is viable
C4_00110C_A	orf19.5634	FRP1	1.627464931	0.702626456	0.004415685	Ferric reductase-related protein involved in heme acquisition; alkaline-induced by Rim101; iron-chelation-induced by CCAAT-binding factor; fluconazole-repressed; ciclopirox-, hypoxia-, Hap43-induced
C5_03490C_A	orf19.6658	orf19.6658	1.626668111	0.701919928	2.89479E-10	Stationary phase enriched protein; predicted ORF from Assembly 19; removed from Assembly 20; subsequently reinstated in Assembly 21 based on comparative genome analysis
C4_06390W_A	orf19.2896	SOU1	1.625357898	0.70075743	4.64448E-06	Enzyme involved in utilization of L-sorbose; has sorbitol dehydrogenase, fructose reductase, and sorbose reductase activities; NAD-binding site motif; transcriptional regulation affected by chromosome 5 copy number; Hap43p-induced gene

C1_11200W_A	orf19.2296	orf19.2296	1.625128549	0.700553841	3.79028E-06	Predicted mucin-like protein; ketoconazole-induced; fluconazole-repressed; induced in cyr1 mutant; colony morphology-related gene regulation by Ssn6; flow model biofilm induced; Spider biofilm induced
CR_07910C_A	orf19.607	orf19.607	1.625049116	0.700483323	2.24819E-08	Protein involved in transcription-coupled nucleotide excision repair of UV-induced DNA lesions; Spider biofilm induced
CR_07560W_A	orf19.729.1	RGD3	1.621898699	0.697683714	1.77758E-12	Putative Rho GTPase activating protein; fungal-specific (no human or murine homolog)
CR_01090W_A	orf19.3245	orf19.3245	1.621724634	0.697528874	7.83565E-07	Ortholog of C. dubliniensis CD36: Cd36_26090, C. parapsilosis CDC317: CPAR2_801510, C. auris B8441: B9J08_002641 and Candida tenuis NRRL Y-1498: CANTEDRAFT_137093
C5_03240W_A	orf19.2655	BUB3	1.620845059	0.696746186	5.97914E-07	Protein similar to S. cerevisiae Bub3; a kinetochore checkpoint component; induced by hydroxyurea treatment; flow model biofilm induced; Spider biofilm induced
C1_10740C_A	orf19.2344	ASR1	1.620409179	0.696358163	0.001209707	Heat shock protein; transcript regulated by cAMP, osmotic stress, ciclopirox olamine, ketoconazole; repressed by Cyr1, Ras1; colony morphology-related regulated by Ssn6; stationary phase enriched; Hap43-induced; Spider biofilm induced
C2_02180W_A	orf19.1534	orf19.1534	1.618997477	0.695100737	1.05129E-05	Ortholog of S. cerevisiae Zrt3, vacuolar membrane zinc transporter; predicted Kex2 substrate; induced in oralpharyngeal candidasis; flow model biofilm induced; Spider biofilm induced
C2_07790C_A	orf19.2202	orf19.2202	1.618947497	0.695056199	0.010894659	Protein of unknown function; induced by alpha pheromone in SpiderM medium

C1_06970C_A	orf19.6211	orf19.6211	1.618704255	0.694839422	5.73327E-08	Protein of unknown function; protein newly produced during adaptation to the serum
C2_10420W_A	orf19.3490	FGR6	1.618068434	0.694272626	1.77498E-05	Protein lacking an ortholog in S. cerevisiae; member of a family encoded by FGR6-related genes in the RB2 repeat sequence; transposon mutation affects filamentous growth
C1_03980W_A	orf19.4473	SPC19	1.615910391	0.692347196	0.044542234	Essential subunit of the Dam1 (DASH) complex, which acts in chromosome segregation by coupling kinetochores to spindle microtubules
C2_01990C_A	orf19.1513	FAB1	1.615626359	0.692093589	1.66453E-11	Phosphatidylinositol 3-phosphate 5-kinase; required for hyphal growth on solid media, and for wild-type vacuolar morphology and acidification; not required for wild-type virulence in mouse systemic infection or for adherence to HeLa cells
C1_12890W_A	orf19.4919	orf19.4919	1.613259174	0.68997823	0.008276719	Has domain(s) with predicted nucleic acid binding activity
C5_05260W_A	orf19.4019	PSY4	1.608778787	0.685965964	3.74675E-05	Regulatory subunit of protein phosphatase PP4; required for recovery from filamentation induced by DNA damage; mutants show increased virulence
C7_02020W_A	orf19.6517	RAD14	1.608555614	0.685765816	1.22085E-05	Putative DNA repair protein; transcription is regulated upon yeast-hyphal switch; flucytosine repressed
C1_09220W_A	orf19.4780	orf19.4780	1.608198541	0.685445526	1.04918E-05	Predicted MFS family membrane transporter, member of the drug:proton antiporter (12 spanner) (DHA1) family; Spider biofilm induced
C3_00480C_A	orf19.5417	DOT5	1.607809193	0.685096205	1.36714E-06	Putative nuclear thiol peroxidase; alkaline downregulated; sumoylation target; Spider and flow model biofilm induced
C3_03530W_A	orf19.351	orf19.351	1.607162218	0.684515554	0.000280667	Ortholog of S. cerevisiae: MAY24, C. glabrata CBS138: CAGL0L08382g, C. dubliniensis CD36: Cd36_83540, C. parapsilosis CDC317: CPAR2_404520 and C. auris B8441: B9J08_002269

C1_01810C_A	orf19.4543	UGA2	1.605982003	0.683455726	1.70464E-09	Predicted succinate semialdehyde dehydrogenase; predicted role in glutamate catabolism; transcription regulated by Mig1, Tup1, Gcn4; mutants are viable
C6_04190C_A	orf19.1075	orf19.1075	1.60531553	0.682856892	0.003250326	Protein of unknown function; Spider biofilm induced
C2_09470C_A	orf19.1605	PMS1	1.604371594	0.682008328	4.98336E-09	Putative DNA mismatch repair factor; ortholog of S. cerevisiae PMS1 which is an ATP-binding protein involved in DNA mismatch repair
C6_00320C_A	orf19.1192	DNA2	1.603905376	0.681589031	2.76315E-09	Protein similar to S. cerevisiae Dna2p, which is a DNA replication factor involved in DNA repair; induced under hydroxyurea treatment
C4_02250C_A	orf19.4565	BGL2	1.603490364	0.681215684	0.00015072	Cell wall 1, 3-beta-glucosyltransferase; mutant has cell-wall and growth defects, but wild-type 1, 3- or 1, 6-beta-glucan content; antigenic; virulence role in mouse systemic infection; rat catheter biofilm induced
C4_06600W_A	orf19.2873	TOP2	1.602437902	0.680268451	7.54492E-12	DNA topoisomerase II; catalyzes ATP-dependent DNA relaxation and decatenation in vitro; Y842 predicted to be catalytic; functional homolog of S. cerevisiae Top2p; sensitive to amsacrine or doxorubicin; farnesol-upregulated in biofilm
C7_00580C_A	orf19.7061	orf19.7061	1.602436238	0.680266952	1.92321E-06	Ortholog(s) have ATPase activity
C2_09280C_A	orf19.4090	orf19.4090	1.601678354	0.679584458	1.03848E-07	Predicted membrane transporter, member of the fucose:proton symporter (FHS) family, major facilitator superfamily (MFS)
C2_07470W_A	orf19.1883	YCS4	1.600899946	0.678883144	3.17943E-09	Putative condensin complex subunit; cell-cycle regulated periodic mRNA expression
C4_04450C_A	orf19.3841	ATG1	1.600208013	0.678259455	9.85368E-07	Putative protein serine/threonine kinase; predicted role in vesicle formation in autophagy and the cytoplasm-to-vacuole targeting (Cvt) pathway; Spider biofilm induced

C2_00740C_A	orf19.2050	orf19.2050	1.599755867	0.677851757	1.40617E-08	Ortholog(s) have sterol esterase activity, role in cellular lipid metabolic process, sterol metabolic process and integral component of membrane, lipid droplet localization
C2_08560W_A	orf19.3623	SMC2	1.59829514	0.67653384	1.32873E-07	Protein similar to S. cerevisiae Smc2p, which is a component of the condensin complex involved in mitotic chromosome condensation; induced under hydroxyurea treatment
C1_00750C_A	orf19.6031	VPS27	1.595761136	0.674244716	1.30451E-07	Putative ESCRT-0 complex protein with a role in multivesicular body (MVB) trafficking
C6_02680W_A	orf19.5539	orf19.5539	1.595611501	0.674109427	4.21536E-06	Ortholog(s) have SNAP receptor activity, role in retrograde vesicle-mediated transport, Golgi to endoplasmic reticulum and SNARE complex, integral component of cytoplasmic side of endoplasmic reticulum membrane localization
CR_02070C_A	orf19.2608	ADH5	1.593905417	0.672566022	0.000551425	Putative alcohol dehydrogenase; regulated by white-opaque switch; fluconazole-induced; antigenic in murine infection; regulated by Nrg1, Tup1; Hap43, macrophage repressed, flow model biofilm induced; Spider biofilm induced
C2_00540W_A	orf19.2073	orf19.2073	1.593338755	0.672053027	3.28383E-07	Protein with a multidrug and toxin extrusion protein domain; induced by Mnl1 under weak acid stress
C2_00550W_A	orf19.2072	orf19.2072	1.592124979	0.670953589	0.011157885	Ortholog(s) have (R)-carnitine transmembrane transporter activity, choline transmembrane transporter activity, ethanolamine transmembrane transporter activity
C1_01620C_A	orf19.3352	orf19.3352	1.590491205	0.669472394	0.000190205	Has domain(s) with predicted oxidoreductase activity and role in metabolic process
CR_01810C_A	orf19.2581	orf19.2581	1.590269833	0.669271579	0.001447564	Protein with a predicted epimerase/dehydratase domain; Hap43-repressed gene
CR_07100W_A	orf19.1813	FLC2	1.589789485	0.668835741	5.71706E-07	Protein involved in heme uptake; putative FAD transporter, similar to S. cerevisiae Flc2p

C3_04160W_A	orf19.5859	DAL8	1.589374093	0.668458734	0.000451294	Putative allantoate permease; fungal-specific (no human or murine homolog)
C3_02790W_A	orf19.270	orf19.270	1.58779611	0.667025667	0.014653184	Ortholog of C. dubliniensis CD36: Cd36_82780, C. parapsilosis CDC317: CPAR2_102150, Pichia stipitis Pignal: psti_CGOB_00155 and Candida tropicalis MYA-3404: CTRG_02557
C5_03780C_A	orf19.1116	orf19.1116	1.587625984	0.66687108	0.00191446	Protein of unknown function; planktonic growth-induced gene
C4_07010C_A	orf19.3108	orf19.3108	1.586916702	0.666226402	3.15771E-06	Putative DNA repair methyltransferase; induced by nitric oxide independent of Yhb1; Spider biofilm induced
C6_04120C_A	orf19.4557	orf19.4557	1.586689302	0.666019654	1.07765E-06	Ortholog(s) have microtubule binding activity and role in mitotic spindle assembly checkpoint signaling, protein localization to kinetochore, sister chromatid biorientation
C3_06810W_A	orf19.6821	orf19.6821	1.586075618	0.665461554	5.41564E-09	Ortholog(s) have ubiquitin protein ligase activity
C1_12880C_A	orf19.4918	orf19.4918	1.586058699	0.665446166	4.70948E-07	Has domain(s) with predicted DNA binding, nucleic acid binding activity
C2_00680C_A	orf19.2060	SOD5	1.585722359	0.665140195	0.007313851	Cu-containing superoxide dismutase; protects against oxidative stress; induced by neutrophils, hyphal growth, caspofungin, osmotic/oxidative stress; oralpharyngeal candidiasis induced; rat catheter and Spider biofilm induced
C4_04840C_A	orf19.3795	AGP3	1.58383698	0.66342385	5.74198E-07	Putative serine transporter; possible role in assimilation of sulfur; F-12/CO2 early biofilm induced
C1_02310C_A	orf19.3688	orf19.3688	1.583439995	0.663062197	0.007497589	Ortholog(s) have chromatin DNA binding activity and role in cell wall mannoprotein biosynthetic process, positive regulation of transcription by RNA polymerase II, telomere maintenance, telomere maintenance via recombination

C2_05690C_A	orf19.6881	YTH1	1.583411581	0.663036308	0.001265384	Putative mRNA cleavage and polyadenylation specificity factor; transcription is regulated upon yeast-hyphal switch; decreased expression in hyphae compared to yeast-form cells; fluconazole or flucytosine induced
C2_10870W_A	orf19.5370	orf19.5370	1.582377715	0.662094014	0.001397542	Ortholog(s) have fungal-type vacuole membrane localization
C7_00370W_A	orf19.7083	DCC1	1.582370747	0.66208766	0.000673696	Protein with a predicted role in sister chromatid cohesion and telomere length maintenance; cell-cycle regulated periodic mRNA expression
C1_11810W_A	orf19.1135	CAS1	1.582202885	0.661934608	9.92128E-09	Putative transcription factor with Ku70/Ku80 beta-barrel DNA-binding motif; involved in telomerase regulation and telomere protection; mutation causes marginal increase in caspofungin sensitivity
C1_09190C_A	orf19.4777	DAK2	1.581968956	0.661721289	0.047544821	Putative dihydroxyacetone kinase; repressed by yeast-hypha switch; fluconazole-induced; caspofungin repressed; protein enriched in stationary phase yeast cultures; flow model biofilm induced; rat catheter and Spider biofilm repressed
CR_04490C_A	orf19.540	orf19.540	1.579698888	0.659649587	6.73253E-05	Has domain(s) with predicted palmitoyl-(protein) hydrolase activity and role in cellular protein modification process
C7_03280C_A	orf19.5125	orf19.5125	1.579124217	0.659124661	1.49834E-06	Protein of unknown function; induced by ketoconazole; Spider, F-12/CO2 and flow model biofilm induced
C6_04490W_A	orf19.2133	LIP4	1.576496692	0.656722143	7.51825E-06	Secreted lipase, member of a differentially expressed lipase gene family with possible roles in nutrition and/or in creating an acidic microenvironment; expressed more strongly during mucosal infections than during systemic infections

C1_09380W_A	orf19.4800	RIM20	1.574886346	0.655247718	1.61354E-06	Protein involved in the pH response pathway; binds to the transcription factor Rim101 and may serve as a scaffold to facilitate the C-terminal proteolytic cleavage that activates Rim101; required for alkaline pH-induced hyphal growth
C1_10410W_A	orf19.4911	orf19.4911	1.574068336	0.654498175	8.10572E-07	BED zinc finger protein; predicted DNA binding protein; Spider biofilm repressed
C3_05220W_A	orf19.6000	CDR1	1.57216166	0.652749572	2.76315E-09	Multidrug transporter of ABC superfamily; transports phospholipids in an in-to-out direction; induced by beta-estradiol, progesterone, corticosteroid, or cholesterol; Spider biofilm induced
C5_05390C_A	orf19.4035	PGA4	1.57170452	0.652330017	2.98166E-05	GPI-anchored cell surface protein; beta-1, 3-glucanosyltransferase with similarity to the A. fumigatus GEL family; transcript induced in RHE model of oral candidiasis; fluconazol-induced
C1_13620W_A	orf19.4998	ROB1	1.571250547	0.651913246	8.652E-05	Zn(II)2Cys6 transcription factor; required for Spider model biofilm formation; mutant displays abnormal colony morphology and invasive growth; caspofungin repressed; flow model biofilm induced; rat catheter biofilm repressed
C6_04420W_A	orf19.2125	orf19.2125	1.568147682	0.649061433	2.09494E-07	Protein of unknown function; GlcNAc-induced protein; Spider biofilm induced; rat catheter biofilm repressed
C4_02070W_A	orf19.4584	PHO114	1.56648133	0.647527575	0.000177203	Acid phosphatase; induced by Mnl1 under weak acid stress; Spider biofilm induced
C1_08310W_A	orf19.5110	OPY2	1.566317673	0.647376843	6.55596E-10	Predicted transmembrane protein; role in cell wall biogenesis; required for Cek1 phosphorylation; Spider biofilm induced
C6_02180W_A	orf19.3458	orf19.3458	1.56619108	0.647260236	4.96035E-07	Ortholog(s) have role in late endosome to vacuole transport via multivesicular body sorting pathway and Vps55/Vps68 complex, fungal-type vacuole membrane localization

C6_01140C_A	orf19.121	ARC18	1.565246823	0.646390173	0.010957181	Putative ARP2/3 complex subunit; mutation confers hypersensitivity to cytochalasin D
C6_03990C_A	orf19.5775	orf19.5775	1.565085904	0.646241845	0.002012125	Predicted ORF overlapping the Major Repeat Sequence on chromosome 6; member of a family encoded by FGR6-related genes in the RB2 repeat sequence; rat catheter biofilm repressed
C6_03590C_A	orf19.5727	orf19.5727	1.564679184	0.645866883	9.57413E-06	Ortholog of C. dubliniensis CD36: Cd36_64130, C. parapsilosis CDC317: CPAR2_601230, C. auris B8441: B9J08_003898 and Candida tenuis NRRL Y-1498: CANTEDRAFT_115908
C4_03200C_A	orf19.2670	orf19.2670	1.56430579	0.645522557	4.55219E-06	Ortholog(s) have 3-hydroxyacyl-[acyl-carrier-protein] dehydratase activity and mitochondrion localization
C3_02820C_A	orf19.273	NSG2	1.5642797	0.645498496	0.005261655	Protein involved in regulation of C14-methylated sterol biosynthesis; mutations increase azole sensitivity
CR_00220W_A	orf19.7512	orf19.7512	1.564000298	0.645240788	2.73694E-08	Has domain(s) with predicted electron transfer activity, heme binding, iron ion binding, monooxygenase activity, oxidoreductase activity and acting on paired donors, more
C5_01310W_A	orf19.1938	orf19.1938	1.562433525	0.643794811	0.000330617	Ortholog of S.pombe SPCC825.05c; a predicted splicing coactivator; transcription repressed in azole-resistant strain overexpressing CDR1 and CDR2; induced by benomyl treatment
C2_09480W_A	orf19.1406	orf19.1406	1.562070462	0.643459532	0.000232361	Ortholog(s) have DNA-directed DNA polymerase activity, role in error-free translesion synthesis, error-prone translesion synthesis and mitochondrion, zeta DNA polymerase complex localization
C4_00950C_A	orf19.4699	orf19.4699	1.561752993	0.643166295	0.000972847	Putative phospholipase of patatin family; similar to S. cerevisiae Tgl3p; predicted Kex2p substrate

C4_04000W_A	orf19.5312	MET4	1.557563854	0.639291309	0.039608678	Putative transcription coactivator; predicted role in sulfur amino acid metabolism; required for yeast cell adherence to silicone substrate; Spider biofilm induced
C3_00210C_A	orf19.5449	orf19.5449	1.555967337	0.637811775	9.29499E-05	Predicted integral membrane protein; Spider biofilm induced
C2_08420W_A	orf19.3639	orf19.3639	1.551197171	0.633382077	2.66455E-05	Ortholog(s) have DNA-3-methyladenine glycosylase activity, alkylbase DNA N-glycosylase activity, damaged DNA binding activity
C3_02550C_A	orf19.244	DCG1	1.55044056	0.632678218	4.8987E-08	Protein of unknown function; ortholog of S. cerevisiae Dcg1; transcript regulated by Nrg1 and Mig1
CR_04350C_A	orf19.524	orf19.524	1.550022579	0.632289231	4.8303E-08	Has domain(s) with predicted ubiquitin-protein transferase activity, zinc ion binding activity
C1_04560W_A	orf19.6850	orf19.6850	1.548454528	0.630829018	7.99061E-06	Putative transcription factor with C3HC4 zinc finger DNA-binding motif; mutants are viable
CR_08060C_A	orf19.589	VPS21	1.547843957	0.630260036	1.3658E-07	Late endosomal Rab small monomeric GTPase involved in transport of endocytosed proteins to the vacuole; involved in filamentous growth and virulence; Spider biofilm induced
C3_03710W_A	orf19.6948	CCC1	1.546558764	0.629061652	1.96351E-08	Manganese transporter; required for normal filamentous growth; mRNA binds She3, localized to hyphal tips; repressed by NO, alkaline pH; colony morphology-related regulation by Ssn6; regulated by Sef1, Sfu1, Hap43; Spider biofilm induced
C7_02370W_A	orf19.6474	orf19.6474	1.546129489	0.62866115	1.52389E-05	Protein with chitin synthesis regulation, resistance to Congo red domain; membrane-localized protein; Spider biofilm induced
C6_03780C_A	orf19.5752	orf19.5752	1.545820413	0.628372722	3.43459E-07	Ortholog(s) have ubiquitin-protein transferase activity, role in SCF-dependent proteasomal ubiquitin-dependent protein

						catabolic process and SCF ubiquitin ligase complex localization
C3_02000W_A	orf19.1648	RAD50	1.545707565	0.628267399	3.51353E-08	Putative DNA double-strand break repair factor; involved in response to oxidative stress and drug resistance; flow model biofilm repressed
C1_02660C_A	orf19.2956	MGM101	1.544061248	0.626729981	0.00193195	Putative mitochondrial genome maintenance protein; fungal-specific (no human or murine homolog); mutation confers hypersensitivity to tubercidin (7-deazaadenosine)
C4_02600C_A	orf19.2739	CAC1	1.543935874	0.626612833	1.39555E-05	Putative component of the chromatin assembly factor I (CAF-1), which functions as a histone chaperone; involved in negative regulation of biofilm initiation; null mutant shows hyperfilamentous colony wrinkling
C1_00470C_A	orf19.6061	orf19.6061	1.54280541	0.62555611	2.77259E-06	Ortholog(s) have role in N-acylethanolamine metabolic process, N-acylphosphatidylethanolamine metabolic process and integral component of mitochondrial inner membrane localization
C1_14300C_A	orf19.7242	NCR1	1.542325433	0.625107208	8.49863E-07	Putative vacuolar membrane protein; predicted role in sphingolipid metabolism; transcript regulated by Nrg1 and Mig1; induced by prostaglandins
C1_09000W_A	orf19.4756	orf19.4756	1.54005533	0.622982184	9.10627E-05	Ortholog ofS. cerevisiae: YTP1, C. dubliniensis CD36: Cd36_08490, C. parapsilosis CDC317: CPAR2_801590, C. auris B8441: B9J08_004547 and Candida tenuis NRRL Y-1498: CANTEDRAFT_109732
C2_05640W_A	orf19.6874	orf19.6874	1.53876207	0.621770173	0.000306133	Putative helix-loop-helix (HLH) transcription factor with a role in filamentous growth
C2_01040W_A	orf19.2018.1	orf19.2018.1	1.53746168	0.620550453	0.001572988	PProtein of unknown function; gene has intron; similar to human BLOC1S2

CR_03690W_A	orf19.4368	orf19.4368	1.536781397	0.61991196	0.00014048	Has domain(s) with predicted hydrolase activity and role in cellular process
C3_02450W_A	orf19.233.1	HRT1	1.536459314	0.619609564	6.96377E-05	Ortholog of S. cerevisiae Hrt1; component of a nuclear ubiquitin-protein ligase complex involved in cell cycle control; induced by hydroxyurea; Spider biofilm induced
C7_03240W_A	orf19.5131	orf19.5131	1.535163275	0.618392104	3.59444E-08	Ortholog of S. cerevisiae Gid7, a GID complex protein; involved in proteasome-dependent catabolite inactivation of fructose-1, 6-bisphosphatase; Hap43-repressed gene
C1_02220C_A	orf19.3679	orf19.3679	1.535037274	0.618273688	8.65507E-05	Putative protein of unknown function; stationary phase enriched protein
C3_03690W_A	orf19.6950	orf19.6950	1.53492515	0.618168304	0.015620052	Putative vacuolar membrane transporter for cationic amino acids; Spider biofilm induced
C2_00510W_A	orf19.2076	orf19.2076	1.534335834	0.617614293	4.39973E-05	Protein of unknown function; S. pombe ortholog SPAC7D4.05 encodes a predicted hydrolase; Hap43-repressed; Spider biofilm induced
C2_04060C_A	orf19.814	SSY1	1.533762645	0.617075238	7.29613E-10	Amino acid sensor; required for wild-type hyphal growth on solid serum or Lees media, not under all conditions; 12 predicted membrane spanning regions; Hap43-repressed; Spider biofilm induced
C7_01100C_A	orf19.6896	orf19.6896	1.532670038	0.616047139	0.000523995	Predicted ORF overlapping the Major Repeat Sequence on chromosome 7; member of a family encoded by FGR6-related genes in the RB2 repeat sequence
C2_10440C_A	orf19.5318	RAD1	1.530657449	0.614151453	8.53622E-11	Putative single-stranded DNA endonuclease; transcript regulated by Nrg1; macrophage-induced gene
C7_02960C_A	orf19.5165	orf19.5165	1.530518685	0.614020658	2.56099E-07	Ortholog ofS. cerevisiae: YNR029C, C. glabrata CBS138: CAGL0M10747g, C. dubliniensis CD36: Cd36_72620, C. parapsilosis CDC317: CPAR2_704190 and C. auris B8441: B9J08_000738

C4_02470C_A	orf19.2755	PRE7	1.529482193	0.61304331	3.2974E-09	Subunit of the 20S core particle of the proteasome
CR_00880W_A	orf19.3266	orf19.3266	1.529442655	0.613006015	0.003674408	Ortholog of C. dubliniensis CD36: Cd36_25930, C. parapsilosis CDC317: CPAR2_800910, C. auris B8441: B9J08_003483 and Candida tenuis NRRL Y-1498: CANTEDRAFT_132053
CR_04120C_A	orf19.496	orf19.496	1.526802952	0.610513881	5.55933E-09	Ortholog(s) have ATP-dependent activity, acting on DNA, dinucleotide insertion or deletion binding, guanine/thymine mispair binding activity
CR_05490W_A	orf19.3519	SUA72	1.525250683	0.609046376	0.002004737	Predicted transcription factor; induced during planktonic growth, whereas related SUA71 is downregulated
CR_05710C_A	orf19.6641	orf19.6641	1.52446783	0.608305707	0.009105222	Ortholog of Candida albicans WO-1: CAWG_01892
C7_03020C_A	orf19.5159	DUG3	1.523246435	0.607149364	2.78775E-05	Putative glutamine amidotransferase (GATase II); role in glutathione catabolism;
C2_02360C_A	orf19.1557	orf19.1557	1.523169881	0.607076856	0.003398467	Ortholog(s) have S-adenosylmethionine-dependent methyltransferase activity and role in protein methylation
C2_03150C_A	orf19.5780	orf19.5780	1.521796521	0.605775469	1.32752E-07	Putative protein of unknown function; Hap43p-repressed gene
C2_08820C_A	orf19.3589	SPO11	1.519816565	0.603897207	0.030120736	DNA endonuclease; required for genetic recombination between homologous chromosomes during the parasexual cycle; produced in mitotically dividing cells; similar to S. cerevisiae Spo11 which functions in recombination during meiosis
C5_03670C_A	orf19.6679	orf19.6679	1.519155186	0.603269253	0.000203368	Has domain(s) with predicted metal ion binding activity
CR_05170C_A	orf19.638	FDH1	1.518668893	0.602807361	0.000225456	Formate dehydrogenase; oxidizes formate to CO2; Mig1 regulated; induced by macrophages; fluconazole-repressed; repressed by Efg1 in yeast, not hyphal conditions; stationary phase enriched; rat catheter and Spider biofilm induced

C1_05870W_A	orf19.2462	PRN3	1.518320207	0.602476081	0.04175608	Protein similar to pirin; induced by Mnl1 under weak acid stress; Hap43-repressed; Spider biofilm induced
C1_13630W_A	orf19.5000	CYB2	1.517839094	0.602018859	3.73737E-08	Putative cytochrome b2 precursor; induced in high iron; alkaline repressed; colony morphology-related gene regulation by Ssn6; Hap43-repressed; pider biofilm induced
CR_03220C_A	orf19.2401	orf19.2401	1.516873062	0.60110036	3.12595E-05	Ortholog(s) have Atg8-specific protease activity
C1_08750W_A	orf19.4733	YMC2	1.515779572	0.600059969	4.25334E-05	Putative mitochondrial carrier protein; Gcn4-regulated; F-12/ CO2 early biofilm induced; Spider biofilm induced
C5_04360C_A	orf19.3922	orf19.3922	1.51538097	0.599680536	4.25181E-09	Possible pyrimidine 5' nucleotidase; protein present in exponential and stationary growth phase yeast cultures; Hap43p-repressed gene
C2_03790C_A	orf19.842	ASR3	1.514846264	0.599171387	0.00035501	Adenylyl cyclase and stress responsive protein; induced in cyr1 or ras1 mutant; Spider biofilm induced
CR_04820W_A	orf19.6311	orf19.6311	1.514117026	0.598476715	0.000655898	Protein of unknown function; Hap43-induced; rat catheter and Spider biofilm induced
C5_01280C_A	orf19.1941	NUF2	1.513709889	0.598088731	9.05164E-08	Kinetochore component; amount of Nuf2p and Mtw1p protein detected at each centromere is consistent with a single kinetochore microtubule attachment site
C1_03260W_A	orf19.3019	orf19.3019	1.5132784	0.597677427	5.20014E-08	Putative DNA-dependent ATPase; transcription may be increased in an azole-resistant strain that overexpresses MDR1
C2_08870C_A	orf19.220	PIR1	1.511472063	0.595954313	2.73699E-05	1, 3-beta-glucan-linked cell wall protein; N-mannosylated, O-glycosylated by Pmt1; cell wall defect in het mutant; Hog1/fluconazole/hypoxia induced; iron/Efg1/Plc1/temp regulated; flow model biofilm induced; hyphal, Spider biofilm repressed
C1_06350W_A	orf19.6276	orf19.6276	1.510410823	0.594941008	0.000124074	Protein of unknown function; rat catheter biofilm repressed

C3_03720W_A	orf19.6947	GTT11	1.510173643	0.594714443	1.11639E-05	Glutathione S-transferase, localized to ER; induced in exponentially growing cells, under oxidative stress; induced by nitric oxide; Spider biofilm induced
C4_06980W_A	orf19.3111	PRA1	1.509612388	0.594178167	0.0276558	Cell surface protein that sequesters zinc from host tissue; enriched at hyphal tips; released extracellularly; binds to host complement regulators; mediates leukocyte adhesion and migration; immunogenic in mouse; produced at ambient pH
C4_02990C_A	orf19.2693	GST2	1.508573062	0.59318457	0.000144934	Glutathione S transferase; induced by benomyl and in populations of cells exposed to fluconazole over multiple generations; regulated by Nrg1, Tup1; induced by nitric oxide; stationary phase enriched; Spider biofilm induced
C6_03880W_A	orf19.5763	orf19.5763	1.508332687	0.592954673	0.0131792	Has domain(s) with predicted oxidoreductase activity and role in metabolic process
CR_03620C_A	orf19.4380.1	orf19.4380.1	1.506595434	0.591292062	1.77368E-08	Ortholog(s) have role in mitochondrial genome maintenance and integral component of mitochondrial inner membrane localization
C2_07660W_A	orf19.2216	PDS5	1.505475477	0.590219208	4.63204E-09	Putative protein with a predicted role in establishment and maintenance of sister chromatid condensation and cohesion; cell-cycle regulated periodic mRNA expression
C1_02060W_A	orf19.3663.1	orf19.3663.1	1.503478777	0.588304503	0.002831395	Predicted plasma membrane protein; gene has intron
C3_05210C_A	orf19.5999	DYN1	1.503472627	0.588298602	3.82529E-08	Dynein heavy chain; motor protein that moves to microtubule minus end; required for yeast cell separation, spindle positioning, nuclear migration, hyphal growth; regulated by Mig1, Hap43; flow model and rat catheter biofilm repressed
C4_00080C_A	orf19.376	orf19.376	1.502019334	0.586903384	0.000593693	Protein of unknown function; Hap43-repressed; Spider biofilm induced

C3_03640W_A	orf19.6956	DAL9	1.501954527	0.586841135	2.66423E-06	Putative allantoate permease; fungal-specific (no human or murine homolog)
CR_03850W_A	orf19.4356	HGT3	1.501808792	0.586701142	1.32752E-07	Putative glucose transporter of the major facilitator superfamily; the C. albicans glucose transporter family comprises 20 members; 12 probable membrane-spanning segments, extended C terminus; expressed in rich medium with 2% glucose
CR_06380C_A	orf19.727	orf19.727	1.501418567	0.586326229	0.00051932	Predicted ORF overlapping the Major Repeat Sequence on chromosome R; member of a family encoded by FGR6-related genes in the RB2 repeat sequence
C2_04210W_A	orf19.799	STE4	1.501348406	0.58625881	0.007497589	Beta subunit of heterotrimeric G protein of mating signal transduction pathway; required for mating; transcript is specific to cells homozygous at MTL; induced by alpha pheromone; ortholog of S. cerevisiae Ste4
C3_03120C_A	orf19.309	DAL5	1.500828044	0.585758691	0.009776877	Allantoate permease; nitrogen catabolite repressed, induced in absence of preferred N sources; nitrogen source regulation requires Gat1; possibly essential gene (by UAU1 method); Hap43-repressed
C1_14310W_A	orf19.7243	orf19.7243	1.500441845	0.585387403	6.88474E-09	Deoxycytidine monophosphate (dCMP) deaminase; role in dUMP and dTMP biosynthesis; Spider biofilm repressed
C5_04290C_A	orf19.3914	orf19.3914	0.66569968	-0.58705662	1.72603E-08	Has domain(s) with predicted translation initiation factor activity, role in translational initiation and cytoplasm localization
C2_06120C_A	orf19.4106	orf19.4106	0.664787755	-0.589034286	0.000799235	Ortholog(s) have ubiquitin conjugating enzyme binding activity
C7_00440C_A	orf19.7076	GBP2	0.664185477	-0.590341918	2.06301E-11	Putative single-strand telomeric DNA-binding protein; protein level decreases in stationary phase cultures; Spider biofilm repressed

C3_06310C_A	orf19.7401	ISW2	0.663942461	-0.590869876	3.30473E-07	Ortholog of S. cerevisiae Isw2; an ATPase involved in chromatin remodeling; required for chlamydospore formation; Hap43-induced gene; repressed by high-level peroxide stress
C4_00270W_A	orf19.5653	ATP2	0.663789173	-0.591202997	0.001502496	F1 beta subunit of F1F0 ATPase complex; antigenic in human, mice; induced by ciclopirox olamine; caspofungin repressed; macrophage/pseudohyphal-induced; detected during exponential and stationary growth phases; Spider biofilm repressed
C3_07810C_A	orf19.6176	SEC61	0.663553869	-0.591714502	7.95879E-10	ER protein-translocation complex subunit; essential; 10 predicted transmembrane regions; chimeric mutant partially functionally complements S. cerevisiae sec61 defects; Spider biofilm repressed
C1_02190W_A	orf19.3676	ABP140	0.663405528	-0.592037062	0.002478624	Ortholog of S. cerevisiae actin-binding protein Abp140; Hap43-induced; F-12/CO2 early biofilm induced
C1_01640W_A	orf19.3354	RPS42	0.66272137	-0.593525653	0.000958502	Predicted ribosomal protein S4, component of the small ribosomal subunit; has paralog RPS41
C3_03200C_A	orf19.319	orf19.319	0.662704223	-0.593562982	0.005270434	Ortholog ofS. cerevisiae: YDR286C, C. glabrata CBS138: CAGL0H01111g, C. dubliniensis CD36: Cd36_83160, C. parapsilosis CDC317: CPAR2_101820 and Candida tenuis NRRL Y-1498: CANTEDRAFT_113333
C1_03350C_A	orf19.3034	RLI1	0.662683097	-0.593608974	4.05502E-08	Member of RNase L inhibitor (RLI) subfamily of ABC family; predicted not to be a transporter; regulated by Sef1p, Sfu1p, and Hap43p
C7_01520W_A	orf19.6577	FLU1	0.662630548	-0.59372338	3.05604E-06	Multidrug efflux pump of the plasma membrane; MDR family member of the MFS (major facilitator superfamily) of transporters; involved in histatin 5 efflux; fungal-specific (no human/murine homolog)

C1_10880W_A	orf19.2330	orf19.2330	0.66237894	-0.59427129	8.65875E-06	Putative U3 snoRNA-associated protein; Hap43-induced; transposon mutation affects filamentous growth; repressed by prostaglandins
C2_05990C_A	orf19.4121	orf19.4121	0.662271838	-0.594504584	0.006596234	Predicted thioesterase/thiol ester dehydrase-isomerase; Spider biofilm induced
C2_00570W_A	orf19.2070	RSC58	0.661134567	-0.596984148	9.3854E-06	Component of the RSC chromatin remodeling complex
C1_12140W_A	orf19.5250	orf19.5250	0.661093163	-0.597074499	0.000423397	Ortholog of C. dubliniensis CD36: Cd36_11370, C. parapsilosis CDC317: CPAR2_207480, C. auris B8441: B9J08_001721 and Candida tenuis NRRL Y-1498: CANTEDRAFT_120384
C1_06900C_A	orf19.6220.3	MMD1	0.660813382	-0.597685193	9.13557E-08	Mitochondrial protein; possibly required for transamination of isoleucine; macrophage-downregulated protein abundance; rat catheter and Spider biofilm repressed
C4_05850C_A	orf19.1272	orf19.1272	0.660298539	-0.598809641	1.96436E-05	Protein of unknown function; may play a role in regulation of cell size; rat catheter biofilm repressed
C3_07930C_A	orf19.6188	orf19.6188	0.660244102	-0.598928585	0.028520111	Putative adhesin-like protein
C3_07440W_A	orf19.6745	TPI1	0.660218232	-0.598985115	9.22914E-05	Triose-phosphate isomerase; antigenic in mouse/human; mutation affects filamentation; macrophage-repressed; protein in exponential and stationary growth phase yeast; possibly essential; flow model biofilm induced; Spider biofilm repressed
C2_07820C_A	orf19.2199	PHO86	0.660071041	-0.59930679	5.8088E-05	Putative endoplasmic reticulum protein; possibly adherence-induced
C4_00670W_A	orf19.4175	TOK1	0.659544979	-0.600457044	8.39928E-05	Outwardly rectifying, noisily gated potassium channel; modulates sensitivity to human salivary histatin (Hst5); very similar to S. cerevisiae Tok1p; Bcr1-repressed in RPMI a/a biofilms

C2_02450C_A	orf19.1569	UTP22	0.659450281	-0.600664204	3.45908E-07	Putative U3 snoRNP protein; Ssr1-induced; repressed by prostaglandins; heterozygous null mutant is resistant to parnafungin
C6_00470C_A	orf19.4212	FET99	0.659436911	-0.600693453	5.02134E-06	Multicopper oxidase family protein; similar to S. cerevisiae Fet3; does not complement S. cerevisiae fet3 mutant growth under low-iron; iron-repressed; regulated by Tup1, Rim101; flow model biofilm induced; Spider biofilm repressed
C4_06670W_A	orf19.3142	orf19.3142	0.659334777	-0.600916916	0.001548774	Ortholog of C. dubliniensis CD36: Cd36_46140, C. parapsilosis CDC317: CPAR2_501210, C. auris B8441: B9J08_001807 and Candida tenuis NRRL Y-1498: CANTEDRAFT_97195
CR_09960C_A	orf19.7570	UGA3	0.659288792	-0.601017539	4.97467E-07	Zn(II)2Cys6 transcription factor; required for utilization of gamma-aminobutyrate (GABA) as a nitrogen source; mutants display decreased CFU in mouse kidneys; Spider biofilm induced
C1_02620C_A	orf19.2951	HOM6	0.657442895	-0.605062507	2.7015E-08	Putative homoserine dehydrogenase; Gcn4-regulated; induced by amino acid starvation (3-ATtreatment); macrophage-induced protein; protein level decreases in stationary phase cultures; flow model biofilm repressed
CR_10550W_A	orf19.7635	DRS1	0.657208573	-0.605576796	0.000518033	Putative nucleolar DEAD-box protein; Hap43-induced; mutation confers hypersensitivity to 5-fluorouracil (5-FU), tubercidin (7-deazaadenosine); Tbf1-induced; repressed in core stress response
CR_09950C_A	orf19.7569	SIK1	0.657186776	-0.605624644	6.27497E-08	Putative U3 snoRNP protein; Hap43p-induced gene; physically interacts with TAP-tagged Nop1p

CR_10680W_A	orf19.7655	RPO21	0.65688532	-0.60628657	3.42388E-09	RNA polymerase II; ortholog of S. cerevisiae Rpo21, transposon mutation affects filamentous growth; flow model biofilm repressed
C5_03020W_A	orf19.4328	CCC2	0.656414007	-0.60732207	1.05434E-08	Copper-transporting P-type ATPase of Golgi; required for wild-type iron assimilation (indirect effect via Fet3p); induced by iron starvation, ciclopirox olamine; caspofungin repressed; not required for virulence in mouse systemic infection
CR_10420W_A	orf19.7619	orf19.7619	0.656139343	-0.607925866	1.81288E-07	Protein with a mitochondrial distribution and morphology domain; possibly an essential gene, disruptants not obtained by UAU1 method; rat catheter and Spider biofilm induced
CR_07710W_A	orf19.1986	ARO2	0.656010669	-0.608208816	2.4423E-07	Putative chorismate synthase; fungal-specific (no human or murine homolog); protein level decreased in stationary phase yeast cultures; GlcNAc-induced protein
C4_00650W_A	orf19.4177	HIS5	0.655610012	-0.609090209	1.66086E-06	Putative histidinol-phosphate aminotransferase; Gcn4p-regulated; protein present in exponential and stationary growth phase yeast cultures
C6_01110W_A	orf19.118	FAD2	0.655539816	-0.609244686	1.65627E-06	Delta-12 fatty acid desaturase, involved in production of linoleic acid, which is a major component of membranes
C6_01120C_A	orf19.119	orf19.119	0.655395311	-0.609562744	0.04267388	Ortholog(s) have protein C-terminal S-isoprenylcysteine carboxyl O-methyltransferase activity
C1_01390C_A	orf19.3328	HOT1	0.655360855	-0.609638593	0.000992832	Putative transcription factor; required for inhibition of filamentous growth by farnesoic acid and for expression of PHO81; filament induced
C5_04860C_A	orf19.3972	orf19.3972	0.655228688	-0.60992957	0.018869326	Ortholog(s) have role in endoplasmic reticulum to Golgi vesicle-mediated transport, retrograde transport, endosome to Golgi

CR_00380W_A	orf19.7495	orf19.7495	0.654960898	-0.610519317	0.004037026	Protein with NADPH oxidoreductase containing flavin mononucleotide (FMN) domain; induced by nitric oxide
C3_07400W_A	orf19.6751	orf19.6751	0.654452345	-0.61163995	0.000175894	Ortholog(s) have tRNA (cytosine-2'-O-)-methyltransferase activity, tRNA (guanosine-2'-O-)-methyltransferase activity, tRNA 2'-O-methyltransferase activity
C2_02170W_A	orf19.1533	orf19.1533	0.653853833	-0.612959932	4.48433E-06	Possible vacuolar protein; Hap43-induced gene
C3_04900W_A	orf19.5959	NOP14	0.653853561	-0.612960533	5.11399E-05	Putative nucleolar protein; Hap43-induced; mutation confers resistance to 5-fluorocytosine (5-FC), 5-fluorouracil (5-FU), and tubercidin (7-deazaadenosine); heterozygous mutant is resistant to parnafungin; Spider biofilm induced
C1_03430W_A	orf19.3043	orf19.3043	0.653020218	-0.614800436	0.001039979	Ortholog(s) have triglyceride lipase activity, role in triglyceride catabolic process and mitochondrion localization
C3_06980W_A	orf19.6803	HUT1	0.652299328	-0.616393952	0.000162972	Ortholog(s) have UDP-galactose transmembrane transporter activity and role in UDP-galactose transmembrane transport, UDP-glucose transmembrane transport
C1_03910C_A	orf19.4465	orf19.4465	0.652240597	-0.616523855	7.62132E-07	Protein of unknown function; Spider biofilm induced
C5_01900C_A	orf19.3181.1	orf19.3181.1	0.651886256	-0.617307837	0.002630869	Protein of unknown function; involved in secretion of proteins that lack classical secretory signal sequences; Spider biofilm induced
C3_06740W_A	orf19.6829	orf19.6829	0.651609733	-0.617919941	5.03103E-05	Protein with a predicted mitochondrial ATPase expression domain; possibly an essential gene, disruptants not obtained by UAU1 method
C4_00690C_A	orf19.4173	orf19.4173	0.650582681	-0.620195678	0.000148026	Ortholog(s) have role in peptidyl-diphthamide biosynthetic process from peptidyl-histidine

CR_00340C_A	orf19.7499.1	orf19.7499.1	0.650241777	-0.620951844	0.000136212	Ortholog of C. parapsilosis CDC317: CPAR2_800380, Candida tenuis NRRL Y-1498: CANTEDRAFT_114079, Pichia stipitis Pignal: psti_CGOB_00127 and Candida tropicalis MYA-3404: CTRG_05490
C4_06110C_A	orf19.4718	TRP5	0.649851091	-0.621818923	2.64092E-10	Predicted tryptophan synthase; identified in detergent-resistant membrane fraction (possible lipid raft component); predicted N-terminal acetylation; Gcn4p-regulated; S. cerevisiae ortholog is Gcn4p regulated; upregulated in biofilm;
C2_06810C_A	orf19.2232	RPL11	0.64975333	-0.622035973	1.23317E-12	Ribosomal protein; repressed by phagocytosis; colony morphology-related gene regulation by Ssn6; Hap43-induced; Spider biofilm repressed
C6_02220W_A	orf19.3462	SAR1	0.649087619	-0.623514857	0.006300837	Functional homolog of S. cerevisiae Sar1; which is required for ER-to-Golgi protein transport; binds GTP; similar to small GTPase superfamily proteins; gene has intron; Hap43-induced; rat catheter biofilm repressed
C4_02400C_A	orf19.2763	orf19.2763	0.648994336	-0.623722207	3.569E-06	Protein not essential for viability; orf19.10279 possibly transcriptionally regulated upon hyphal formation
C3_06420C_A	orf19.7427	orf19.7427	0.648736881	-0.624294636	1.04964E-07	Has domain(s) with predicted integral component of membrane localization
CR_04870C_A	orf19.6306	orf19.6306	0.648431524	-0.624973864	2.35861E-07	Trimethylaminobutyraldehyde dehydrogenase, the third enzyme of the carnitine biosynthesis pathway
CR_00900W_A	orf19.3265	TRM1	0.648394307	-0.625056671	6.5595E-08	Putative N2, N2-dimethylguanine tRNA methyltransferase; induced upon adherence to polystyrene
C6_00490W_A	orf19.4210	orf19.4210	0.648025653	-0.625877169	0.003357149	Ortholog(s) have phospholipase A2 activity, role in cardiolipin acyl-chain remodeling, cardiolipin metabolic process and mitochondrial inner membrane, mitochondrion localization

C3_02320W_A	orf19.1613	ILV2	0.647951504	-0.626042256	4.38442E-13	Putative acetolactate synthase; regulated by Gcn4p; induced by amino acid starvation (3-AT treatment); stationary phase enriched protein
C6_01910W_A	orf19.3391	ADK1	0.647797545	-0.626385094	4.58563E-09	Putative adenylate kinase; repressed in hyphae; macrophage-induced protein; adenylate kinase release used as marker for cell lysis; possibly essential (UAU1 method); flow model biofilm induced; rat catheter and Spider biofilm repressed
C5_00480C_A	orf19.941	SEC14	0.647462854	-0.62713067	6.46497E-06	Essential protein; functional homolog of S. cerevisiae Sec14p, a Golgi phosphatidylinositol/phosphatidylcholine transfer protein that regulates choline-phosphate cytidyltransferase and thereby affects secretion; biofilm-regulated
C2_02370C_A	orf19.1559	HOM2	0.646901775	-0.628381423	4.76188E-09	Aspartate-semialdehyde dehydrogenase; forms a homodimer; conserved in bacteria, archaea, and fungi but not in mammals; ketoconazole-repressed; protein present in exponential and stationary growth phase yeast cultures; GlcNAc-induced protein
C4_05110C_A	orf19.740	HAP41	0.646626481	-0.628995503	0.000702797	Putative Hap4-like transcription factor; Hap43-repressed; not required for response to low iron; induced by Mnl1 under weak acid stress; Spider biofilm induced
C1_02210W_A	orf19.3678	orf19.3678	0.646372257	-0.629562817	1.41993E-05	Ortholog of C. dubliniensis CD36: Cd36_02060, C. parapsilosis CDC317: CPAR2_106200, Candida tenuis NRRL Y-1498: CANTEDRAFT_96322 andDebaryomyces hansenii CBS767: DEHA2C02310g
C4_02640C_A	orf19.2735	SEN2	0.6457834	-0.630877737	2.11381E-05	Putative tRNA splicing endonuclease subunit; mutation confers hypersensitivity to toxic ergosterol analog and to

						amphotericin B; 5'-UTR intron; Hap43-induced; Spider biofilm induced
C4_02010C_A	orf19.4592	HSX11	0.645704189	-0.631054708	3.45992E-06	UDP-glucose:ceramide glucosyltransferase (glucosylceramide synthase [GCS], EC 2.4.1.80); involved in glucosylceramide biosynthesis, which is important for virulence
C4_00140C_A	orf19.5639	HIS4	0.644458863	-0.633839823	2.53696E-12	Multifunctional enzyme that catalyzes three steps of histidine biosynthesis, with phosphoribosyl-AMP cyclohydrolase, phosphoribosyl-ATP diphosphatase, and histidinol dehydrogenase activities; required for wild-type adhesion to human calls
C2_09780C_A	orf19.1372	orf19.1372	0.644439078	-0.633884115	1.71503E-06	Protein of unknown function; transcript regulated by Mig1 and Tup1
C1_06090C_A	orf19.2436	SKY1	0.644354796	-0.634072809	1.14637E-05	SR-like protein kinase involved in osmotic stress and polyamine resistance; expression analysis suggests roles in mRNA processing and mitochondrial function; Spider biofilm induced; null mutant shows resistance to hygromycin B
C2_09040W_A	orf19.200	orf19.200	0.644313974	-0.63416421	1.46804E-06	Putative nuclear RNA-binding protein; Spider biofilm repressed
C6_02290C_A	orf19.3470	orf19.3470	0.64397896	-0.634914542	0.000573389	Putative flavodoxin; similar to S. cerevisiae Tyw1, an iron-sulfur protein required for synthesis of wybutosine modified tRNA; predicted Kex2p substrate; Spider biofilm induced
CR_06810W_A	orf19.1853	HHT2	0.643526416	-0.635928725	0.001874425	Putative histone H3; farnesol regulated; Hap43-induced; rat catheter and Spider biofilm repressed

CR_02650C_A	orf19.2825	DRE2	0.642745433	-0.63768064	1.73894E-06	Putative cytosolic Fe-S protein assembly protein; a-specific transcript; regulated by Sef1, Sfu1, and Hap43; rat catheter and Spider biofilm induced
CR_05680C_A	orf19.6644	orf19.6644	0.642386096	-0.638487429	0.000609253	Protein of unknown function; 2 predicted transmembrane domains; transcript detected on high-resolution tiling arrays; flow model biofilm induced; Spider biofilm repressed
C4_06570C_A	orf19.2877	PDC11	0.641698177	-0.64003321	6.62961E-05	Pyruvate decarboxylase; antigenic; on hyphal not yeast cell surface; Hap43, Gcn4, Efg1, Efh1, Hsf1 regulated; fluconazole, farnesol induced; amino acid starvation repressed; flow model biofilm induced; Spider biofilm repressed
C2_02680W_A	orf19.5839	PDR17	0.641687685	-0.640056799	5.94792E-06	Fungal-specific protein (no human or murine homolog); role in sensitivity to fluconazole, specifically
C3_00650W_A	orf19.5392	NGT1	0.641631257	-0.64018367	0.018701888	N-acetylglucosamine (GlcNAc)-specific transporter; role in GlcNAc (but not serum) induced hyphal growth; localizes to plasma membrane; induced by GlcNAc, macrophage engulfment; 12 transmembrane, major facilitator superfamily
C2_05130W_A	orf19.3544	orf19.3544	0.64069584	-0.642288472	2.50323E-07	Putative protein of unknown function; Hap43p-repressed gene
C4_01690C_A	orf19.4624	HRT2	0.640601353	-0.64250125	1.83217E-06	Protein described as having a role in Ty3 transposition; repressed in hyphae; stationary phase enriched protein; rat catheter and Spider biofilm repressed
C1_08580C_A	orf19.405	VCX1	0.639798063	-0.64431147	4.09119E-06	Putative H+/Ca2+ antiporter; Spider biofilm repressed
C2_04480W_A	orf19.4504	orf19.4504	0.639037396	-0.646027737	2.27107E-05	Has domain(s) with predicted oxidoreductase activity, zinc ion binding activity
CR_08580C_A	orf19.6431	orf19.6431	0.63869723	-0.646795901	0.001334787	Protein of unknown function; Spider biofilm induced

C5_01710C_A	orf19.3204	orf19.3204	0.638695007	-0.646800922	0.000828928	Ortholog of C. dubliniensis CD36: Cd36_51610, C. parapsilosis CDC317: CPAR2_303630, C. auris B8441: B9J08_001459 and Candida tenuis NRRL Y-1498: CANTEDRAFT_135125
C3_02040C_A	orf19.1642	orf19.1642	0.63857442	-0.647073333	0.000452064	Ortholog of S. cerevisiae Loc1, a nuclear protein involved in asymmetric localization of ASH1 mRNA in S. cerevisiae; Hap43-induced gene; Spider biofilm induced
C2_08660C_A	orf19.3610	orf19.3610	0.63839386	-0.64748132	0.000662093	Protein of unknown function; upregulation correlates with clinical development of fluconazole resistance; regulated by Sef1, Sfu1, and Hap43
C5_03970W_A	orf19.3214	orf19.3214	0.638054563	-0.648248294	0.021739887	Alpha/beta-HydrolasE superfamily protein; membrane-localized
C4_03930C_A	orf19.789.1	orf19.789.1	0.638046463	-0.648266609	3.48376E-05	Ortholog(s) have 7S RNA binding activity, role in SRP-dependent cotranslational protein targeting to membrane, translocation and signal recognition particle, endoplasmic reticulum targeting localization
CR_06860C_A	orf19.1847	ARO10	0.63791742	-0.64855842	1.89557E-10	Aromatic decarboxylase; Ehrlich fusel oil pathway of aromatic alcohol biosynthesis; alkaline repressed; protein abundance affected by URA3 expression in CAI-4 strain; Spider biofilm induced
C6_02090C_A	orf19.3501	orf19.3501	0.637659178	-0.649142569	2.29514E-05	S. cerevisiae ortholog Pxl1 localizes to sites of polarized growth and is required for selection and/or maintenance of polarized growth sites; Hog1p-repressed
CR_02060W_A	orf19.2607	orf19.2607	0.63672378	-0.651260448	4.27792E-09	Protein of unknown function; Spider biofilm induced
C7_03050W_A	orf19.5156	orf19.5156	0.636660467	-0.65140391	0.000785683	Protein similar to S. cerevisiae Phs1p, which is required for growth; has six putative membrane-spanning regions
C4_03380C_A	orf19.3365	DAO2	0.635660065	-0.653672641	9.57413E-06	Putative D-amino acid oxidase; rat catheter biofilm induced

C1_02530C_A	orf19.2942	DIP5	0.63561643	-0.653771678	2.03038E-11	Dicarboxylic amino acid permease; mutation confers hypersensitivity to toxic ergosterol analog; induced upon phagocytosis by macrophage; Gcn4-regulated; upregulated by Rim101 at pH 8; rat catheter and Spider biofilm induced
CR_09110C_A	orf19.7307	orf19.7307	0.63540613	-0.654249085	0.000758781	Putative oxidoreductase; similar to S. cerevisiae Pga3p; possible Kex2p substrate
C5_03850W_A	orf19.1109	orf19.1109	0.635000184	-0.655171084	0.00016015	Ortholog of C. dubliniensis CD36: Cd36_53580, C. parapsilosis CDC317: CPAR2_303030, Debaryomyces hansenii CBS767: DEHA2G07766g andPichia stipitis Pignal: psti_CGOB_00176
C6_00530C_A	orf19.4205.1	orf19.4205.1	0.634946729	-0.655292538	0.005883083	Ortholog(s) have splicing factor binding activity, role in mRNA splicing, via spliceosome and U1 snRNP, U2 snRNP, U2-type prespliceosome, U4/U6 x U5 tri-snRNP complex, U5 snRNP, post-mRNA release spliceosomal complex localization
C2_06680W_A	orf19.1224	FRP3	0.634757162	-0.655723326	2.31167E-07	Putative ammonium transporter; upregulated in the presence of human neutrophils; fluconazole-downregulated; repressed by nitric oxide; Spider biofilm induced; rat catheter biofilm repressed
C4_05050C_A	orf19.3774	PPG1	0.634749713	-0.655740258	3.73293E-06	Protein phosphatase of the Type 2A-related family (serine/threonine-specific) involved in control of filamentous growth and virulence
CR_10660W_A	orf19.7652	CKA1	0.634246994	-0.656883319	6.17401E-08	Putative alpha subunit (catalytic subunit) of protein kinase CK2; Cka1p and Cka2p have a common target with respect to fluconazole resistance; synthetically lethal with CKA2; flucytosine induced
C1_12790C_A	orf19.6343	FEN1	0.634126898	-0.657156522	1.68949E-06	Putative fatty acid elongase; predicted role in sphingolipid biosynthesis; possibly an essential gene, disruptants not

						obtained by UAU1 method; Spider and flow model biofilm induced
C1_00800C_A	orf19.6026	ERG2	0.633928696	-0.657607519	1.04581E-05	C-8 sterol isomerase; enzyme of ergosterol biosynthesis; converts fecosterol to episterol; mutant is hypersensitive to multiple drugs; ketoconazole-induced; flow model and Spider biofilm repressed
C4_04360W_A	orf19.1411	orf19.1411	0.632961643	-0.659810018	6.56058E-05	Putative cytochrome P450; Hap43-repressed gene
CR_10690W_A	orf19.7657	POP3	0.632935147	-0.659870411	0.010119699	Putative RNase MRP and nuclear RNase P component; decreased repressed by prostaglandins; Spider biofilm induced
CR_10780C_A	orf19.7667	IAH1	0.632831366	-0.660106986	5.60228E-07	Protein similar to S. cerevisiae Iah1p, which is involved in acetate metabolism; mutation confers hypersensitivity to tunicamycin; transposon mutation affects filamentous growth
C1_11500C_A	orf19.1170	ARO7	0.632753206	-0.660285184	3.18256E-10	Putative chorismate mutase; fungal-specific (no human or murine homolog); alkaline upregulated
C3_04510W_A	orf19.5905	orf19.5905	0.632031685	-0.66193121	0.000522295	Protein of unknown function; Hap43-induced; F-12/CO2 early biofilm induced
C1_05080W_A	orf19.4063	GPT1	0.631415166	-0.663339183	0.000152001	GABA/polyamine transporter; 9 to 11 membrane spanning segments; complements GABA uptake defect of an S. cerevisiae uga4 put4 gap1 triple mutant; complements growth of an S. cerevisiae spe1 mutant under polyamine limitation
C1_10200C_A	orf19.4889	orf19.4889	0.630071265	-0.666413079	4.24488E-09	Predicted MFS family membrane transporter, member of the drug:proton antiporter (12 spanner) (DHA1) family; Spider biofilm induced
C3_07650C_A	orf19.6723	orf19.6723	0.62994393	-0.666704673	0.000151169	Protein of unknown function; Spider biofilm induced

C7_01660C_A	orf19.6558	orf19.6558	0.628993714	-0.668882496	5.24111E-09	Ortholog(s) have GTPase activator activity and cytosol localization
C4_05680W_A	orf19.1253	PHO4	0.628796307	-0.66933535	6.76244E-09	bHLH transcription factor of the myc-family; required for phosphate acquisition and for resistance to stresses; induced by Mnl1 under weak acid stress
CR_09620C_A	orf19.6600	orf19.6600	0.62855323	-0.669893167	0.000943258	Ortholog(s) have phosphatidic acid transfer activity and role in cardiolipin metabolic process, phospholipid translocation, phospholipid transport, positive regulation of phosphatidylcholine biosynthetic process
C2_06390C_A	orf19.18	IMH3	0.628527549	-0.669952115	1.62232E-06	Inosine monophosphate (IMP) dehydrogenase; enzyme of GMP biosynthesis; target of mycophenolic acid and mizoribine monophosphate; antigenic during infection; repressed in core stress response; snoRNA snR54 encoded within IMH3 intron
C4_00660W_A	orf19.4176	orf19.4176	0.628240841	-0.670610361	2.64338E-05	Ortholog(s) have structural constituent of ribosome activity and mitochondrial small ribosomal subunit localization
C1_01520C_A	orf19.3340	SOD2	0.628021182	-0.671114876	9.16454E-06	Mitochondrial Mn-containing superoxide dismutase; protection against oxidative stress; homotetramer active; N-terminal 34 amino acids removed on mitochondrial import; H2O2-induced via Cap1p; Hap43p-, alkaline-downregulated, farnesol-induced
C2_09750W_A	orf19.1375	LEU42	0.627749401	-0.671739347	1.63675E-10	Putative alpha-isopropylmalate synthase; fungal-specific; induced by human blood or polymorphonuclear cells; regulated by Gcn2 and Gcn4; stationary phase enriched protein; Spider biofilm induced
CR_10500C_A	orf19.7627	orf19.7627	0.62767731	-0.671905038	0.000127355	Ortholog ofS. cerevisiae: YNL320W, C. glabrata CBS138: CAGL0M04125g, C. dubliniensis CD36: Cd36_35340, C.

						parapsilosis CDC317: CPAR2_200350 and C. auris B8441: B9J08_001244
C2_09430W_A	orf19.1601	RPL3	0.627635401	-0.672001367	3.66682E-07	Ribosomal protein, large subunit; induced by ciclopirox olamine treatment; genes encoding cytoplasmic ribosomal subunits are downregulated upon phagocytosis by murine macrophages; Hap43-induced gene; Spider biofilm repressed
C3_01480C_A	orf19.1701	RKI1	0.627556764	-0.672182135	1.98681E-05	Ortholog(s) have ribose-5-phosphate isomerase activity and role in pentose-phosphate shunt, pyridoxine biosynthetic process
C5_01070C_A	orf19.1964	orf19.1964	0.627228112	-0.672937873	1.69513E-08	Protein of unknown function; repressed by fluphenazine treatment; induced by benomyl treatment and in an RHE model; regulated by Nrg1, Tup1
C3_07450C_A	orf19.6744	orf19.6744	0.626938527	-0.673604105	1.39647E-05	Has domain(s) with predicted endodeoxyribonuclease activity, producing 5'-phosphomonoesters activity
C3_02460C_A	orf19.234	PHA2	0.62622564	-0.675245515	2.80059E-05	Putative prephenate dehydratase; Hap43p-repressed gene; expression downregulated in an ssr1 null mutant
CR_09490W_A	orf19.7344	orf19.7344	0.625760581	-0.676317315	0.000248541	Ortholog(s) have DNA binding, chromatin binding, histone deacetylase activity and role in chromosome segregation, gene silencing by RNA, histone deacetylation, negative regulation of transcription by RNA polymerase II
C2_01120W_A	orf19.2013	KAR2	0.625134455	-0.677761573	9.65276E-08	Similar to Hsp70 family chaperones; role in translocation of proteins into the ER; induced in high iron; protein present in exponential and stationary growth phase yeast cultures; flow model and Spider biofilm repressed
C1_10650W_A	orf19.1836	APN2	0.624868973	-0.678374389	0.000110914	Putative class II abasic (AP) endonuclease; flucytosine induced

C1_04180W_A	orf19.1052	orf19.1052	0.62481088	-0.678508518	0.001322003	Predicted histone H2B; Hap43-induced gene; Spider biofilm repressed
C3_02860W_A	orf19.277	THI6	0.624470751	-0.679294094	1.07765E-06	Putative thiamin-phosphate pyrophosphorylase, hydroxyethylthiazole kinase; fungal-specific; Spider biofilm induced
C1_01370C_A	orf19.3325.3	RPS21B	0.623651832	-0.681187258	3.17359E-08	Ribosomal protein S21; regulated by Nrg1, Tup1; colony morphology-related gene regulation by Ssn6; positively regulated by Tbf1, Hap43; Spider biofilm repressed
CR_09440C_A	orf19.7341	orf19.7341	0.623194199	-0.682246291	5.45941E-05	Protein of unknown function; flow model biofilm induced; ketoconazole-repressed
C3_05280C_A	orf19.6007	orf19.6007	0.622978391	-0.682745973	7.354E-05	Predicted fatty acid acyl transferase-related protein domain; repressed by prostaglandins
CR_06700C_A	orf19.704	SOL3	0.621666236	-0.685787871	1.93093E-05	Putative 6-phosphogluconolactonase; present in exponential and stationary growth phase yeast cultures; macrophage-downregulated protein; Spider biofilm repressed
CR_02540W_A	orf19.169	CHO2	0.621464665	-0.686255728	3.09213E-09	Phosphatidyl-ethanolamine N-methyltransferase; fungal-specific (no human or murine homolog); amphotericin B repressed; Hap43p-induced gene
C1_02820W_A	orf19.2970	LYS2	0.621448514	-0.686293224	1.69337E-09	Heterodimeric alpha-aminoadipate reductase large subunit; lysine biosynthesis; predicted binding sites for AMP and alpha-aminoadipate; inhibited by lys or thialysine; regulated by Gcn2 and Gcn4; Spider biofilm induced, flow model repressed
CR_10520C_A	orf19.7631	SLD5	0.620807128	-0.687782973	0.025154386	Putative GINS complex subunit; cell-cycle regulated periodic mRNA expression
CR_09550C_A	orf19.6607	NUO1	0.620196477	-0.689202764	2.53894E-06	NADH-ubiquinone oxidoreductase subunit with roles in mitochondrial respiratory chain complex I assembly

C1_04510W_A	orf19.6845	orf19.6845	0.620065442	-0.689507607	0.000633008	Putative transcription factor with bZIP DNA-binding motif; rat catheter biofilm induced
C5_03500W_A	orf19.6659	GAP6	0.619215868	-0.691485652	9.41398E-08	Broad-specificity amino acid permease; Plc1, Gcn4 regulated; rat catheter biofilm induced
C3_06870W_A	orf19.6814	TDH3	0.618884771	-0.692257273	5.91089E-07	NAD-linked glyceraldehyde-3-phosphate dehydrogenase; binds fibronectin, laminin; at cell surface; antigenic in infection; farnesol-repressed; stationary phase-enriched; GlcNAc-induced; flow model biofilm induced; Spider biofilm repressed
CR_08840C_A	orf19.7279	NIT2	0.618821027	-0.692405876	4.30242E-05	Putative carbon-nitrogen hydrolase; rat catheter biofilm repressed
CR_09210W_A	orf19.7319	SUC1	0.618726307	-0.692626719	0.001932524	Zinc-finger transcription factor; regulates alpha-glucosidase exression; complements S. cerevisiae suc2 for sucrose utilization and mal13 maltase defect; required for yeast cell adherence to silicone substrate; rat catheter biofilm induced
C2_05770W_A	orf19.6888	orf19.6888	0.618639149	-0.692829962	0.003198696	Zn(II)2Cys6 domain transcription factor; regulated by Mig1 and Tup1; rat catheter and Spider biofilm induced
C6_00860W_A	orf19.87	GPX1	0.618070613	-0.694156423	3.01658E-06	Putative thiol peroxidase; rat catheter and Spider biofilm induced
CR_09980W_A	orf19.7572	SPT7	0.617996575	-0.694329253	9.65891E-11	Putative SAGA transcriptional regulatory complex subunit; mutation confers hypersensitivity to toxic ergosterol analog, and to amphotericin B
C4_01500W_A	orf19.4634	orf19.4634	0.617529962	-0.695418959	1.04918E-05	Protein required for thiolation of uridine at wobble position of Gln, Lys, and Glu tRNAs; has a role in urmylation; S. cerevisiae ortholog has a role in invasive and pseudohyphal growth

C6_00870C_A	orf19.88	ILV5	0.61748594	-0.695521808	1.56143E-09	Ketol-acid reductoisomerase; antigenic; regulated by Gcn4; GlcNAc, amino acid starvation (3-AT)-induced; macrophage-repressed protein; protein present in exponential and stationary phase; flow model and Spider biofilm repressed
C2_08490W_A	orf19.3629	DSE1	0.616965893	-0.696737358	6.75247E-06	Essential cell wall protein involved in cell wall integrity and rigidity; periodic mRNA expression peaks at M/G1 phase; Ace2p-induced; required for virulence in a mouse model of infection
C6_01670W_A	orf19.3419	MAE1	0.616501924	-0.697822697	1.32873E-07	Malic enzyme, mitochondrial; transcription regulated by Mig1, Tup1; colony morphology-related gene regulation by Ssn6; Hap43-repressed; Spider biofilm repressed
C1_11510C_A	orf19.1169	orf19.1169	0.616331537	-0.698221481	0.000297452	Putative DnaJ-like molecular chaperone; Spider biofilm induced
C1_09350W_A	orf19.4796	orf19.4796	0.616214196	-0.698496177	3.97122E-08	Putative eIF-4E-binding repressor of CAP-dependent translation; stationary phase enriched protein
CR_01030W_A	orf19.3252	DAL81	0.616095251	-0.69877468	2.17398E-07	Zn(II)2Cys6 transcription factor; ortholog of S. cerevisiae Dal81, involved in the regulation of nitrogen-degradation genes; required for yeast cell adherence to silicone substrate; Spider biofilm induced
CR_04590C_A	orf19.550	PDX3	0.615424744	-0.700345644	4.44867E-07	Pyridoxamine-phosphate oxidase; transcript regulated by yeast-hypha switch and by Nrg1, Mig1, Tup1; Hap43, caspofungin repressed; present in exponential and stationary phase yeast cultures
C2_01210C_A	orf19.2003	HNM1	0.614687795	-0.702074254	7.51302E-08	Putative choline/ethanolamine transporter; mutation confers hypersensitivity to toxic ergosterol analog; colony morphology-related gene regulation by Ssn6; clade-associated gene expression

C6_02350C_A	orf19.3477	orf19.3477	0.613835292	-0.7040765	0.000376772	Putative pseudouridine synthase; predicted role in snRNA pseudouridine synthesis, tRNA pseudouridine synthesis; Spider biofilm induced
CR_09600C_A	orf19.6601.1	YKE2	0.613634005	-0.704549662	5.84035E-05	Possible heterohexameric Gim/prefoldin protein complex subunit; role in folding alpha-tubulin, beta-tubulin, and actin; transcript induced by yeast-to-hypha switch; regulated by Nrg1, Tup1; Spider and flow model biofilm induced
C4_01750C_A	orf19.4618	FBA1	0.613405313	-0.705087431	6.7674E-05	Fructose-bisphosphate aldolase; glycolytic enzyme; antigenic in murine/human infection; regulated by yeast-hypha switch; induced by Efg1, Gcn4, Hog1, fluconazole; phagocytosis-repressed; flow model biofilm induced; Spider biofilm repressed
C3_07470W_A	orf19.6741	EVP1	0.611798979	-0.708870396	2.23233E-06	Putative plasma membrane protein; predicted role in cell wall integrity; regulated by Nrg1, Tup1; induced during chlamydospore formation in both C. albicans and C. dubliniensis
C1_01580W_A	orf19.3348	orf19.3348	0.611161407	-0.71037465	4.53396E-05	Ortholog(s) have structural constituent of ribosome activity and fungal-type vacuole, mitochondrial large ribosomal subunit localization
CR_09160C_A	orf19.7312	ERG13	0.609949825	-0.713237525	1.80679E-12	3-hydroxy-3-methylglutaryl coenzyme A synthase; ergosterol biosynthesis; sumoylation target; Tn mutation affects filamentation; amphotericin B, caspofungin repressed; exponential, stationary growth phase expressed; Spider biofilm repressed
C3_04270C_A	orf19.5870	CTP1	0.607979519	-0.717905371	1.63932E-07	Putative citrate transport protein; flucytosine induced; amphotericin B repressed, caspofungin repressed; Hap43p-induced gene

C4_06560W_A	orf19.2878	PGA15	0.607908174	-0.718074677	0.001249958	Putative GPI-anchored protein
C6_03390W_A	orf19.5628	orf19.5628	0.607414545	-0.71924664	1.3119E-09	Mitochondrial dicarboxylate transporter; possibly an essential gene, disruptants not obtained by UAU1 method
CR_00290W_A	orf19.7504	orf19.7504	0.607049602	-0.720113692	0.000298919	Ortholog of S. cerevisiae Rts3; a component of the protein phosphatase type 2A complex; Plc1-regulated; induced in core caspofungin response; Spider biofilm induced
C1_01170C_A	orf19.3305	ZCF17	0.606923752	-0.720412812	4.76275E-08	Putative Zn(II)2Cys6 transcription factor
C1_11580W_A	orf19.1162	orf19.1162	0.606904532	-0.720458502	5.54255E-08	Protein of unknown function; transcript upregulated by benomyl treatment
C2_09350W_A	orf19.4096	TAZ1	0.60682408	-0.720649759	0.001010139	Putative lyso-phosphatidylcholine acyltransferase, required for normal phospholipid content of mitochondrial membranes; rat catheter biofilm induced
C2_06360C_A	orf19.5500	MAK16	0.606270656	-0.721966099	0.000275497	Putative constituent of 66S pre-ribosomal particles; Hap43-induced; repressed by prostaglandins; Spider biofilm induced
C7_00380W_A	orf19.7082	orf19.7082	0.606219477	-0.722087889	2.45356E-09	S-adenosylmethionine transporter of the mitochondrial inner membrane; mitochondrial carrier family; predicted role in biotin biosynthesis and respiratory growth; Spider biofilm repressed
CR_00630W_A	orf19.7468	VHR1	0.605788507	-0.723113887	4.83595E-07	Transcriptional activator of genes involved in biotin metabolism; required for survival and proliferation in macrophages; expression upregulated during growth in the mouse cecum; Spider biofilm induced
C2_03940C_A	orf19.829	SCH9	0.604454873	-0.726293459	1.96351E-08	Protein kinase; involved in growth control, ribosomal protein synthesis, cell size, resistance to rapamycin, , chlamydospore formation, filamentous growth, and virulence; prevents hyphal growth in hypoxia at high CO2

C3_00640W_A	orf19.5393	orf19.5393	0.603067391	-0.729608867	7.65869E-08	Putative cysteine sulfinate decarboxylase; transcript positively regulated by Tbf1; Spider biofilm induced
C4_04320W_A	orf19.1415	FRE10	0.602847428	-0.730135173	2.39998E-08	Major cell-surface ferric reductase under low-iron conditions; 7 transmembrane regions and a secretion signal predicted; Tup1, Rim101, Ssn6, Hog1, caspofungin repressed; ciclopirox olamine induced; rat catheter biofilm induced
C4_03720C_A	orf19.1306	orf19.1306	0.602647536	-0.730613621	1.03723E-06	Has domain(s) with predicted 2-oxoglutarate-dependent dioxygenase activity
C3_04340W_A	orf19.5877	ATF1	0.602316774	-0.731405657	3.19838E-12	Putative alcohol acetyltransferase; caspofungin repressed; expression depends on Tac1p
CR_10290C_A	orf19.7604	orf19.7604	0.602261554	-0.731537929	3.59052E-10	Ortholog(s) have role in early endosome to Golgi transport, establishment or maintenance of cell polarity, hyphal growth, intra-Golgi vesicle-mediated transport, regulation of GTPase activity
C1_04300C_A	orf19.1065	SSA2	0.60225898	-0.731544095	7.354E-11	HSP70 family chaperone; cell wall fractions; antigenic; beta-defensin peptides impport; ATPase domain binds histatin 5; at hyphal surface, not yeast; farnesol-repressed in biofilm; flow model, Spider biofilm repressed; caspofungin repressed
CR_05380C_A	orf19.5286	YCP4	0.601774828	-0.732704334	9.46798E-09	Flavodoxin-like protein involved in oxidative stress protection and virulence; flow model, rat catheter and Spider biofilm repressed
CR_10350C_A	orf19.7611	TRX1	0.600080481	-0.736772091	3.82099E-10	Thioredoxin; involved in response to reactive oxygen species; biofilm, benomyl, flucytosine, peroxide, Hap43 induced; amphotericin B, caspofungin repressed; induced by human neutrophils; macrophage-repressed gene

C1_04620W_A	orf19.6855	orf19.6855	0.599644777	-0.737819979	1.16899E-08	Ortholog of C. dubliniensis CD36: Cd36_04370, C. parapsilosis CDC317: CPAR2_105410, C. auris B8441: B9J08_000021 and Candida tenuis NRRL Y-1498: CANTEDRAFT_114891
CR_10390W_A	orf19.7615	orf19.7615	0.599377232	-0.738463813	2.06543E-05	Protein involved in endoplasmic reticulum (ER) to Golgi vesicle-mediated transport; putative subunit of the transport protein particle (TRAPP) complex of the cis-Golgi; Spider biofilm induced
C1_10440W_A	orf19.985	orf19.985	0.599086474	-0.739163834	2.04062E-15	Ortholog ofS. cerevisiae: YEL043W, C. glabrata CBS138: CAGL0L01221g, C. dubliniensis CD36: Cd36_09830, C. parapsilosis CDC317: CPAR2_804210 and C. auris B8441: B9J08_004773
C1_13070C_A	orf19.4933	FAD3	0.597850023	-0.74214448	6.91278E-09	Omega-3 fatty acid desaturase; production of alpha-linolenic acid, a major component of membranes; caspofungin induced; Plc1-regulated; colony morphology-related gene regulation by Ssn6; Spider biofilm induced, flow model biofilm repressed
C1_04290C_A	orf19.1064	ACS2	0.597242614	-0.743610986	1.00729E-09	Acetyl-CoA synthetase; antigenic during human and murine infection; induced by Efg1; macrophage-induced protein; soluble protein in hyphae; gene contains intron; flow model and Spider biofilm repressed
C5_03790W_A	orf19.1115	GUK1	0.597235887	-0.743627238	8.21983E-11	Putative guanylate kinase; identified in extracts from biofilm and planktonic cells; protein level decrease in stationary phase cultures; Hap43p-induced gene
C3_00930W_A	orf19.2496	ATO2	0.596832696	-0.744601523	2.67761E-08	Putative fungal-specific transmembrane protein; fluconazole repressed, Hap43-repressed; flow model biofilm induced; Spider biofilm induced

C5_01470C_A	orf19.4142	orf19.4142	0.596455624	-0.74551329	3.33761E-05	Putative transporter; decreased transcription is observed upon fluphenazine treatment
CR_10750C_A	orf19.7664	orf19.7664	0.595857861	-0.746959871	0.004032745	Ortholog ofS. cerevisiae: YCR087C-A, C. glabrata CBS138: CAGL0E00517g, C. dubliniensis CD36: Cd36_35524, C. parapsilosis CDC317: CPAR2_200040 and C. auris B8441: B9J08_001263
C5_02810W_A	orf19.4305.1	orf19.4305.1	0.595496453	-0.747835179	0.001217108	Ortholog(s) have RNA binding activity, role in mRNA splicing, via spliceosome and U4/U6 x U5 tri-snRNP complex, U6 snRNP, nucleolus, nucleus localization
C2_04460W_A	orf19.4506	LYS22	0.595247671	-0.748438025	6.6249E-08	Homocitrate synthase, minor isoform; repressed by nitric oxide and by hypoxia; protein level decreases in stationary phase cultures; induced by ketoconazole, Spider biofilm induced; flow model biofilm repressed
CR_04740C_A	orf19.6317	ADE6	0.594814078	-0.749489302	2.28699E-11	5-Phosphoribosylformyl glycinamidine synthetase; adenine biosynthesis; not induced in GCN response, in contrast to S. cerevisiae Ade6; protein in stationary phase yeast-form cultures; flow model and rat catheter biofilm repressed
CR_02980C_A	orf19.2859	SRP40	0.594737466	-0.749675132	5.68139E-05	Putative chaperone of small nucleolar ribonucleoprotein particles; macrophage/pseudohyphal-induced; rat catheter biofilm induced
C5_00200C_A	orf19.978	BDF1	0.594700024	-0.749765962	6.23746E-11	Essential chromatin-binding bromodomain protein; repressed upon adherence to polystyrene; reduced mRNA abundance detected in null mutant; macrophage/pseudohyphal-repressed
C5_02370C_A	orf19.4245	OPY1	0.594636898	-0.749919107	1.11905E-06	Protein with a predicted pleckstrin domain; Hap43-repressed gene; mutation causes decreased interaction with macrophages

C5_00190C_A	orf19.979	FAS1	0.594376575	-0.750550835	6.48584E-12	Beta subunit of fatty-acid synthase; multifunctional enzyme; Hap43, fluconazole-induced; amphotericin B, caspofungin repressed; macrophage/pseudohyphal-induced; flow model and Spider biofilm repressed
CR_00640W_A	orf19.7466	ACC1	0.594336804	-0.750647373	1.32122E-09	Putative acetyl-coenzyme-A carboxylases; regulated by Efg1; amphotericin B repressed; caspofungin repressed; 5'-UTR intron; gene used for strain identification by multilocus sequence typing; Hap43-induced; flow model biofilm repressed
C7_00330C_A	orf19.7088	orf19.7088	0.592964504	-0.75398235	3.92121E-06	Ortholog(s) have N(6)-L-threonylcarbamoyladenine synthase activity, single-stranded telomeric DNA binding activity
C2_02690W_A	orf19.5838	SER2	0.592851029	-0.754258464	1.15243E-10	Ortholog(s) have phosphoserine phosphatase activity and role in L-serine biosynthetic process
C4_05130C_A	orf19.742	ALD6	0.591614729	-0.757270125	0.00045719	Putative aldehyde dehydrogenase; stationary phase enriched protein; expression regulated upon white-opaque switch; rat catheter biofilm induced; rat catheter and Spider biofilm induced
C1_10950C_A	orf19.2320	orf19.2320	0.5915445	-0.757441392	5.54747E-08	Putative serine/threonine-protein kinase; possibly an essential gene, disruptants not obtained by UAU1 method
C3_03440C_A	orf19.341	orf19.341	0.591331817	-0.75796019	1.40617E-08	Putative spermidine export pump; fungal-specific (no human or murine homolog)
C6_02980C_A	orf19.5576	orf19.5576	0.590310362	-0.760454429	3.65592E-10	Putative pantothenate kinase; ortholog of S. cerevisiae Cab1; transposon mutation affects filamentous growth; repressed in core stress response
C4_02080W_A	orf19.4583	orf19.4583	0.590099391	-0.760970126	2.2924E-05	Protein with a mitochondrial carrier protein domain; possibly an essential gene, disruptants not obtained by UAU1 method; Spider biofilm repressed

C3_06000W_A	orf19.7381	AHR1	0.590012102	-0.761183548	0.000210271	Zn(II)2Cys6 transcription factor; involved in regulation of adhesion genes; involved in white-opaque switch; acts as repressor of START; forms complex with Mcm1; mutant is sensitive to 5-fluorocytosine and lithium chloride
CR_02190C_A	orf19.3753	SEF1	0.589831971	-0.761624071	2.76315E-09	Zn2-Cys6 transcription factor; regulates iron uptake; negatively regulated by Sfu1p, positively regulated by Tbf1; promotes virulence in mice; mutants display decreased colonization of mouse kidneys; Spider biofilm induced
C1_10450W_A	orf19.986	GLY1	0.589802888	-0.761695208	1.72847E-06	L-threonine aldolase; complements glycine auxotrophy of S. cerevisiae shm1 shm2 gly1-1 triple mutant; macrophage/pseudohyphal-induced; the GLY1 locus has an RFLP and is triploid in strain SGY269; flow model biofilm induced
CR_06660W_A	orf19.700	SEO1	0.58893888	-0.763810176	1.04591E-15	Protein with similarity to permeases; Sfu1-repressed; flucytosine induced; induced by Mnl1 under weak acid stress; flow model biofilm repressed
CR_09730C_A	orf19.6589	SSF1	0.588898625	-0.763908789	0.00012239	Protein involved in ribosome biogenesis; ortholog of S. cerevisiae Ssf1; Hap43-induced; rat catheter and Spider biofilm induced
CR_08900C_A	orf19.7285	orf19.7285	0.588244599	-0.765511925	2.59286E-05	Ortholog(s) have role in mRNA polyadenylation, pre-mRNA cleavage required for polyadenylation, regulation of mRNA 3'-end processing, sno(s)RNA 3'-end processing, termination of RNA polymerase II transcription, exosome-dependent
C1_08500C_A	orf19.395	ENO1	0.588124317	-0.765806952	2.59686E-09	Enolase, involved in glycolysis and gluconeogenesis; also has transglutaminase activity involved in assembly of cell

						wall polysaccharides; major cell-surface antigen; binds host plasmin/plasminogen; immunoprotective; may be essential
C5_01750C_A	orf19.3200	MTLA2	0.587409891	-0.767560537	0.034880894	Master regulator (activator) of a-type mating; has HMG domain, which is predicted to bind DNA; gene has intron; not related to S. cerevisiae MATa2; a/alpha mating type may increase virulence, provides competitive advantage
C5_04280C_A	orf19.3912	GLN3	0.587358107	-0.767687725	4.14979E-10	GATA transcription factor, involved in regulation of nitrogen starvation-induced filamentous growth; regulates transcription of Mep2 ammonium permease; regulated by Gcn2 and Gcn4; mRNA binds She3; Spider biofilm induced
C4_04410C_A	orf19.3846	LYS4	0.587348601	-0.767711074	7.12437E-11	Homoaconitase; regulated by Gcn4, Gcn2; induced in response to amino acid starvation (3-AT); induced by human whole blood or PMNs; Hap43-repressed; flow model and Spider biofilm repressed
C1_01490W_A	orf19.3335	orf19.3335	0.586703564	-0.769296338	3.48792E-09	Plasma membrane protein of unknown function; colony morphology-related gene regulation by Ssn6; repressed by nitric oxide
C1_01450W_A	orf19.3331	ABC1	0.586669397	-0.769380358	2.53105E-11	Putative ubiquinol-cytochrome-c reductase; induced upon adherence to polystyrene; flow model biofilm induced; Spider biofilm induced
CR_01580C_A	orf19.2545	DOT6	0.585992298	-0.771046392	3.27005E-11	Protein with a predicted role in telomeric gene silencing and filamentation; repressed by high-level peroxide stress; Spider biofilm induced
C6_02500C_A	orf19.5519	GCV1	0.585923687	-0.771215321	1.14291E-06	Putative T subunit of glycine decarboxylase; transcript negatively regulated by Sfu1; Spider biofilm repressed
C1_09540W_A	orf19.4818	orf19.4818	0.585217555	-0.772955047	1.79501E-08	Protein of unknown function; Spider biofilm induced

C1_05220C_A	orf19.444	orf19.444	0.584310797	-0.775192148	0.00476568	Has domain(s) with predicted nucleic acid binding, nucleotide binding activity
C1_12800W_A	orf19.6342	orf19.6342	0.581047658	-0.783271595	0.003042834	Ortholog of C. dubliniensis CD36: Cd36_11980, C. parapsilosis CDC317: CPAR2_201330, C. auris B8441: B9J08_003701 and Candida tenuis NRRL Y-1498: CANTEDRAFT_114815
C2_10160W_A	orf19.1765	orf19.1765	0.580941241	-0.783535845	1.77918E-05	Secreted protein; fluconazole-induced
CR_10590W_A	orf19.7642	orf19.7642	0.580833578	-0.783803238	4.98422E-09	Ortholog of S. cerevisiae Vps3; CORVET tethering complex component involved in vacuolar protein sorting; Hap43-repressed gene
CR_10640W_A	orf19.7648	orf19.7648	0.580713918	-0.784100483	1.78636E-05	Has domain(s) with predicted antiporter activity, xenobiotic transmembrane transporter activity, role in xenobiotic transmembrane transport and membrane localization
C4_04730W_A	orf19.3809	BAS1	0.580152099	-0.785496913	7.61914E-08	Putative Myb-like transcription factor; ortholog S. cerevisiae Bas1, a regulator of purine biosynthetic genes; mutant exhibits adenine auxotrophy and abnormal colony morphology
C7_03850W_A	orf19.7197	orf19.7197	0.579136717	-0.788024129	3.25032E-08	Putative intranuclear transport and DNA replication mediator; heterozygous null mutant exhibits resistance to parnafungin in the C. albicans fitness test; Spider biofilm induced
C5_02080C_A	orf19.3160	HSP12	0.57832207	-0.790054934	0.005201082	Heat-shock protein; induced by osmotic/oxidative/cadmium stress, fluphenazine treatment, low iron, CDR1 and CDR2 overexpression, or ssn6 or ssk1 null mutation; overexpression increases resistance to farnesol and azoles
C5_02110W_A	orf19.4216	orf19.4216	0.57832207	-0.790054934	0.005201082	Putative heat shock protein; decreased expression in hyphae; transcription is increased in populations of cells

						exposed to fluconazole over multiple generations; overexpression increases resistance to farnesol and azoles
C1_00170W_A	orf19.6086	LEU4	0.577917013	-0.791065754	8.49863E-07	Putative 2-isopropylmalate synthase; involved in resistance to caspofungin and anidulafungin; regulated by NRG1, MIG1, TUP1, GCN4; induced by human whole blood or PMNs; macrophage/pseudohyphal-repressed after 16h; Spider biofilm repressed
C4_04880W_A	orf19.3791	FGR10	0.577839146	-0.791260152	3.59584E-05	Putative asparaginase; lacks ortholog in S. cerevisiae; transposon mutation affects filamentous growth; Spider biofilm induced
C3_05950W_A	orf19.7376	orf19.7376	0.576189612	-0.795384443	7.78318E-10	Ortholog of C. dubliniensis CD36: Cd36_85880, C. parapsilosis CDC317: CPAR2_806870, C. auris B8441: B9J08_002007 and Candida tenuis NRRL Y-1498: CANTEDRAFT_96983
CR_10470C_A	orf19.7624	orf19.7624	0.576023947	-0.795799304	4.38739E-09	Ortholog(s) have role in maturation of SSU-rRNA from tricistronic rRNA transcript (SSU-rRNA, 5.8S rRNA, LSU-rRNA), rRNA processing and nucleolus, small-subunit processome localization
C1_09970C_A	orf19.4863	PDC2	0.575335392	-0.797524873	6.27086E-11	Homeodomain-like transcription factor; regulator of pyruvate decarboxylase; contains a putative C-terminal activation domain, Glu- and Pro-rich; complements glucose utilization defect of S. cerevisiae pdc2 mutant
CR_02530W_A	orf19.171	DBP2	0.573663551	-0.801723239	7.75608E-10	Putative DEAD-box family ATP-dependent RNA helicase; flucytosine induced; repressed in core stress response
CR_10140W_A	orf19.7590	orf19.7590	0.573652681	-0.801750577	2.37457E-09	Putative NADH-ubiquinone oxidoreductase; identified in detergent-resistant membrane fraction (possible lipid raft component); predicted N-terminal acetylation; repressed by nitric oxide

C4_07090C_A	orf19.3099	TRP4	0.572662099	-0.804243972	9.86175E-12	Predicted enzyme of amino acid biosynthesis; upregulated in biofilm; regulated by Gcn2p and Gcn4p; S. cerevisiae ortholog is Gcn4p regulated
C3_05100C_A	orf19.5982	RPL18	0.572575964	-0.804460986	2.70011E-12	Predicted ribosomal protein; Plc1p-regulated, Tbf1-activated; repressed upon phagocytosis by murine macrophage; Hap43p-induced; Spider biofilm repressed
C1_05010C_A	orf19.55	orf19.55	0.572384766	-0.80494282	8.76263E-07	Ortholog(s) have role in cellular zinc ion homeostasis
C2_02980C_A	orf19.5805	DLD1	0.572219393	-0.805359702	1.41324E-12	Putative D-lactate dehydrogenase; white cell-specific trancript; colony morphology-related gene regulation by Ssn6; Hap43-repressed; rat catheter biofilm induced; Spider biofilm repressed
C1_04370C_A	orf19.5201	orf19.5201	0.57160115	-0.806919275	1.55646E-08	Ortholog(s) have structural constituent of ribosome activity and mitochondrial small ribosomal subunit localization
CR_10490W_A	orf19.7626	EIF4E	0.571407006	-0.807409371	1.20683E-08	Translation initiation factor eIF4E; genes encoding ribosomal subunits, translation factors, tRNA synthetases downregulated by phagocytosis by macrophage; alternatively spliced intron in 5' UTR; protein levels decrease in stationary phase
CR_02580W_A	orf19.163	PAN6	0.571025658	-0.808372522	0.000175197	Ortholog(s) have pantoate-beta-alanine ligase activity and role in pantothenate biosynthetic process
C5_05080W_A	orf19.4000	GRF10	0.570678675	-0.80924944	8.55457E-07	Putative homeodomain transcription factor, involved in copper homeostasis and control of filamentous growth; null mutant is an adenine auxotroph and shows increased copper resistance; promoter bound by Bcr1, Tec1, Efg1, Ndt80 and Brg1
C5_04840C_A	orf19.3970	orf19.3970	0.570348862	-0.81008346	5.53293E-07	Putative ribosome biogenesis factor; possibly essential, disruptants not obtained by UAU1 method; rat catheter and Spider biofilm induced

C1_08190C_A	orf19.5097	CAT8	0.569302614	-0.81273237	1.36552E-13	Zn(II)2Cys6 transcription factor; similar to S. cerevisiae Cat8 but mutant phenotype suggests different target genes; mutant displays increased filamentous/invasive growth; flucytosine repressed; rat catheter biofilm induced
C3_03420C_A	orf19.339	NDE1	0.56908191	-0.813291775	1.33605E-15	Putative NADH dehydrogenase; may act alternatively to complex I in respiration; caspofungin repressed; rat catheter biofilm induced; Spider biofilm repressed
C6_04240W_A	orf19.1082	orf19.1082	0.568945794	-0.813636887	0.000100232	Protein with an Alba DNA/RNA-binding protein domain; Spider biofilm induced
CR_06770C_A	orf19.1857	orf19.1857	0.568871894	-0.81382429	5.80174E-13	Putative L-azetidine-2-carboxylic acid acetyltransferase; mutants are viable
CR_10820W_A	orf19.7673	orf19.7673	0.568660979	-0.814359282	4.7579E-05	Ortholog(s) have mRNA binding activity, role in mRNA splicing, via spliceosome and U1 snRNP, U2-type prespliceosome, U4/U6 x U5 tri-snRNP complex, U5 snRNP, commitment complex, cytosol, nucleus localization
C3_01300C_A	orf19.1721	NCE103	0.568570121	-0.814589809	8.53282E-10	Carbonic anhydrase; converts of CO2 to bicarbonate; essential for virulence in host niches with limited CO2, normal white-opaque switch; Mnl1-induced in weak acid stress; Hap43-induced gene; F-12/CO2, rat catheter, Spider biofilm induced
C5_05220W_A	orf19.4015	CAG1	0.568562438	-0.814609304	9.64093E-09	Heterotrimeric G protein alpha subunit; positive role in mating pheromone response; opaque-enriched transcript; transcript repressed by MTLa1-MTLalpha2; regulated by hemoglobin-responsive Hbr1 via MTL genes; rat catheter biofilm repressed
C4_04300C_A	orf19.1417	orf19.1417	0.568548183	-0.814645476	0.00125995	Ortholog of C. dubliniensis CD36: Cd36_43970, C. parapsilosis CDC317: CPAR2_401720, C. auris B8441:

						B9J08_000295 and Candida tenuis NRRL Y-1498: CANTEDRAFT_102588
CR_06760C_A	orf19.1860	LSC2	0.56837834	-0.815076518	8.66977E-13	Putative succinate-CoA ligase beta subunit; regulated by Mig1, Tup1; induced in high iron; protein present in exponential and stationary growth phase yeast cells; Spider biofilm repressed
C2_10240W_A	orf19.1756	GPD1	0.567828419	-0.816473039	3.28767E-10	Glycerol-3-phosphate dehydrogenase; glycerol biosynthesis; regulated by Efg1; regulated by Tsa1, Tsa1B under H2O2 stress conditions; Sflow model and Spider biofilm induced
CR_04600W_A	orf19.551	orf19.551	0.567682363	-0.816844174	1.4686E-17	Ortholog of C. dubliniensis CD36: Cd36_29980, C. parapsilosis CDC317: CPAR2_204170, C. auris B8441: B9J08_004836 and Candida tenuis NRRL Y-1498: CANTEDRAFT_110235
C1_09640W_A	orf19.4827	ADE12	0.567321076	-0.817762633	1.63784E-11	Adenylosuccinate synthase; upregulated in biofilm; decreased expression in hyphae vs yeast-form cells; not induced during GCN response, in contrast to S. cerevisiae ADE12, which is induced by Gcn4p; stationary phase-enriched protein
CR_10670W_A	orf19.7654	CPR6	0.566323378	-0.82030201	8.18212E-10	Putative peptidyl-prolyl cis-trans isomerase; macrophage/pseudohyphal-repressed; heavy metal (cadmium) stress-induced; heterozygous null mutant displays sensitivity to virgineone; rat catheter biofilm induced
CR_07010W_A	orf19.2364	MIS11	0.566049432	-0.82100005	1.40914E-12	Predicted mitochondrial C1-tetrahydrofolate synthase precursor; putative protein of glycine catabolism; repressed by Efg1; fluconazole-induced; stationary phase enriched protein; rat catheter and Spider biofilm repressed

CR_02000C_A	orf19.2601	HEM1	0.565939867	-0.821279324	3.74585E-12	Putative 5-aminolevulinate synthase; caspofungin repressed; induced by high iron, nitric oxide; regulated by Ssn6; Hap43-repressed; Spider biofilm induced
CR_09910W_A	orf19.7565	GNP3	0.565310818	-0.82288379	2.86346E-07	Putative high-affinity glutamine permease; fungal-specific (no human or murine homolog)
CR_09240C_A	orf19.7322	orf19.7322	0.565305725	-0.822896787	1.42304E-06	Protein of unknown function; S. cerevisae ortholog Ypl225w interacts with ribosomes; rat catheter biofilm induced
C4_01220C_A	orf19.4668	orf19.4668	0.565008405	-0.823655766	1.41877E-08	Protein with a glycoside hydrolase domain; mutants are viable
C3_02840W_A	orf19.275	orf19.275	0.564418315	-0.825163291	7.50724E-05	Ortholog(s) have RNA binding, ribonuclease MRP activity, ribonuclease P activity
C1_10110W_A	orf19.4880	orf19.4880	0.564415856	-0.825169578	4.66132E-06	Protein of unknown function; flow model biofilm induced
C3_04990W_A	orf19.5967	FGR44	0.563240488	-0.828177051	8.62092E-06	Protein lacking an ortholog in S. cerevisiae; transposon mutation affects filamentous growth
C3_04830C_A	orf19.5949	FAS2	0.562883661	-0.829091324	1.36771E-19	Alpha subunit of fatty-acid synthase; required for virulence in mouse systemic infection and rat oropharyngeal infection models; regulated by Efg1; fluconazole-induced; amphotericin B repressed; flow model and Spider biofilm repressed
C4_00760W_A	orf19.4166	ZCF21	0.561332909	-0.833071453	7.55652E-05	Predicted Zn(II)2Cys6 transcription factor; mutants display increased colonization of mouse kidneys; Spider biofilm induced
CR_04090C_A	orf19.492	ADE17	0.560634529	-0.834867494	2.4874E-12	5-Aminoimidazole-4-carboxamide ribotide transformylase, enzyme of adenine biosynthesis; antigenic in human; soluble protein in hyphae; not induced during GCN response, in contrast to the S. cerevisiae ortholog

CR_09660W_A	orf19.6597	orf19.6597	0.560533739	-0.835126882	1.95099E-05	Ortholog ofS. cerevisiae: YLR287C, C. glabrata CBS138: CAGL0M09757g, C. dubliniensis CD36: Cd36_34830, C. parapsilosis CDC317: CPAR2_701110 and C. auris B8441: B9J08_000560
C5_03410C_A	orf19.2639	orf19.2639	0.560519967	-0.835162329	3.56924E-05	Ortholog(s) have structural constituent of ribosome activity and mitochondrial large ribosomal subunit localization
CR_06480C_A	orf19.718	RRN11	0.558937658	-0.839240716	7.08073E-07	Putative RNA polymerase I subunit; rat catheter biofilm induced; Spider biofilm induced
CR_10850C_A	orf19.7678	ATP16	0.558172521	-0.841216994	8.02275E-13	Subunit of the mitochondrial F1F0 ATP synthase; sumoylation target; protein newly produced during adaptation to the serum; Spider biofilm repressed
C4_07150W_A	orf19.3088	orf19.3088	0.557950832	-0.8417901	0.020954897	bZIP transcription factor; possibly transcriptionally regulated upon hyphal formation; Hap43; F-12/CO2 early biofilm induced; Spider biofilm induced
C2_06210C_A	orf19.5484	SER1	0.55765594	-0.842552807	1.13145E-11	Putative 3-phosphoserine aminotransferase; predicted role in serine and glycine biosynthesis; protein present in exponential and stationary yeast growth phases; Spider biofilm repressed
C4_04720W_A	orf19.3810	orf19.3810	0.557355411	-0.843330505	5.78817E-16	Ortholog(s) have methylenetetrahydrofolate dehydrogenase (NAD+) activity, role in folic acid-containing compound biosynthetic process, one-carbon metabolic process, purine nucleobase biosynthetic process and cytosol localization
C3_05730C_A	orf19.7354	LAC1	0.556215822	-0.84628331	9.83357E-09	Ceramide synthase; required for biosynthesis of ceramides with C18:0 fatty acids, which serve as precursors for glucosylsphingolipids; caspofungin induced
C1_08000W_A	orf19.5071	NRP1	0.555930743	-0.847022929	3.32915E-16	Ortholog(s) have cytoplasmic stress granule localization
CR_00810W_A	orf19.3275	orf19.3275	0.555785294	-0.847400433	0.000157332	Ortholog of C. dubliniensis CD36: Cd36_25870, C. parapsilosis CDC317: CPAR2_804000, C. auris B8441:

						B9J08_001023 and Candida tenuis NRRL Y-1498: CANTEDRAFT_115661
CR_09770C_A	orf19.7549	PMT5	0.554925964	-0.849632789	5.99502E-15	Protein mannosyltransferase (PMT), expressed at extremely low levels; not required for wild-type hyphal growth, drug resistance, or virulence in mouse systemic infection; one of five PMT family members
C1_05960W_A	orf19.2451	PGA45	0.5545807	-0.850530686	8.75706E-09	Putative GPI-anchored cell wall protein; repressed in core caspofungin response; Hog1-induced; regulated by Ssn6; Mob2-dependent hyphal regulation; flow model biofilm induced
C5_02540C_A	orf19.4266	SPR28	0.553920968	-0.852247944	0.003198696	Septin; similar to S. cerevisiae meiotic/sporulation septin; mutant has no obvious phenotype; two introns with noncanonical branch site and 5' splice site, respectively; splicing inhibited upon exposure to alpha-factor
C4_00810C_A	orf19.4160	orf19.4160	0.553787365	-0.852595957	2.86414E-05	Ortholog(s) have N(6)-L-threonylcarbamoyladenine synthase activity and role in mitochondrial tRNA threonylcarbamoyladenosine modification, tRNA threonylcarbamoyladenosine modification
C3_00920W_A	orf19.6169	ATO1	0.553367367	-0.853690527	4.33033E-05	Putative fungal-specific transmembrane protein; induced by Rgt1; Spider biofilm induced
C4_05540W_A	orf19.1235	HOM3	0.551008253	-0.859854166	1.42839E-11	Putative L-aspartate 4-P-transferase; fungal-specific (no human or murine homolog); regulated by Gcn2 and Gcn4; early-stage flow model biofilm induced
C2_09060C_A	orf19.198	ASN1	0.550636855	-0.86082692	3.66067E-19	Putative asparagine synthetase; soluble protein in hyphae; regulated by Rim101; decreased expression at pH 4 vs pH 8; protein detected during exponential and stationary phases of yeast-form growth

C7_00430W_A	orf19.7077	orf19.7077	0.550003878	-0.862486304	1.2178E-10	Putative ferric reductase; induced by Mac1 under copper starvation; Plc1-regulated; Rim101-repressed
C3_07200C_A	orf19.6781	ZFU2	0.549198194	-0.864601215	5.88444E-05	Zn(II)2Cys6 transcription factor; regulator of yeast form adherence; mutants display increased colonization of mouse kidneys; required for yeast cell adherence to silicone substrate; Spider biofilm induced
CR_09800C_A	orf19.7552	orf19.7552	0.547962957	-0.867849727	6.78177E-08	Putative U3-containing small subunit processome complex protein; Hap43-induced gene; repressed in core stress response; Spider biofilm induced
C1_10610W_A	orf19.1832	FCY23	0.547608587	-0.868783026	2.12354E-19	Putative transporter; Gcn4p-regulated; more similar to S. cerevisiae Tpn1p, which is a vitamin B6 transporter, than to purine-cytosine permeases
C4_06430C_A	orf19.2891	orf19.2891	0.546214289	-0.872461039	1.25783E-08	Ortholog(s) have role in cellular response to oxidative stress, protein quality control for misfolded or incompletely synthesized proteins and mitochondrial inner membrane localization
C7_01730C_A	orf19.6551.1	STE18	0.546213798	-0.872462337	0.001156172	Protein similar to S. cerevisiae Ste18p; expressed in opaque or white MTLa/MTLa or MTLalpha/MTLalpha, but not MTLa/MTLalpha cells; MTLa1p, MTLalpha2p bind promoter region
C1_10320W_A	orf19.4903	orf19.4903	0.543692667	-0.879136726	8.36451E-08	Ortholog(s) have N-acetylglucosaminylphosphatidylinositol deacetylase activity
CR_10250C_A	orf19.7600	FDH3	0.540970718	-0.88637759	1.97794E-24	Glutathione-dependent formaldehyde dehydrogenase; glycine catabolism; repressed by Efg1 in yeast, not hyphal growth conditions; induced by Mnl1 under weak acid stress; Spider biofilm repressed

C2_03610W_A	orf19.857	FMO2	0.539744296	-0.889652004	2.5977E-05	Protein with a monooxygenase domain; Spider biofilm induced
C3_01710C_A	orf19.1673	PPT1	0.539348297	-0.890710866	2.1185E-10	Putative serine/threonine phosphatase; induced in high iron
C4_04230W_A	orf19.1427	orf19.1427	0.539182964	-0.89115318	1.79394E-08	Putative transporter; fungal-specific; Spider biofilm induced
C3_00030C_A	orf19.5469	orf19.5469	0.538204558	-0.893773487	1.07466E-14	Protein with a predicted DEAD-like DNA/RNA helicase domain; shows colony morphology-related gene regulation by Ssn6; overlaps orf19.5472; Spider biofilm repressed
CR_10370W_A	orf19.7613	HCR1	0.537784283	-0.894900502	8.55945E-18	Putative translation initiation factor; repressed upon phagocytosis by murine macrophage; Spider biofilm repressed
C4_04160W_A	orf19.5293	orf19.5293	0.537564813	-0.895489386	1.70351E-11	Ortholog of C. dubliniensis CD36: Cd36_43710, C. parapsilosis CDC317: CPAR2_402940, C. auris B8441: B9J08_005206 and Candida tenuis NRRL Y-1498: CANTEDRAFT_114940
CR_00510C_A	orf19.7484	ADE1	0.537041693	-0.896894	2.82066E-14	Phosphoribosylaminoimadazole succinocarboxamide synthetase, enzyme of adenine biosynthesis; not induced in GCN response, unlike the S. cerevisiae ortholog; fungal-specific (no human or murine homolog); levels decrease in stationary phase
C2_05380W_A	orf19.3569	orf19.3569	0.534703318	-0.903189465	1.12213E-08	Protein of unknown function; S. cerevisiae YLR407W mutants have abnormal budding; constitutive expression independent of MTL or white-opaque status; Spider biofilm induced
CR_08930C_A	orf19.7290	orf19.7290	0.534586766	-0.903503972	9.10207E-20	Ortholog(s) have RNA cap binding, chromatin binding, mRNA binding activity and role in deadenylation-dependent decapping of nuclear-transcribed mRNA,

						nuclear-transcribed mRNA catabolic process, deadenylation-dependent decay
C3_06520C_A	orf19.7441	orf19.7441	0.53444533	-0.903885716	2.37064E-11	Ortholog(s) have role in lipid homeostasis, nuclear envelope organization and nuclear envelope localization
CR_01320C_A	orf19.3222	orf19.3222	0.532840048	-0.908225575	5.89956E-18	Predicted vacuolar protein; rat catheter biofilm repressed; flow model biofilm repressed
C3_03040W_A	orf19.300	AIP2	0.532020534	-0.910446164	3.4591E-14	Putative actin interacting protein; regulated by Gcn4; induced in response to amino acid starvation (3-AT); repressed by elevated CO2; flow model biofilm repressed
C1_11030W_A	orf19.2311	RPL82	0.531590698	-0.911612235	2.68954E-14	Predicted ribosomal protein; genes encoding cytoplasmic ribosomal subunits, translation factors, and tRNA synthetases are downregulated upon phagocytosis by murine macrophage
CR_08860W_A	orf19.7281	PDK2	0.531106421	-0.912927124	1.36807E-15	Putative pyruvate dehydrogenase kinase; mutation confers hypersensitivity to amphotericin B
CR_10540C_A	orf19.7634	MCD1	0.530527182	-0.914501424	7.86703E-14	Alpha-kleisin cohesin complex subunit; for sister chromatid cohesion in mitosis and meiosis; repressed by alpha pheromone in SpiderM medium; periodic cell-cycle expression; Hap43-repressed; rat catheter and Spider biofilm repressed
CR_09830W_A	orf19.7554	orf19.7554	0.530157197	-0.915507898	4.22758E-08	Transporter; similar to the Sit1 siderophore transporter; induced by nitric oxide independent of Yhb1; repressed during chlamydospore formation in C. albicans and C. dubliniensis; rat catheter biofilm repressed
CR_09670C_A	orf19.6596	orf19.6596	0.52854201	-0.919909949	1.08357E-09	Putative esterase; possibly transcriptionally regulated by Tac1; induced by Mnl1 under weak acid stress; protein present in exponential and stationary growth phase yeast cultures; Spider biofilm repressed

C1_05940W_A	orf19.2454	PHO87	0.527357908	-0.923145671	5.76159E-16	Putative phosphate permease; transcript repressed by Rim101 at pH 8; regulated by white-opaque switch; caspofungin repressed; virulence-group-correlated expression; flow model biofilm induced
C2_06590C_A	orf19.34	GIT1	0.526080571	-0.926644324	4.69301E-07	Glycerophosphoinositol permease; involved in utilization of glycerophosphoinositol as a phosphate source; Rim101-repressed; virulence-group-correlated expression
C1_11990W_A	orf19.5267	CWP419	0.525894382	-0.927155011	6.32287E-07	Cell wall adhesin-like protein involved in regulation of covering (masking) cell wall glucan; repressed in core caspofungin response and by alpha pheromone in SpiderM medium; transcript reduced in ace2 mutant
C7_03300C_A	orf19.1345	LIP8	0.525471822	-0.928314692	8.71812E-10	Secreted lipase, member of a differentially expressed lipase gene family with possible roles in nutrition and/or in creating an acidic microenvironment; LIP5 and LIP8 are expressed at all stages of both mucosal and systemic infection
C6_02380W_A	orf19.3481	orf19.3481	0.522932951	-0.935302116	4.11404E-18	Putative mitochondrial ATP-dependent RNA helicase of the DEAD-box family, transcription is activated in the presence of elevated CO2
C1_07890C_A	orf19.5061	ADE5	0.52234552	-0.936923662	1.00425E-23	Phosphoribosylamine-glycine ligase and phosphoribosylformylglycinamidine cyclo-ligase; interacts with Vps34p; required for hyphal growth and virulence; flucytosine induced; not induced in GCN response, in contrast to S. cerevisiae ortholog
CR_10060W_A	orf19.7581	orf19.7581	0.522245956	-0.937198678	1.31682E-14	Protein with a predicted role in assembly of U2 snRNP into the spliceosome; Spider biofilm induced
CR_10160W_A	orf19.7592	FAA4	0.521729798	-0.938625261	3.09548E-25	Acyl CoA synthase involved in uptake of long-chain fatty acids and biofilm formation

CR_08340W_A	orf19.6402	CYS3	0.52123129	-0.940004403	6.51242E-17	Cystathionine gamma-lyase; induced by alkaline, amphotericin B, cadmium stress, oxidative stress via Cap1; possibly adherence-induced; Hog1 regulated; reduced levels in stationary phase yeast cells; Spider and flow model biofilm induced
CR_10270C_A	orf19.7602	AHA1	0.519679219	-0.944306726	4.00739E-17	Putative Hsp90p co-chaperone; Hap43-repressed; heavy metal (cadmium) stress-induced; oxidative stress-induced via Cap1; rat catheter biofilm induced; flow model biofilm repressed
C2_03640W_A	orf19.854	UGA11	0.519634314	-0.944431392	9.707E-09	Putative gamma-aminobutyrate (GABA) transaminase; macrophage-induced; overlaps orf19.854.1, which is a region annotated as a blocked reading frame; Spider biofilm induced
C1_00010W_A	orf19.6115	orf19.6115	0.519061115	-0.946023681	3.49862E-05	Dubious open reading frame
C4_06450W_A	orf19.2889	orf19.2889	0.519055681	-0.946038785	0.012221814	Ortholog(s) have role in ATP-dependent chromatin remodeling, chromatin remodeling, histone exchange and positive regulation of cellular response to phosphate starvation, more
CR_10120C_A	orf19.7588	orf19.7588	0.518594162	-0.947322131	0.004290064	Ortholog ofS. cerevisiae: RRG7, C. glabrata CBS138: CAGL0I09680g, C. dubliniensis CD36: Cd36_35140, C. parapsilosis CDC317: CPAR2_200670 and Candida tenuis NRRL Y-1498: CANTEDRAFT_117734
CR_09010C_A	orf19.7297	orf19.7297	0.514466038	-0.958852252	2.10406E-24	Putative cystathionine gamma-synthase; decreased levels in stationary phase cultures; Hog1p-induced; Gcn4p-regulated
C2_03270W_A	orf19.903	GPM1	0.511418684	-0.967423228	1.63401E-14	Phosphoglycerate mutase; surface protein that binds host complement Factor H and FHL-1; antigenic; fluconazole, or amino acid starvation (3-AT) induced, farnesol-

						repressed; Hap43, flow model biofilm induced; Spider biofilm repressed
C5_03060C_A	orf19.4335	TNA1	0.508654683	-0.975241527	5.0359E-07	Putative nicotinic acid transporter; detected at germ tube plasma membrane by mass spectrometry; transcript induced upon phagocytosis by macrophage; rat catheter biofilm induced
CR_10580W_A	orf19.7638	PRO1	0.508525031	-0.975609306	9.59884E-24	Putative gamma-glutamyl kinase; transcript regulated by Nrg1; regulated by Gcn2 and Gcn4; Hap43-repressed gene; early-stage flow model biofilm induced gene
C4_06220C_A	orf19.2916	orf19.2916	0.506550928	-0.981220771	0.017251944	Ortholog of C. dubliniensis CD36: Cd36_45740, C. parapsilosis CDC317: CPAR2_401900, Candida tenuis NRRL Y-1498: CANTEDRAFT_116046 andPichia stipitis Pignal: PICST_28890
CR_09320C_A	orf19.7327	PHO88	0.505938119	-0.982967153	1.06598E-19	Protein with a role in phosphate transport; biofilm-regulated expression; amphotericin B repressed
C4_02440C_A	orf19.2758	PGA38	0.505642879	-0.983809283	2.64078E-11	Putative adhesin-like GPI-anchored protein; repressed during cell wall regeneration; possibly an essential gene, disruptants not obtained by UAU1 method; rat catheter and Spider biofilm repressed
C4_06820C_A	orf19.3127	CZF1	0.505098265	-0.985364008	5.8186E-05	Transcription factor; regulates white-opaque switch; hyphal growth regulator; expression in S. cerevisiae causes dominant-negative inhibition of pheromone response; required for yeast cell adherence to silicone; Spider biofilm induced
C4_06530C_A	orf19.2882	XUT1	0.501623217	-0.995323971	3.88353E-06	Putative high-affinity, high-capacity xanthine-uric acid/H+ symporter; similar to A. nidulans UapA; member of the Nucleobase-Ascorbate Transporter/Nucleobase-Cation

						Symporter (NAT/NCS2) family; rat catheter biofilm induced
CR_10240W_A	orf19.7599	UTP5	0.500579778	-0.998328083	1.88196E-15	Putative U3 snoRNA-associated protein; Hap43p-induced gene; mutation confers resistance to 5-fluorocytosine (5-FC), 5-fluorouracil (5-FU), and tubercidin (7-deazaadenosine); physically interacts with TAP-tagged Nop1p
C1_02980W_A	orf19.2989	GOR1	0.500426239	-0.998770658	1.89774E-12	Ortholog(s) have glyoxylate reductase (NAD+) activity, role in glyoxylate catabolic process and extracellular region localization
C5_01010W_A	orf19.1969	CCW14	0.498915774	-1.003131813	2.2553E-12	Putative mannoprotein of cell wall with role in response to stress; increased mRNA abundance observed in cyr1 homozygous mutant (hyphal or yeast-form cells) and in ras1 homozygous mutant (yeast-form cells)
C6_00790C_A	orf19.3646	CTR1	0.497809478	-1.006334397	4.11063E-24	Copper transporter; transcribed in low copper; induced Mac1, Tye7, macrophage interaction, alkaline pH via Rim101; 17-beta-estradiol repressed; complements S. cerevisiae ctr1 ctr3 copper transport mutant; flow model/Spider biofilm induced
C5_04850W_A	orf19.3971	orf19.3971	0.49689938	-1.008974354	0.000173571	Protein of unknown function; early-stage flow model biofilm induced
CR_09740W_A	orf19.7546	orf19.7546	0.495341399	-1.013504893	3.80856E-12	Protein involved in rRNA processing; required for maturation of the 35S primary transcript of pre-rRNA and for cleavage leading to mature 18S rRNA; Spider biofilm induced
C4_06760W_A	orf19.3133	GUT2	0.493552344	-1.018724996	1.60196E-14	Glycerol-3-phosphate dehydrogenase; Plc1p-regulated; rat catheter biofilm induced; Spider biofilm induced

C1_05950C_A	orf19.2452	orf19.2452	0.4914375	-1.024920146	1.38463E-12	Protein of unknown function; induced in high iron; repressed in core caspofungin response; ketoconazole-repressed; colony morphology-related gene regulation by Ssn6; possibly subject to Kex2 processing
CR_10010C_A	orf19.7577	MSS51	0.49038586	-1.028010716	8.14735E-21	Putative mRNA maturation factor; fungal-specific (no human or murine homolog)
CR_10730C_A	orf19.7662	orf19.7662	0.490076317	-1.028921665	8.62063E-09	Ortholog(s) have RNA polymerase II complex binding activity, role in DNA damage response, detection of DNA damage, mRNA 3'-end processing, negative regulation of transposition, RNA-mediated and site of double-strand break localization
C2_01060C_A	orf19.2018	orf19.2018	0.489895908	-1.029452855	9.74232E-08	Protein with a predicted DnaJ chaperone domain and a CSL-type zinc finger; Spider biofilm induced
C2_01050W_A	orf19.2018.2	orf19.2018.2	0.487769012	-1.035729989	0.017251944	Ortholog of Candida tropicalis NEW ASSEMBLY: CTRG1_CGOB_00043 and Candida tropicalis MYA-3404: CTRG_04298
CR_09370W_A	orf19.7332	ELF1	0.487678192	-1.035998636	2.42186E-13	Putative mRNA export protein; Walker A and B (ATP/GTP binding) motifs; required for wild-type morphology, growth; expressed in hyphal, pseudohyphal, and yeast form; Hap43-induced; Spider and flow model biofilm induced
CR_10650W_A	orf19.7650	LTV1	0.487157206	-1.037540688	6.93596E-08	Putative GSE complex component; repressed by prostaglandins
C5_04110W_A	orf19.3893	SCW11	0.48524519	-1.043214183	2.97847E-17	Cell wall protein; repressed in ace2 mutant; repressed in core caspofungin response; induced in high iron; possibly an essential gene, disruptants not obtained by UAU1 method; rat catheter and Spider biofilm repressed

CR_09330C_A	orf19.7328	orf19.7328	0.484661965	-1.044949226	4.03641E-20	Protein with a Staphylococcal nuclease domain; transcript regulated by Mig1 and Tup1; flow model and Spider biofilm repressed
C5_01020C_A	orf19.1968.1	orf19.1968.1	0.484077002	-1.04669154	0.005443402	Predicted non-catalytic subunit of N-terminal acetyltransferase; Spider biofilm induced
C3_05900W_A	orf19.7370	orf19.7370	0.483006373	-1.049885871	4.7715E-16	Possible G-protein coupled receptor; vacuolar membrane transporter for cationic amino acids; PQ-loop motif; rat catheter and Spider biofilm induced
C2_06970W_A	orf19.2251	AAH1	0.481579009	-1.054155584	4.67442E-07	Adenine deaminase; purine salvage and nitrogen catabolism; colony morphology-related regulation by Ssn6; Hog1, CO2-induced; chlamydospore formation repressed in C. albicans and C. dubliniensis; rat catheter and F-12/CO2 biofilm induced
C2_02950W_A	orf19.5809	BNA31	0.481214347	-1.055248439	6.89526E-31	Putative arylformamidase, enzyme of the NAD biosynthesis pathway; Gcn4p-regulated
CR_08940W_A	orf19.7291	orf19.7291	0.480924954	-1.05611631	2.27781E-15	Ortholog(s) have tRNA (adenine-N1-)-methyltransferase activity, role in tRNA methylation and nucleus, tRNA (m1A) methyltransferase complex localization
C3_03020W_A	orf19.298	orf19.298	0.480913199	-1.056151572	0.000783857	Protein of unknown function; flow model biofilm induced
CR_09510C_A	orf19.6612	orf19.6612	0.480020067	-1.058833375	0.002007319	Putative mitochondrial protein; Hap43p-induced gene
C1_13680C_A	orf19.5006	GCV3	0.479306889	-1.060978419	8.94696E-14	Glycine decarboxylase, subunit H; protein level decrease in stationary phase cultures
CR_10410C_A	orf19.7618	orf19.7618	0.477746511	-1.065682757	2.68683E-09	Putative nucleolar protein with a predicted role in pre-18S rRNA processing; Plc1p-regulated; Spider biofilm induced
C2_06700W_A	orf19.3152	AMO2	0.477346375	-1.066891592	2.22897E-14	Protein similar to A. niger predicted peroxisomal copper amino oxidase; mutation confers hypersensitivity to toxic ergosterol analog; F-12/CO2 early biofilm induced

C1_13820C_A	orf19.5020	orf19.5020	0.47242328	-1.081848035	0.000132049	Protein of unknown function; Hap43-induced; Spider biofilm induced
C2_01950C_A	orf19.1507	AMN1	0.47175571	-1.083888114	2.68954E-14	Putative negative regulator of exit from mitosis; Plc1-regulated; rat catheter biofilm induced
C1_05340C_A	orf19.431	ZCF2	0.470618494	-1.087370079	1.13174E-09	Zn(II)2Cys6 transcription factor, required for adaptation to reactive sulfur species; regulates sulfite tolerance through expression of SSU1 and CDG1; Hap43-repressed; Spider biofilm induced
CR_10860C_A	orf19.7680	CTA26	0.470035071	-1.08915969	3.73377E-06	Putative transcription factor/activator; Med2 mediator complex ddomain; transcript is upregulated in an RHE model of oral candidiasis; member of a family of telomere-proximal genes; Efg1, Hap43-repressed
C6_02870W_A	orf19.5563	RNH1	0.467509152	-1.096933487	5.33498E-20	Ribonuclease H (RNAse H); hyphal-induced; flucytosine induced; similar to orf19.5564 (see Locus History); possibly essential (UAU1 method); rat catheter biofilm induced; flow model biofilm repressed
CR_06150C_A	orf19.3870	ADE13	0.467366852	-1.09737268	3.00332E-18	Adenylosuccinate lyase; enzyme of adenine biosynthesis; soluble protein in hyphae; not induced during GCN response, in contrast to the S. cerevisiae ortholog; repressed by nitric oxide
CR_09920W_A	orf19.7566	GNP2	0.466283109	-1.100721927	4.93772E-15	High-specificity proline permease; transcript upregulated in clinical strains from HIV+ patients with oral candidiasis; alkaline upregulated by Rim101; rat catheter, Spider and flow model biofilm induced
C1_04840C_A	orf19.758	orf19.758	0.465828015	-1.102130688	0.000346663	Ortholog(s) have signal sequence binding activity, role in vacuolar transport and late endosome localization
C6_00480C_A	orf19.4211	FET31	0.465228165	-1.103989656	1.72653E-13	Putative multicopper oxidase; ketoconazole/caspofungin/amphotericin B repressed;

						Sef1/Sfu1/Hap43 regulated; reports differ if functional homolog of ScFet3; rat catheter and Spider biofilm induced
CR_09340W_A	orf19.7329	orf19.7329	0.465140115	-1.104262727	1.50985E-17	Ortholog(s) have ubiquitin conjugating enzyme activity, ubiquitin-protein transferase activity
C1_12910W_A	orf19.4921.1	orf19.4921.1	0.462645137	-1.112022068	0.008588547	Protein of unknown function; Spider biofilm repressed
C4_05720W_A	orf19.1257	orf19.1257	0.462398034	-1.112792833	8.80864E-09	Ortholog of Candida albicans WO-1: CAWG_03251
CR_06330C_A	orf19.3887	orf19.3887	0.461751581	-1.114811193	2.78254E-14	Ortholog ofS. cerevisiae: YML108W, C. glabrata CBS138: CAGL0J06666g, C. dubliniensis CD36: Cd36_31830, C. parapsilosis CDC317: CPAR2_204870 and C. auris B8441: B9J08_000809
C1_11450C_A	orf19.657	SAM2	0.452824744	-1.142975301	9.84192E-30	S-adenosylmethionine synthetase; localizes to surface of hyphae, not yeast cells; alkaline, Hog1-induced; farnesol-downregulated; F-12/CO2 early biofilm induced; Spider biofilm repressed
C7_01880C_A	orf19.6532	orf19.6532	0.448977233	-1.155285804	4.46325E-05	Ortholog(s) have FAD transmembrane transporter activity, role in FAD transport and mitochondrion localization
C4_07170C_A	orf19.3087.1	orf19.3087.1	0.448056068	-1.158248818	0.003040065	Ortholog of C. dubliniensis CD36: Cd36_54140, Candida tropicalis NEW ASSEMBLY: CTRG1_CGOB_00095 and Candida albicans WO-1: CAWG_04820
C3_02850C_A	orf19.276	orf19.276	0.448012637	-1.158388667	1.59141E-08	Plasma membrane-associated protein; upregulated in an azole-resistant strain that overexpresses MDR1; Hap43-repressed; Spider biofilm induced
C1_11050W_A	orf19.2310	orf19.2310	0.446970258	-1.16174926	1.54394E-07	Predicted single-stranded nucleic acid binding protein; flow model biofilm induced
C6_01500C_A	orf19.3434	TRY5	0.445035572	-1.168007438	3.22021E-15	Zn(II)2Cys6 transcription factor; regulator of yeast form adherence; required for yeast cell adherence to silicone substrate

C2_02610C_A	orf19.1587	HGT20	0.445013792	-1.168078046	1.33605E-15	Putative glucose transporter of the major facilitator superfamily; the C. albicans glucose transporter family comprises 20 members; 12 probable membrane-spanning segments; regulated by Nrg1
C2_00340C_A	orf19.2098	ARO8	0.441926024	-1.178123204	7.68194E-37	Aromatic transaminase of the Ehrlich fusel oil pathway of aromatic alcohol biosynthesis; Rim101 independent alkaline induction; protein abundance affected by URA3 expression in CAI-4 strain; Gcn4-regulated; stationary phase enriched
C7_01170C_A	orf19.6899	orf19.6899	0.441677039	-1.178936262	2.29624E-05	Putative oxidoreductase; mutation confers hypersensitivity to toxic ergosterol analog; rat catheter and Spider biofilm induced
C4_00750C_A	orf19.4167	orf19.4167	0.441251117	-1.180328164	1.33101E-17	Protein of unknown function; Spider biofilm induced
C3_07140C_A	orf19.6786	orf19.6786	0.440233688	-1.183658545	4.9641E-12	Protein of unknown function; flow model biofilm induced
C7_01570C_A	orf19.6569	orf19.6569	0.438307602	-1.189984395	1.17921E-09	Predicted transmembrane transporter; induced during chlamydospore formation in both C. albicans and C. dubliniensis
C1_04930C_A	orf19.751	orf19.751	0.434841839	-1.201437338	1.95369E-12	Ortholog of C. dubliniensis CD36: Cd36_04660, Candida tropicalis NEW ASSEMBLY: CTRG1_04484, Candida tropicalis MYA-3404: CTRG_04484 and Candida albicans WO-1: CAWG_00904
C2_05250C_A	orf19.3554	AAT1	0.432868375	-1.207999692	3.85408E-22	Aspartate aminotransferase; soluble protein in hyphae; macrophage-induced protein; alkaline upregulated; amphotericin B repressed; gene used for strain identification by multilocus sequence typing; farnesol-, Hap43p-induced; GlcNAc-induced
CR_09820C_A	orf19.7555	orf19.7555	0.432565265	-1.209010273	0.005750973	Ortholog of Candida albicans WO-1: CAWG_02247
CR_07900C_A	orf19.609	orf19.609	0.431307018	-1.213212905	0.008459923	Protein of unknown function; white cell specific transcript

C3_06220C_A	orf19.7411	OAC1	0.429922226	-1.217852399	2.07765E-07	Putative mitochondrial inner membrane transporter; rat catheter biofilm induced
C3_02510C_A	orf19.242	SAP8	0.429615836	-1.218880921	1.52862E-05	Secreted aspartyl protease; regulated by growth phase, temperature, white-opaque switch; highly expressed in opaque cells and upon deep epidermal invasion; greater expression in vaginal than oral infection; prominent role in biofilms
CR_07610C_A	orf19.6288	orf19.6288	0.429224016	-1.220197295	0.006232051	Dubious open reading frame
C1_11630C_A	orf19.1156	FUS1	0.429027652	-1.220857457	1.74424E-07	Membrane protein required for mating; ortholog of S. cerevisiae Fus1; transcript induced by Cph1 in cells homozygous for the MTLa locus; alpha factor induced
CR_00300W_A	orf19.7503	CDA2	0.425714438	-1.232042075	3.63513E-05	Putative chitin deacetylase; transcription is positively regulated by Tbf1p
CR_10400W_A	orf19.7617	orf19.7617	0.422297346	-1.243668916	1.12513E-22	Ortholog(s) have peptide alpha-N-acetyltransferase activity, peptide-glutamate-N-acetyltransferase activity, peptide-serine-N-acetyltransferase activity
CR_10380C_A	orf19.7614	orf19.7614	0.418569958	-1.256459326	2.57914E-08	Protein of unknown function; Hap43-induced gene; mutant is viable
C2_00890W_A	orf19.2035	orf19.2035	0.416605262	-1.263247032	1.99709E-06	Ortholog of C. dubliniensis CD36: Cd36_15830, C. parapsilosis CDC317: CPAR2_212930, Candida tropicalis MYA-3404: CTRG_01155, Candida albicans WO-1: CAWG_03868 and Candida metapsilosis: CMET_2287
CR_10100C_A	orf19.7585	INO1	0.413382669	-1.274450189	1.82787E-34	Inositol-1-phosphate synthase; antigenic in human; repressed by farnesol in biofilm or by caspofungin; upstream inositol/choline regulatory element; glycosylation predicted; rat catheter, flow model induced; Spider biofilm repressed

C6_02210W_A	orf19.3461	orf19.3461	0.408576077	-1.291323362	3.31255E-09	Protein of unknown function; oxidative stress-induced via Cap1; induced by alpha pheromone in SpiderM medium
C3_01680C_A	orf19.1676	orf19.1676	0.405813768	-1.301110284	4.04939E-14	Predicted potassium ion transporter; Spider biofilm induced
C1_03680W_A	orf19.3066	ENG1	0.402688139	-1.312265116	1.57749E-42	Endo-1, 3-beta-glucanase; controls exposure of cell wall beta-glucan to host immune system; caspofungin, fluconazole repressed; repressed by alpha pheromone in SpiderM medium; flow model biofilm induced; rat catheter biofilm repressed
C4_00200C_A	orf19.5645	MET15	0.400902532	-1.318676567	4.64061E-31	O-acetylhomoserine O-acetylserine sulfhydrylase; sulfur amino acid synthesis; immunogenic; Hog1, adherence-induced; brown color of mutant in Pb(2+) medium a visual selection; chlamydospore formation induced, F-12/CO2 biofilm induced
C1_08170C_A	orf19.5094	BUL1	0.399380764	-1.324163248	1.62873E-37	Protein similar but not orthologous to S. cerevisiae Bul1; a protein involved in selection of substrates for ubiquitination; mutants are viable; macrophage/pseudohyphal-induced; rat catheter biofilm induced
C3_04350C_A	orf19.5879	orf19.5879	0.396673504	-1.33397606	2.97847E-17	Has domain(s) with predicted oxidoreductase activity and role in metabolic process
C2_00660C_A	orf19.2062	SOD4	0.396338426	-1.335195248	2.24629E-11	Cu-containing superoxide dismutase; role in response to host innate immune ROS; regulated on white-opaque switch; induced under iron starvation; ciclopirox olamine induced; caspofungin repressed; SOD1, 4, 5, 6 gene family
C6_02080W_A	orf19.3503	orf19.3503	0.394172086	-1.343102481	2.7433E-13	Ortholog of Candida albicans WO-1: CAWG_05160
C3_06880W_A	orf19.6813	orf19.6813	0.392088886	-1.350747346	0.002092286	Protein of unknown function; Hap43-induced gene

C1_08410C_A	orf19.386	SAM4	0.389404545	-1.360658371	4.11063E-24	Putative S-adenosylmethionine-homocysteine methyltransferase; Hap43-repressed; alkaline induced; Spider biofilm repressed
C1_04850C_A	orf19.757.1	orf19.757.1	0.388498966	-1.364017336	8.85069E-06	Protein of unknown function
C6_03760C_A	orf19.5750	SHM2	0.385399413	-1.375573721	5.25937E-25	Cytoplasmic serine hydroxymethyltransferase; complements glycine auxotrophy of S. cerevisiae shm1 shm2 gly1-1 mutant; antigenic; farnesol-upregulated in biofilm; stationary-phase enriched protein; rat catheter and Spider biofilm repressed
CR_09170C_A	orf19.7313	SSU1	0.384596703	-1.3785817	5.0699E-13	Protein similar to S. cerevisiae Ssu1 sulfite transport protein; Tn mutation affects filamentous growth; regulated by Gcn2 and Gcn4; induced by nitric oxide; Hap43-repressed; Spider and flow model biofilm induced
CR_10570C_A	orf19.7637	YHB4	0.383192745	-1.38385785	4.77825E-20	Protein related to flavohemoglobins; not required for wild-type nitric oxide resistance; has predicted globin, FAD-binding, and NAD(P)-binding domains but lacks some conserved residues of flavohemoglobins; Hap43p-repressed gene
CR_09360W_A	orf19.7331	FCY24	0.382795923	-1.38535263	3.10689E-15	Putative transporter; more similar to S. cerevisiae Tpn1, which is a vitamin B6 transporter, than to purine-cytosine permeases; transcription is regulated by Nrg1; Spider biofilm induced
C7_00280W_A	orf19.7094	HGT12	0.380284719	-1.394848126	7.12045E-19	Glucose, fructose, mannose transporter; major facilitator superfamily; role in macrophage-induced hyphal growth; detected at germ tube plasma membrane by mass spectrometry; Snf3p-induced; 12 probable transmembrane segments

CR_02880W_A	orf19.2846	orf19.2846	0.380160682	-1.395318767	4.1335E-28	Protein of unknown function; Hap43-repressed; induced in core caspofungin response; regulated by yeast-hypha switch; Spider biofilm repressed
C5_01790C_A	orf19.3196	orf19.3196	0.378876096	-1.400201976	0.00031415	Ortholog of Candida albicans WO-1: CAWG_04564
C5_03340W_A	orf19.2645	orf19.2645	0.372167774	-1.425974959	0.009213414	Ortholog of Candida albicans WO-1: CAWG_04710
C3_06790W_A	orf19.6824	TRY6	0.366756368	-1.44710608	8.73532E-05	Helix-loop-helix transcription factor; regulator of yeast form adherence; required for yeast cell adherence to silicone substrate; Spider and F-12/CO2 biofilm induced; repressed by alpha pheromone in SpiderM medium
C4_00010W_A	orf19.362	TLO9	0.364238764	-1.457043624	6.09359E-15	Member of a family of telomere-proximal genes of unknown function; Hap43p-repressed gene
C1_10400C_A	orf19.4910	FGR41	0.363187439	-1.461213787	1.0513E-13	Putative GPI-anchored adhesin-like protein involved in regulation of covering (masking) cell wall glucan; transposon mutation affects filamentous growth; Spider biofilm repressed
CR_08830W_A	orf19.7279.1	orf19.7279.1	0.357599156	-1.483584765	2.6892E-08	Protein of unknown function; Spider biofilm induced
C7_03310W_A	orf19.1344	orf19.1344	0.353560013	-1.499972978	1.09268E-07	Protein of unknown function; fluconazole-induced; Spider biofilm induced
C1_02600W_A	orf19.2948	SNO1	0.350056638	-1.514339731	3.59345E-30	Protein with a predicted role in pyridoxine metabolism; stationary phase protein; regulated by Tup1, Efg1; Spider biofilm induced
CR_05330W_A	orf19.5288.1	orf19.5288.1	0.348986781	-1.518755703	6.36548E-05	Protein of unknown function; Spider biofilm repressed
C5_03130W_A	orf19.4342	SUT1	0.34821161	-1.521963791	1.82737E-26	Zn2Cys6 transcription factor involved in sterol uptake; flow model biofilm induced; Spider biofilm repressed
C7_03190C_A	orf19.5137	orf19.5137	0.342294751	-1.546688926	0.000502141	Protein of unknown function; regulated by Sef1, Sfu1, Hap43; Spider biofilm repressed
C4_07260W_A	orf19.3073	orf19.3073	0.342134774	-1.54736335	2.8327E-38	Protein of unknown function

C1_08400C_A	orf19.385	GCV2	0.340719114	-1.553345211	2.17781E-52	Glycine decarboxylase P subunit; protein of glycine catabolism; repressed by Efg1; Hog1-induced; induced by Rim101 at acid pH; transcript induced in elevated CO2; stationary phase enriched protein
C2_10540W_A	orf19.5326	MIG2	0.339994596	-1.55641628	6.92629E-07	Transcription factor with zinc finger DNA-binding motif, involved in glucose repression; possible ortholog of S. cerevisiae Mig2p
CR_09760W_A	orf19.7548	MED21	0.334289021	-1.580832121	2.80525E-06	Ortholog(s) have transcription coactivator activity, transcription corepressor activity and role in negative regulation of transcription by RNA polymerase II, positive regulation of transcription by RNA polymerase II
C1_00190C_A	orf19.6084	orf19.6084	0.329317695	-1.602448063	8.42076E-06	Protein of unknown function; flow model biofilm induced; Spider biofilm induced
C2_03090C_A	orf19.5789	ADE8	0.321350613	-1.637779869	4.10526E-52	Putative phosphoribosylglycinamide formyl-transferase, enzyme of amino acid biosynthesis pathway; upregulated in biofilm; S. cerevisiae ortholog is Gcn4p regulated; protein enriched in stationary phase yeast-form cultures
C1_04720W_A	orf19.772.1	orf19.772.1	0.304430532	-1.715815039	0.003248163	Ortholog of C. parapsilosis CDC317: CPAR2_105440, Lodderomyces elongisporus NRLL YB-4239: LELG_01504, Pichia stipitis Pignal: psti_CGOB_00118 and Candida tropicalis NEW ASSEMBLY: CTRG1_CGOB_00081
C1_11480W_A	orf19.655	PHO84	0.302413247	-1.725406757	0.003357149	High-affinity phosphate transporter; transcript regulated by white-opaque switch; Hog1, ciclopirox olamine or alkaline induced; caspofungin, stress repressed; required for normal TORC1 function

C1_04450C_A	orf19.6837	FMA1	0.301943283	-1.727650515	7.05848E-28	Putative oxidoreductase; induced by ciclopirox olamine; upregulation correlates with clinical development of fluconazole resistance; Spider biofilm repressed
CR_09180W_A	orf19.7314	CDG1	0.298767431	-1.742905208	9.27989E-22	Putative cysteine dioxygenases; role in conversion of cysteine to sulfite; transcript regulated upon white-opaque switch; rat catheter, Spider and flow model biofilm induced
C2_10080W_A	orf19.1773	RAP1	0.297255222	-1.75022594	6.318E-53	Transcription factor; binds telomeres and regulatory sequences in DNA; involved in telomere maintenance; represses hyphal growth under yeast-favoring conditions; similar to (but shorter than) S. cerevisiae Rap1
C2_05620W_A	orf19.6873.1	KTI11	0.290146952	-1.785144319	3.63203E-07	Zn-ribbon protein; required for synthesis of diphthamide on translation factor eEF2; involved in modification of wobble nucleosides in tRNAs; rat catheter and Spider biofilm induced
C6_01490C_A	orf19.3435	orf19.3435	0.274761066	-1.86375051	6.70423E-07	Ortholog of Candida albicans WO-1: CAWG_05215
C6_03320W_A	orf19.5620	orf19.5620	0.27358588	-1.86993432	6.44128E-60	Stationary phase enriched protein; Gcn4-regulated; induced by amino acid starvation (3-AT), benomyl or in azole-resistant strain that overexpresses MDR1; flow model biofilm induced; rat catheter biofilm repressed; overlaps orf19.5621
C1_02780W_A	orf19.2966	orf19.2966	0.271336289	-1.881846087	6.96608E-30	Predicted dienelactone hydrolase domain; clade-associated gene expression; farnesol-downregulated; rat catheter biofilm repressed
C1_02580W_A	orf19.2946	HNM4	0.258605313	-1.951176177	1.34216E-26	Putative choline permease; fungal-specific (no human or murine homolog)
C1_14120C_A	orf19.7218	RBE1	0.257177587	-1.959163177	1.08496E-18	Pry family cell wall protein; Rim101, Efg1, Ssn6, alkaline repressed; O-glycosylation; no GPI anchor predicted; ketoconazol induced; regulated by Sef1, Sfu1, Hap4; flow

						model biofilm induced; rat catheter and Spider biofilm repressed
C5_01990W_A	orf19.3172	orf19.3172	0.256456675	-1.96321297	7.15579E-29	Ortholog of C. dubliniensis CD36: Cd36_51840, Candida tropicalis NEW ASSEMBLY: CTRG1_05315, Candida tropicalis MYA-3404: CTRG_05315 and Candida albicans WO-1: CAWG_04584
CR_03270W_A	orf19.2397	VHT1	0.25405928	-1.976762934	1.77262E-32	Predicted membrane transporter, involved in biotin import; member of the anion:cation symporter (ACS) family, major facilitator superfamily (MFS); biotin-dependent transcription regulated by Vhr1p; amphotericin B, caspofungin repressed
C5_03480C_A	orf19.6656	orf19.6656	0.244543618	-2.031836279	1.6797E-45	Spermidine transporter; induced in strains from HIV patients with oral candidiasis; alkaline repressed; amphotericin B induced; colony morphology regulated by Ssn6; reduced oral epithelial cell damage by mutant; Spider biofilm induced
CR_07260C_A	orf19.6142	orf19.6142	0.231635606	-2.11007106	2.31078E-05	Ortholog(s) have double-stranded DNA binding activity, role in reciprocal meiotic recombination and condensed nuclear chromosome localization
C1_02520W_A	orf19.2941	SCW4	0.22381035	-2.159651343	4.40352E-26	Putative cell wall protein; substrate for Kex2p processing in vitro; expression regulated by white-opaque switch; alkaline repressed; possibly essential (UAU1 method); flow model biofilm induced; Spider biofilm induced
CR_10110W_A	orf19.7586	CHT3	0.210922753	-2.245213365	1.52183E-38	Major chitinase; secreted; functional homolog of S. cerevisiae Cts1p; 4 N-glycosylation motifs; possible O-mannosylation; putative signal peptide; hyphal-repressed; farnesol upregulated in biofilm; regulated by Efg1p, Cyr1p, Ras1p

C2_03370W_A	orf19.889	THI20	0.193227039	-2.371631102	1.16779E-83	Putative trifunctional enzyme of thiamine biosynthesis, degradation and salvage; Spider biofilm induced
C7_01560C_A	orf19.6570	NUP	0.18612352	-2.425667715	4.98643E-32	Nucleoside permease; adenosine and guanosine are substrates, whereas cytidine, adenine, guanine, uridine, uracil are not; similar to a nucleoside permease of S. pombe; possibly processed by Kex2p
C1_02590C_A	orf19.2947	SNZ1	0.174524908	-2.518495144	3.18701E-62	Stationary phase protein; vitamin B synthesis; induced byyeast-hypha switch, 3-AT or in azole-resistant strain overexpressing MDR1; soluble in hyphae; regulated by Gcn4, macrophage; Spider biofilm induced; rat catheter biofilm repressed
C6_01990W_A	orf19.689	PLB1	0.172990145	-2.531238243	1.86539E-23	Phospholipase B; host cell penetration and virulence in mouse systemic infection; Hog1-induced; signal sequence, N-glycosylation, and Tyr phosphorylation site; induced in fluconazole-resistant strains; rat catheter biofilm repressed
C7_01010W_A	orf19.7013	orf19.7013	0.132712957	-2.913618859	2.34574E-22	Ortholog of C. dubliniensis CD36: Cd36_70940, C. parapsilosis CDC317: CPAR2_300280, Debaryomyces hansenii CBS767: DEHA2F22220g andPichia stipitis Pignal: PICST_32459

[a]**: Red: up-regulated genes; Green: down-regulated genes**

[b]**:** Descriptions are derived from the Candida Genome Database (CGD, http://www.candidagenome.org).

Supplementary Table S4: Result of Gene Set Enrichment Analysis (GSEA)

**Term**	**NES**[a]	***p*-value**	**FDR**[b]
Ca21ChrRx11	-3.447410699	<3.3e-05	<3.3e-05
Ca21ChrRx12	-3.121276747	<3.3e-05	<3.3e-05
Ca21ChrRx10	-3.054824663	<3.3e-05	<3.3e-05
ribosome_CEL	-2.381750221	<3.3e-05	<3.3e-05
structural constituent of ribosome_MOL	-2.342251395	<3.3e-05	<3.3e-05
ribonucleoprotein complex biogenesis and assembly_BIO	-2.204057741	<3.3e-05	0.00134222
translation_BIO	-2.201278315	<3.3e-05	0.001227172
ribosome biogenesis and assembly_BIO	-2.188415903	<3.3e-05	0.001567957
cytosolic part_CEL	-2.188149356	<3.3e-05	0.001420855
cytosolic ribosome (sensu Eukaryota)_CEL	-2.185549238	<3.3e-05	0.001391028
large ribosomal subunit_CEL	-2.183568328	<3.3e-05	0.00132669
HU_6h_dn	-2.146578534	<3.3e-05	0.002944749
Fhl1_TF	-2.144709631	<3.3e-05	0.002857145
OS(2006)_dn	-2.101116742	<3.3e-05	0.006843578
Ifh1_TF	-2.04208324	<3.3e-05	0.019757477
RPS7A_BIND	-2.030467961	<3.3e-05	0.022872647
Tbf1_TF	-2.021572323	<3.3e-05	0.025075826
organellar ribosome_CEL	-2.020993893	<3.3e-05	0.022631497
mitochondrial ribosome_CEL	-2.020993893	<3.3e-05	0.022631497
protein-RNA complex assembly_BIO	-2.003004251	<3.3e-05	0.029182301
MPP10_BIND	-1.989599676	7.40083E-05	0.0345886
RPL3_BIND	-1.988374825	<3.3e-05	0.033746292
small ribosomal subunit_CEL	-1.976547397	7.47328E-05	0.03893817
cytosolic large ribosomal subunit (sensu Eukaryota)_CEL	-1.966911344	0.000441112	0.043426914
NAN1_BIND	-1.954886229	0.000294942	0.049987197
BMS1_BIND	-1.952264482	0.000442576	0.049904298
DIM1_BIND	-1.948864282	0.000652789	0.05053616

Cdc5_4h_dn	-1.94861676	<3.3e-05	0.048912584
RPL17A_BIND	-1.938046107	7.53069E-05	0.055013343
RPS3_BIND	-1.934552551	<3.3e-05	0.055984398
ribosomal subunit assembly_BIO	-1.93310776	0.000870764	0.055302608
nucleolus_CEL	-1.932245155	7.82534E-05	0.054228734
ribosomal large subunit biogenesis and assembly_BIO	-1.930369577	0.000510093	0.053980164
RPL25_BIND	-1.924223938	0.000306373	0.056973286
RPL20A_BIND	-1.924076638	<3.3e-05	0.055468195
NOC4_BIND	-1.918451036	0.000584539	0.058215737
rRNA metabolic process_BIO	-1.916271673	<3.3e-05	0.05833347
RPS22A_BIND	-1.913197605	0.000222965	0.059261898
rRNA processing_BIO	-1.903346575	<3.3e-05	0.065933345
ribosome assembly_BIO	-1.903147928	0.000797333	0.064446078
KRE33_BIND	-1.899360887	7.47719E-05	0.066193166
vitamin metabolic process_BIO	-1.893712016	0.000894588	0.067897597
water-soluble vitamin metabolic process_BIO	-1.893712016	0.000894588	0.067897597
RPL10_BIND	-1.891990881	7.58668E-05	0.0677577
UTP20_BIND	-1.891433967	0.001296083	0.066738969
Cd_dn	-1.890996242	0.000149869	0.06566267
aromatic compound metabolic process_BIO	-1.888125589	0.000733299	0.066682107
nucleolar part_CEL	-1.886111645	0.000818635	0.066980839
eukaryotic 43S preinitiation complex_CEL	-1.883335853	0.000661911	0.067958659
amino acid biosynthetic process_BIO	-1.879043587	<3.3e-05	0.070341107
cellular biosynthetic process_BIO	-1.872112258	<3.3e-05	0.0753806
RPL5_BIND	-1.860131698	7.64584E-05	0.085599838
amine biosynthetic process_BIO	-1.859820994	0.000152138	0.082726841
nitrogen compound	-1.859820994	0.000152138	0.082726841

biosynthetic process_BIO			
PNO1_BIND	-1.858359643	0.001717844	0.082615963
RPP0_BIND	-1.858296409	0.000226912	0.081204303
RPL7B_BIND	-1.856948117	0.000303444	0.081088919
cytokinesis, site selection_BIO	-1.856352343	0.001248898	0.078921702
cellular bud site selection_BIO	-1.856352343	0.001248898	0.078921702
organellar large ribosomal subunit_CEL	-1.855967472	0.001745455	0.076685363
mitochondrial large ribosomal subunit_CEL	-1.855967472	0.001745455	0.076685363
branched chain family amino acid metabolic process_BIO	-1.853858366	0.003525099	0.077382716
pmt4_CAP4-216_up	-1.850244363	0.000742611	0.079509
RPS4A_BIND	-1.849214711	0.000531511	0.079242069
BRX1_BIND	-1.844399734	0.000746491	0.082482509
processing of 20S pre-rRNA_BIO	-1.843596913	0.002681354	0.081981903
amino acid metabolic process_BIO	-1.834853186	<3.3e-05	0.089492738
RPL2B_BIND	-1.832675684	0.001754386	0.090345036
amino acid and derivative metabolic process_BIO	-1.83142315	<3.3e-05	0.090239618
snoRNA binding_MOL	-1.828562016	0.003268923	0.091910337
RPS11A_BIND	-1.823821982	0.001562849	0.095617613
NOC2_BIND	-1.819195179	0.001265917	0.099375113
ECM16_BIND	-1.818360856	0.002707251	0.098889011
NOP1_BIND	-1.812898008	0.00015597	0.103730842
cytosolic small ribosomal subunit (sensu Eukaryota)_CEL	-1.808921498	0.002764239	0.105568484
eukaryotic 48S initiation complex_CEL	-1.808921498	0.002764239	0.105568484
UTP15_BIND	-1.806934644	0.002786128	0.106472424
RPS5_BIND	-1.799140456	0.000760225	0.114306449
EMG1_BIND	-1.798335268	0.005492938	0.113829513

small nucleolar ribonucleoprotein complex_CEL	-1.794407767	0.005363096	0.117299036
OS(hog1)_dn	-1.791671572	0.001277523	0.119199369
RPS1B_BIND	-1.788705034	0.000684984	0.121352153
HS_dn	-1.786307079	<3.3e-05	0.122963218
RPS13_BIND	-1.784698506	0.00338534	0.123524902
RPL11B_BIND	-1.784262999	0.001864837	0.122608556
RPL1A_BIND	-1.780663627	0.000898742	0.125697047
XS_dn	-1.780542216	<3.3e-05	0.124395873
hypoxia_synnott_dn	-1.779838167	0.000239006	0.123819229
RPL4B_BIND	-1.776222204	0.002665877	0.12708615
anion transport_BIO	-1.773865259	0.009821933	0.128686619
aromatic amino acid family biosynthetic process_BIO	-1.772824489	0.008071368	0.12727852
aromatic compound biosynthetic process_BIO	-1.772824489	0.008071368	0.12727852
cytokinetic process_BIO	-1.77105936	0.001269604	0.128211257
ribosomal large subunit assembly and maintenance_BIO	-1.768118785	0.005565863	0.130674582
BiofilmBatch_dn	-1.767236434	0.000158541	0.130449901
methyltransferase activity_MOL	-1.76658874	0.002270064	0.129917225
transferase activity, transferring one-carbon groups_MOL	-1.7625471	0.002348969	0.13386574
one-carbon compound metabolic process_BIO	-1.761368194	0.006703669	0.134073074
tRNA modification_BIO	-1.759444499	0.009029345	0.135275058
UTP30_BIND	-1.758603662	0.006376812	0.134972655
QCR6_BIND	-1.753385082	0.010022034	0.140729882
RRP9_BIND	-1.753375388	0.005595524	0.139365968
RPS15_BIND	-1.751225922	0.005273423	0.140944697
RPL36B_BIND	-1.74856953	0.002538829	0.143176378
HAS1_BIND	-1.747537978	0.001228784	0.14317198
RPL7A_BIND	-1.745075461	0.00327381	0.145242465

RPL14B_BIND	-1.744230394	0.005859799	0.145075153
aromatic amino acid family metabolic process_BIO	-1.743827514	0.011478682	0.144283214
Cdc5_9h_dn	-1.740610886	0.000699844	0.147455224
RPS1A_BIND	-1.737033089	0.001646091	0.151179217
RLP7_BIND	-1.734843753	0.002526754	0.152950627
NOG1_BIND	-1.726750329	0.002116882	0.163446647
axial cellular bud site selection_BIO	-1.722174523	0.013312451	0.169073921
GNP1_BIND	-1.720184983	0.009580491	0.17059208
NIP7_BIND	-1.718380969	0.004580378	0.172040121
MRP7_BIND	-1.718019975	0.011369324	0.171114942
RPL16A_BIND	-1.715026431	0.001954593	0.174302113
DIP2_BIND	-1.713779957	0.010812746	0.174782674
amine metabolic process_BIO	-1.713464502	8.088E-05	0.173822499
RPL13B_BIND	-1.713429954	0.00580072	0.172436208
NMD5_BIND	-1.711800125	0.006652376	0.173540257
GAS5_BIND	-1.711630493	0.016143879	0.172370238
RPL28_BIND	-1.711423143	0.007511046	0.171289875
translational initiation_BIO	-1.710270241	0.008754012	0.171659295
RRP12_BIND	-1.710011747	0.002755231	0.170701865
SSM4_BIND	-1.709226825	0.00797853	0.170565739
TIF6_BIND	-1.709178214	0.00300368	0.169302639
serine family amino acid metabolic process_BIO	-1.7001387	0.013498072	0.18201493
RNA methyltransferase activity_MOL	-1.694793733	0.018193357	0.189296704
UTP21_BIND	-1.692260302	0.01132511	0.192033182
RPL18B_BIND	-1.68874254	0.010477299	0.196444359
RPL6A_BIND	-1.687700178	0.003635262	0.196756123
RPS17B_BIND	-1.684010391	0.006932538	0.201502893
translation initiation factor activity_MOL	-1.6832144	0.01465704	0.201357751
EBP2_BIND	-1.681953749	0.008651399	0.202072333
RPS4B_BIND	-1.681558973	0.003947468	0.201261206
cell separation during	-1.681129074	0.02070922	0.200520703

cytokinesis_BIO			
UPS2_BIND	-1.679923893	0.018408802	0.201177009
aspartate family amino acid metabolic process_BIO	-1.676954854	0.009506264	0.204852074
ace2_yeast_up	-1.676065316	0.011354653	0.204941636
carboxylic ester hydrolase activity_MOL	-1.672178002	0.017513761	0.210346538
TRM7_BIND	-1.668080447	0.016639408	0.216259636
RPL1B_BIND	-1.665983762	0.014615883	0.21854275
SLS1_BIND	-1.664963708	0.01389293	0.218872678
RPS8B_BIND	-1.664961067	0.016046259	0.217367587
glucose catabolic process_BIO	-1.663928518	0.01876348	0.217775594
ENP2_BIND	-1.662409657	0.015445402	0.218999153
cytokinesis_BIO	-1.662038492	0.002983476	0.21818889
RPL20B_BIND	-1.661387128	0.006348969	0.217879548
IMP3_BIND	-1.660496789	0.020101219	0.218016857
BUD21_BIND	-1.659336977	0.003712121	0.218625235
mitochondrial membrane part_CEL	-1.655729091	0.010535622	0.223709843
RPL16B_BIND	-1.654702448	0.006234039	0.224108771
mitochondrial matrix_CEL	-1.654676612	0.001229256	0.221262074
mitochondrial lumen_CEL	-1.654676612	0.001229256	0.221262074
LOS1_BIND	-1.653497379	0.013270768	0.222005359
GCN4_only_up	-1.652757153	0.010615866	0.221928091
LST4_BIND	-1.649888469	0.00820773	0.225675816
AIM27_BIND	-1.644821525	0.026274566	0.233650015
regulation of translation_BIO	-1.643779996	0.02469311	0.234176493
ATS1_BIND	-1.641824397	0.018474417	0.236344989
TUF1_BIND	-1.641255256	0.013238214	0.235937851
SUV3_BIND	-1.640380701	0.023404709	0.236123202
PRT1_BIND	-1.639343476	0.011670581	0.236667293
NAM7_BIND	-1.639194479	0.004216498	0.235517511
anion transporter activity_MOL	-1.638387884	0.025693226	0.235605898
UTP6_BIND	-1.636641121	0.024205641	0.237450607
nucleobase metabolic	-1.632963247	0.024799886	0.243023145

process_BIO			
hydro-lyase activity_MOL	-1.628887617	0.023589302	0.249456511
zap1_up	1.648566338	0.000449817	0.239910916
plasma membrane_CEL	1.651518054	0.000111396	0.237521764
FLC1_BIND	1.654099885	0.017169258	0.235992734
SRS2_BIND	1.656403266	0.004319654	0.235204297
protein targeting to vacuole_BIO	1.660607041	0.007922373	0.230313807
di-, tri-valent inorganic cation homeostasis_BIO	1.662559583	0.006880175	0.230380879
telomere organization and biogenesis_BIO	1.665830468	0.019825736	0.227730808
telomere maintenance_BIO	1.665830468	0.019825736	0.227730808
SGS1_BIND	1.672081507	0.00257732	0.223098817
PDR12_BIND	1.674342872	0.012003234	0.222669648
urea cycle intermediate metabolic process_BIO	1.677624547	0.014300947	0.22023579
single-species biofilm formation_BIO	1.678447375	0.016556291	0.223208223
small GTPase mediated signal transduction_BIO	1.683662864	0.006245408	0.216521437
White_up	1.683679485	0.001928246	0.221295335
Tec1_TF	1.685007178	0.014028056	0.223374345
Swi4p_TFMOTIF	1.687908779	0.008009858	0.222053895
YGR130C_BIND	1.69003476	0.010343324	0.222558302
Cd_up	1.692567651	0.001118042	0.222279413
Efg1_Hyphae_dn	1.695474231	0.008520289	0.221269338
cell surface_CEL	1.700005133	0.000403179	0.216720016
cell projection_CEL	1.718094463	0.010787963	0.184123453
mating projection_CEL	1.718094463	0.010787963	0.184123453
transition metal ion homeostasis_BIO	1.727619015	0.00373317	0.175577988
single-species biofilm formation on inanimate substrate_BIO	1.731919479	0.009803922	0.172306949
intraspecies interaction between organisms_BIO	1.731919479	0.009803922	0.172306949

OS(2006)_up	1.731925559	5.6699E-05	0.18273741
second-messenger-mediated signaling_BIO	1.735235195	0.00737798	0.181778084
MGS1_BIND	1.742564888	0.005900255	0.17299646
tsa1_dn	1.759409593	0.006484105	0.147583731
nucleotide-excision repair_BIO	1.763462527	0.004113175	0.145620186
XS_up	1.771792178	0.000233659	0.13681541
biological adhesion_BIO	1.772293768	0.002672011	0.141052411
FMP45_BIND	1.773677241	0.004799192	0.144206157
RAD17_BIND	1.775802817	0.002983984	0.146281523
biofilm formation_BIO	1.777558782	0.004608295	0.149267173
HXT1_BIND	1.77830631	0.00497915	0.15441905
HU_2h_up	1.788463542	0.000114877	0.142893077
adhesion to other organism during symbiotic interaction_BIO	1.789020849	0.003420683	0.148649523
adhesion to host_BIO	1.789020849	0.003420683	0.148649523
Zap1_TF	1.793030551	0.00089955	0.156543652
G-protein coupled receptor protein signaling pathway_BIO	1.794840047	0.00403401	0.16165658
SIZ1_BIND	1.79812665	0.00323685	0.164376807
meiosis_BIO	1.827370188	0.000119055	0.12023775
meiotic cell cycle_BIO	1.827370188	0.000119055	0.12023775
M phase of meiotic cell cycle_BIO	1.827370188	0.000119055	0.12023775
cyclic-nucleotide-mediated signaling_BIO	1.883882221	0.001139168	0.066639271
cAMP-mediated signaling_BIO	1.883882221	0.001139168	0.066639271
REV3_BIND	1.915324674	0.000307882	0.048473784
HU_6h_up	1.967391381	<3.3e-05	0.021907572
hypoxia_synnott_up	1.98121655	<3.3e-05	0.018883528
Late Biofilm_up	1.98373467	<3.3e-05	0.020281005
APN1_BIND	1.99816088	6.17436E-05	0.017526758
cdc5_9h_up	2.015975634	<3.3e-05	0.014474269

Ras1_Yeast_up	2.021607336	<3.3e-05	0.015293001
response to endogenous stimulus_BIO	2.024496692	<3.3e-05	0.017798242
RHE30_up	2.11514712	<3.3e-05	0.003017459
response to DNA damage stimulus_BIO	2.120850214	<3.3e-05	0.003974464
DNA repair_BIO	2.137216774	<3.3e-05	0.005312908

[a]: NES, normalized enrichment score [b]: FDR, false discovery rate

Supplementary Table S5: DEGs in the *rap1*Δ/Δ mutant that are located in the subtelomeric region[a]

**Up-regulated genes**				
**Gene name**	**Fold change**[b]	**log2 Fold change**	**Adjusted *p*-value (padj)**	**Description**
NRG2	3.430238043	1.778308696	2.04312E-11	Transcription factor; transposon mutation affects filamentous growth
MAL2	2.280141452	1.189123327	3.00694E-16	Alpha-glucosidase; hydrolyzes sucrose for sucrose utilization; transcript regulated by Suc1, induced by maltose, repressed by glucose; Tn mutation affects filamentous growth; upregulated in RHE model; rat catheter and Spider biofilm induced
RDH54	1.922998784	0.943357851	2.53177E-14	Putative DNA-dependent ATPase with a predicted role in DNA recombination and repair; transcriptionally

				induced by interaction with macrophage
TLO11	1.816858092	0.86144574	0.026139655	Member of a family of telomere-proximal genes of unknown function; may be spliced in vivo
TLO8	1.785482159	0.836313718	1.36128E-08	Member of a family of telomere-proximal genes of unknown function; may be spliced in vivo
TLO4	1.661413562	0.732411236	0.004031274	Member of a family of telomere-proximal genes of unknown function; transcript induced in an RHE model of oral candidiasis; Hap43-repressed
**Down-regulated genes**				
**Gene name**	**Fold change[a]**	**log2 Fold change**	**Adjusted *p*-value (padj)**	**Description**
IAH1	0.632831366	-0.660106986	5.60228E-07	Protein similar to S. cerevisiae Iah1p, which is involved in acetate metabolism; mutation confers

				hypersensitivity to tunicamycin; transposon mutation affects filamentous growth
ATP16	0.558172521	-0.841216994	8.02275E-13	Subunit of the mitochondrial F1F0 ATP synthase; sumoylation target; protein newly produced during adaptation to the serum; Spider biofilm repressed
INO1	0.413382669	-1.274450189	1.82787E-34	Inositol-1-phosphate synthase; antigenic in human; repressed by farnesol in biofilm or by caspofungin; upstream inositol/choline regulatory element; glycosylation predicted; rat catheter, flow model induced; Spider biofilm repressed
TLO9	0.364238764	-1.457043624	6.09359E-15	Member of a family of telomere-proximal genes of

				unknown function; Hap43p-repressed gene

[a]: Genes located in the subtelomeric region are adapted from Dunn and Anderson 2019, Genes (Basel), 10(11):866.

[b]: Red: up-regulated genes; Green: down-regulated genes

Unedited western blot images

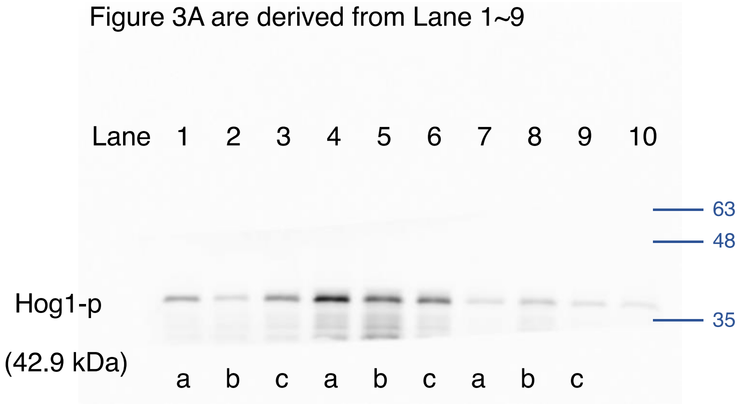

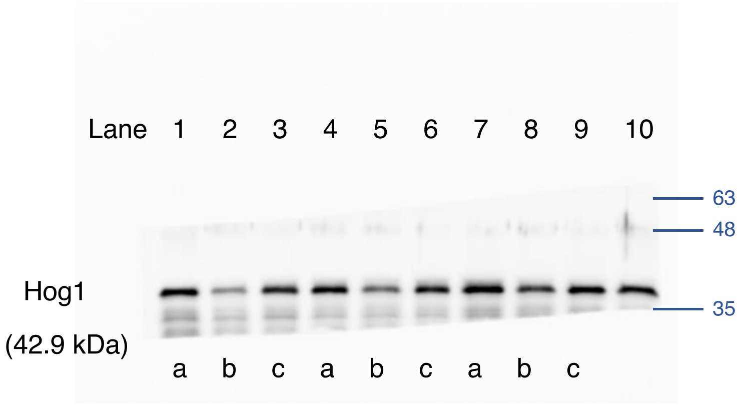

a: SC5314 b: *rap1Δ/Δ* c: *rap1Δ::RAP1/ rap1Δ::RAP1*

Figure 4C are derived from Lane 1~4

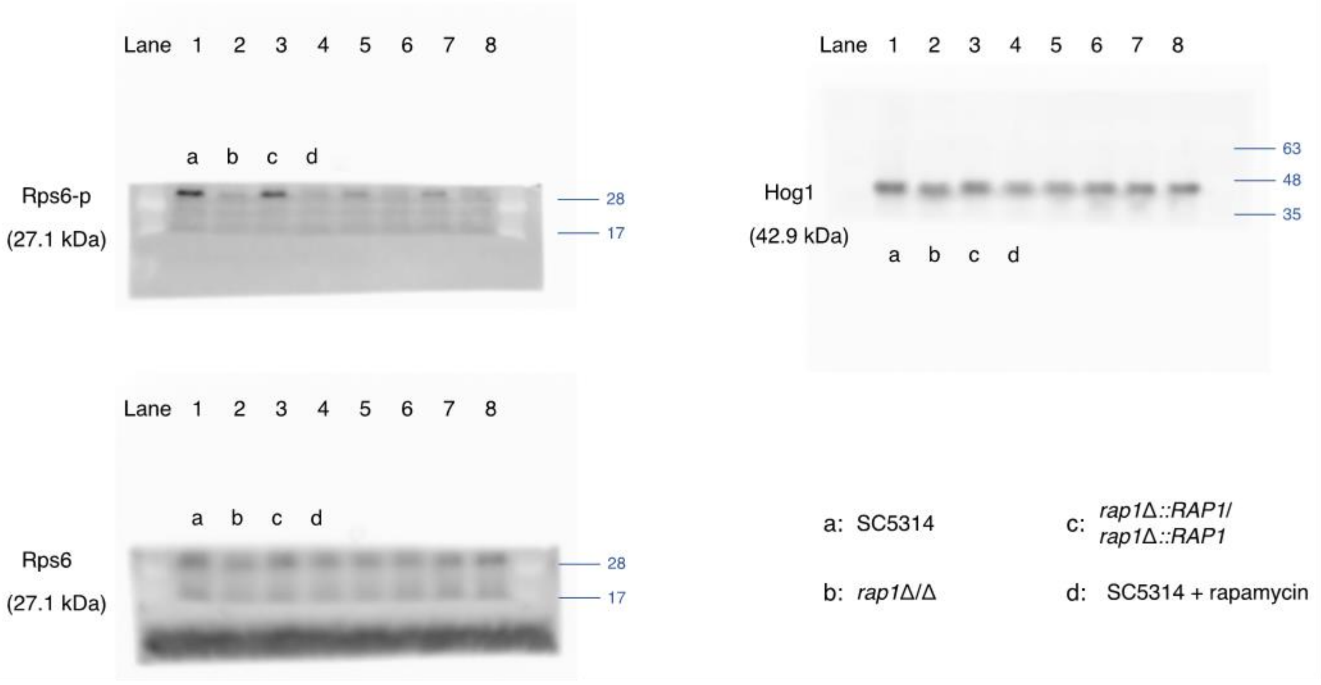

Supplement: Supplementary Tables S1-S5 and Supplementary Material [file BSR-2024-0689_supp.pdf]
